# Supplementary material for: Avapritinib-based SAR studies unveil a binding pocket in KIT and PDGFRA
Source: Nat Commun. 2024 Jan 2;15:63. doi: 10.1038/s41467-023-44376-8 (PMC10761696; doi:10.1038/s41467-023-44376-8)
Supplement: Supplementary file 1 — Supplementary Information [file 41467_2023_44376_MOESM1_ESM.pdf]

Supplementary Information

Avapritinib-based SAR studies unveil a binding pocket in KIT and PDGFRA

A. Teuber[1,‡], T. Schulz[1,‡], B. Fletcher[2], R. Gontla[1], T. Mühlenberg[2], M.-L. Zischinsky[3], J. Niggenaber[1], J. Weisner[1], S. B. Kleinbölting[1], J. Lategahn[1], S. Sievers[4], M. P. Müller[1], S. Bauer,[2] and D. Rauh*,[1]

[1] Department of Chemistry and Chemical Biology, TU Dortmund University and Drug Discovery Hub Dortmund (DDHD), Zentrum für Integrierte Wirkstoffforschung (ZIW), Otto-Hahn-Strasse 4a, 44227 Dortmund, Germany

[2] Department of Medical Oncology and Sarcoma Center, West German Cancer Center, University Duisburg-Essen, Medical School, Essen, Germany

[3] Lead Discovery Center GmbH, Otto-Hahn-Strasse 15, 44227 Dortmund, Germany

[4] Compound Management and Screening Center, Max Planck Institute of Molecular Physiology, Dortmund, Germany

‡These authors contributed equally

*E-Mail: daniel.rauh@tu-dortmund.de

Table of Contents

Supplementary Figures and Tables **S4-S12**

Supplementary Figure 1 S4

Supplementary Figure 2 S5

Supplementary Figure 3 S5

Supplementary Figure 4 S6

Supplementary Figure 5 S6

Supplementary Figure 6 S7

Supplementary Figure 7 S8

Supplementary Figure 8 S9

Supplementary Table 1 S10

Supplementary Table 2 S11

Supplementary Table 3 S12

Supplementary Methods **S13-S20**

Supplementary Table 4 S13

Supplementary Table 5 S13

Supplementary Table 6 S14

Supplementary Table 7 S15

Supplementary Table 8 S16

Supplementary Table 9 S17

Supplementary Table 10 S18

Supplementary Table 11 S19

Supplementary Table 12 S20

Supplementary Table 13 S21

Synthetic Procedures S22

Supplementary Note 1: NMR and mass spectra for final compounds **S30-S49**

Supplementary Note 2: Abbreviations **S50**

References **S50**

Supp. Fig. and Tab.

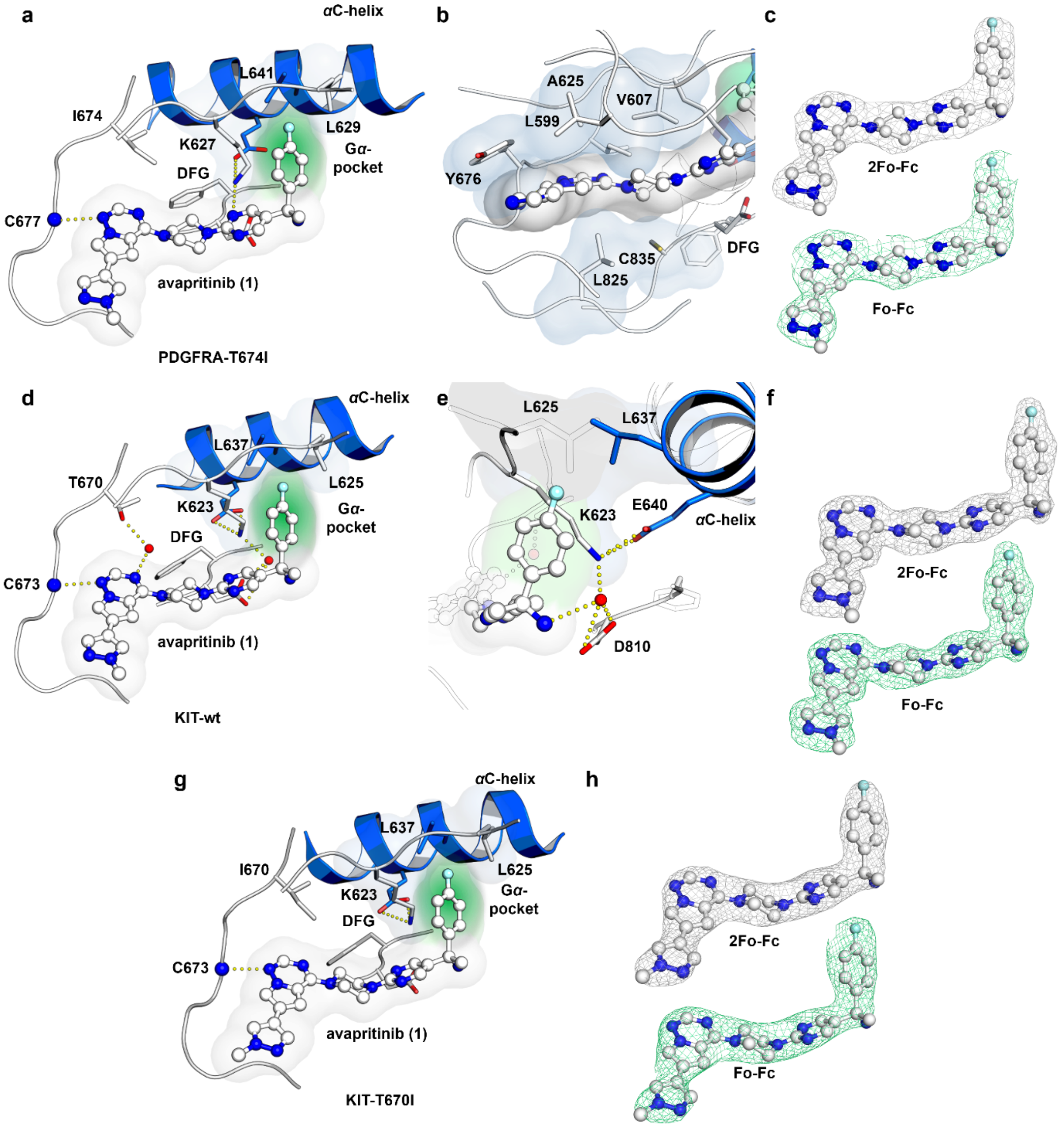

Supp. Fig. 1 Co-crystal structures of avapritinib (1) bound to PDGFRA-T674I (a), KIT-wt (d) and KIT-T670I (g). a Complex crystal structure of **1** bound to PDGFRA-T674I, which shows direct interactions between **1**, the hinge regions Cys677 and the catalytic lysine Lys629 (PDB-ID: 8PQH). **b** Representation of further interactions of **1** with Leu599, Val607, Ala625, Tyr676, Leu825 and Cys835. **c** Corresponding |2Fo-Fc|- (r.m.s.d. = 1.0) and simulated annealing |Fo-Fc| omit (r.m.s.d. = 2.8) electron density maps of **1** bound to PDGFRA-T674I (PDB-ID: 8PQH). **d** Complex crystal structure of **1** bound to KIT-wt. Herein a water-mediated interaction between the N4 of the pyrrolotriazine scaffold of **1** and Thr670 is clearly visible in this structure (PDB-ID: 8PQ9). **e** Representation of the water-mediated interaction of the free amine of **1** bound to KIT-wt, Lys623 and Asp810. **f** Corresponding |2Fo-Fc|- (r.m.s.d. = 1.0) and simulated annealing |Fo-Fc| omit (r.m.s.d. = 2.8) electron density maps of **1** bound to KIT-wt (PDB-ID: 8PQ9). **g** Complex crystal structure of **1** bound to KIT-T670I. It is seen that the isoleucine of Ile670 replaces a water molecule observed in the wt structures, leading to drug resistance (PDB-ID: 8PQG). **h** Corresponding |2Fo-Fc|- (r.m.s.d. = 1.0) and simulated annealing |Fo-Fc| omit (r.m.s.d. = 2.8) electron density maps of **1** bound to KIT-T670I (PDB-ID: 8PQG).

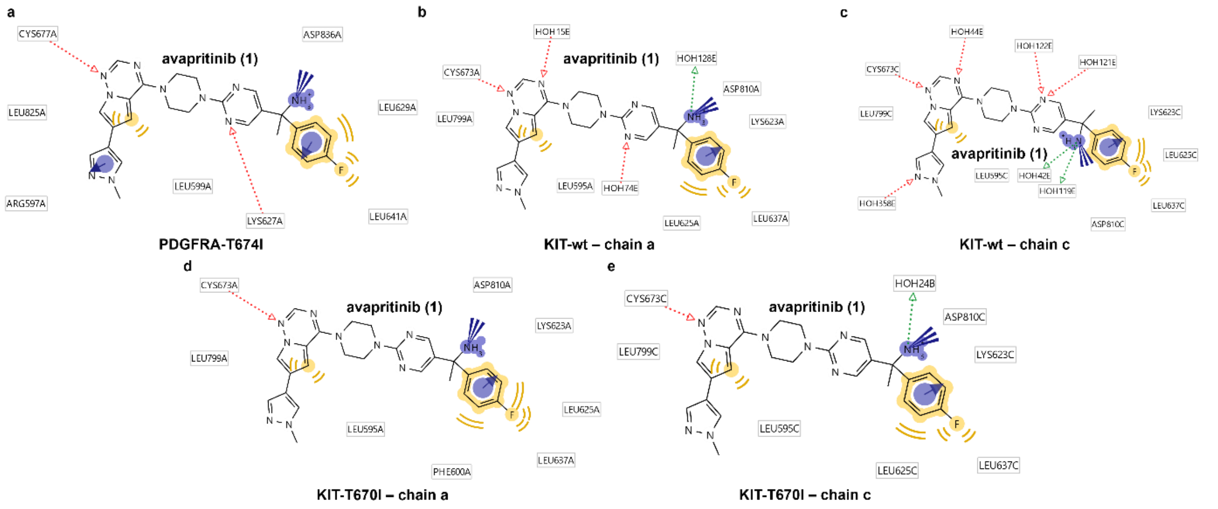

Supp. Fig. 2 Representation of pharmacophoric features of avapritinib bound to PDGFRA-T674I (a), KIT-wt (b, c) and -T670I (d, e). a 2D pharmacophore model of avapritinib (**1**) bound to PDGFRA-T674I. **b**, **c** 2D pharmacophore model of avapritinib (**1**) bound to KIT-wt. **d**, **e** 2D pharmacophore model of avapritinib (**1**) bound to KIT-T670I. LigandScout (v. 4.4.8) was used to generate pharmacophore models and the corresponding 2D visualization.

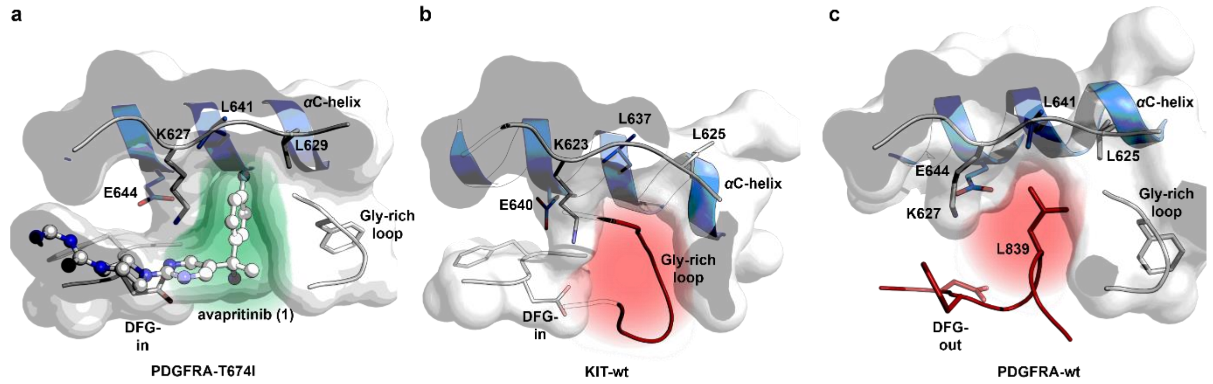

Supp. Fig. 3 Crystal structures explain and underline addressing of the sub-pocket bound by 1. **a** Representation of the Gα-pocket addressed by **1**. This sub-pocket is formed by the amino acids Phe604, Lys627, Leu629, and Leu641 (PDB-ID: 8PQH)). **b** Representation of active KIT-wt bound to ADP in a DFG-in conformation (PDB-ID: 1PKG), where the activation loop (AL) is rotated into the proteins backpocket, so ATP can be bound and the kinase domain gets activated. **c** Apo crystal structure of PDGFRA-wt in the auto-inhibited DFG-out conformation (PDB-ID: 8PQJ). Leu839 of the DFGLARDI motif protrudes into a pocket between the αC helix and the Gly-rich loop. In addition, the juxtamembrane domain (JMD, red) is protuding into the back pocket of the kinase domain.

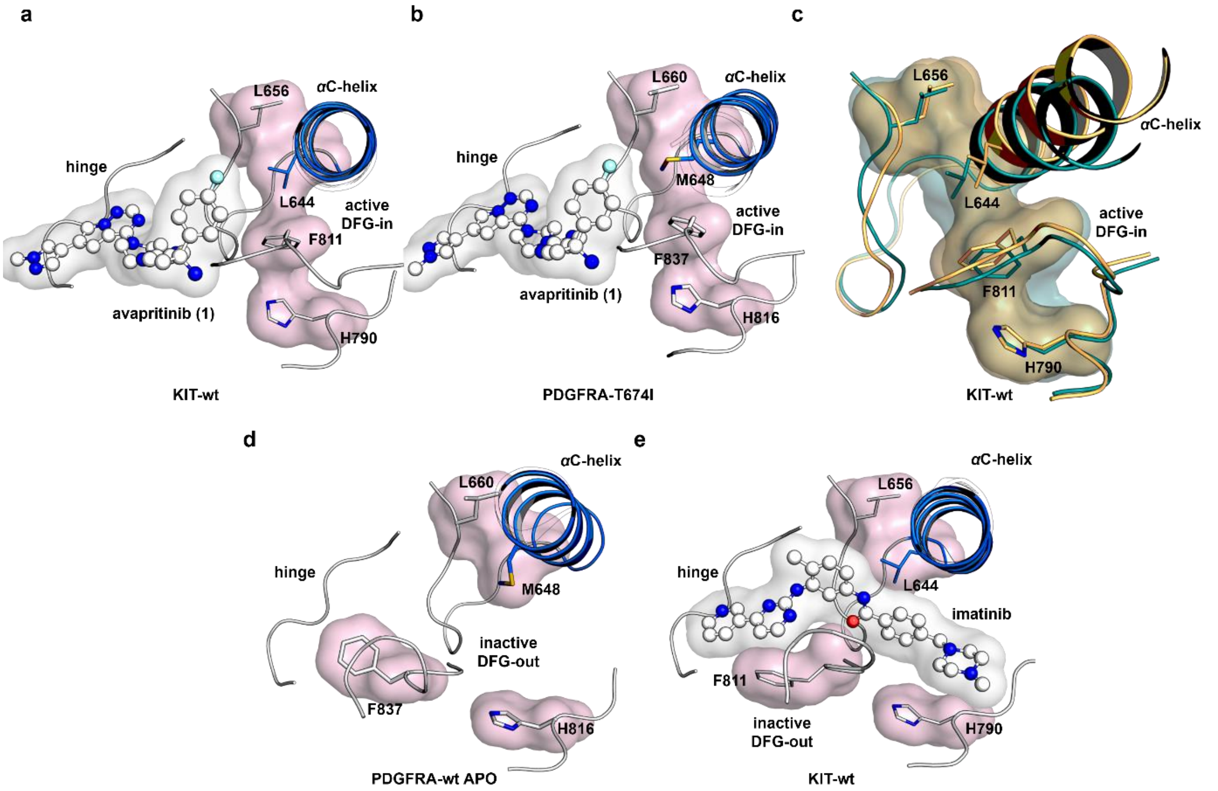

Supp. Fig. 4 Illustration of the R-spine assembly. a Illustration of avapritinib (**1**) bound to KIT-wt in the DFG-in conformation. Phe811 points inwards the backpocket, so the R-spine can assemble. **b** illustration of PDGFRA-T674I bound to avapritinib (**1**) in a DFG-in conformation. Phe837 points towards the backpocket of the protein, so the R-spine can assemble. **c** alignment of active KIT-wt (PDB-ID: 1PKG, green) and KIT-wt (orange) bound to avapritinib (**1**, ligand not shown). **d** illustration of inactive KIT-wt in a DFG-out conformation- Phe811 points inwards the ATP-binding site, so the R-spine cannot assemble. **e** Complex crystal structure of KIT-wt bound to imatinib (PDB-ID: 1T46) in a DFG-out conformation. The R-spine cannot assemble as Phe811 is rotated inwards the ATP-binding pocket.

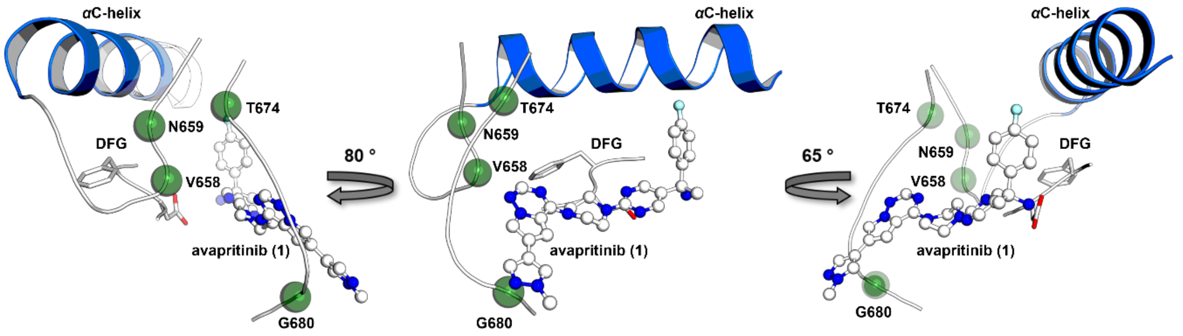

Supp. Fig. 5 Overview of emerging resistance mutations. Illustration of occurring resistance mutations within the ATP-binding pocket in PDGFRA-T674I bound to avapritinib (V658A, N659K, T674I/R, G680R) visualized from different angles (PDB-ID: 8PQH).

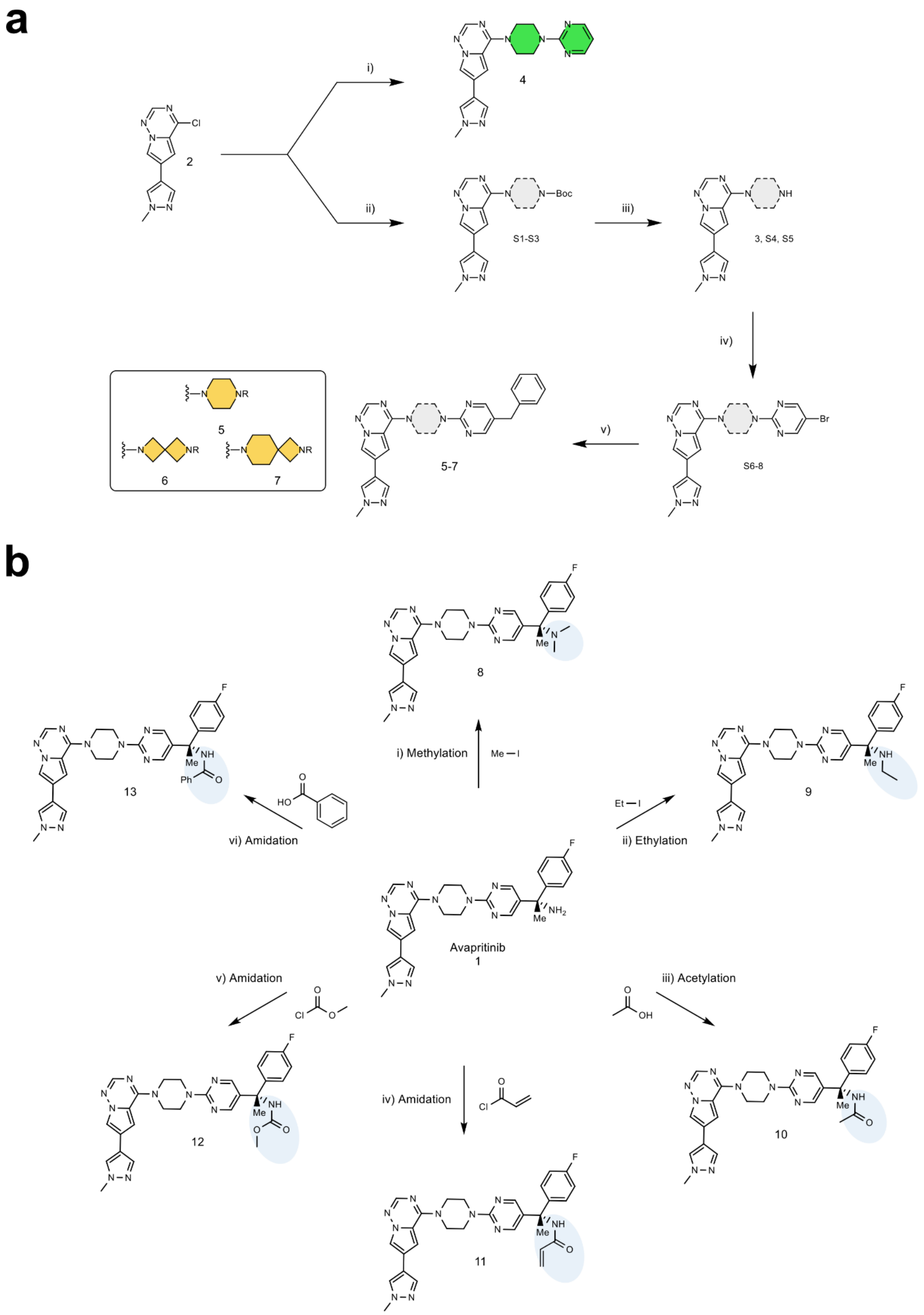

Supp. Fig. 6 Synthesis schemes of avapritinib-based inhibitors. (a) Linear synthesis route for the preparation of spiro containing derivatives. i)2-(piperazin-1-yl)pyrimidine, TEA, dry THF, rt 6 h, 76 %; ii) Boc protected di-amine, TEA, dry THF, rt, 6 h-ovn, 66-98 %; iii) HCl, DCM, rt, ovn, 63 %-quant.; iv) 5-bromo-2-chloropyrimidine, TEA, EtOH, 60 °C, ovn, 63-85 %; v) benzylboronic acid pinacol ester, Pd(dppf)Cl_2, Cs_2CO_3, Dioxane/water (5:1), 1 h, 120 °C, µw, 54-72 %. **(b)** Late-stage functionalizations of avapritinib. i) MeI, DIPEA, dry THF, 3 h, rt, 77 %; ii) EtI, NaH, dry THF, 8 h, rt, 80 %; iii) acetyl chloride, dry THF, 3 h, rt, 89 %; iv) acryloyl chloride, dry THF, 3 h, rt, 90 %; v) methyl chloroformate, K_2CO_3, dry DCM, 5 h, rt, 84 %; vi) benzoic acid, HATU, TEA, dry DCM, 14 h, rt, 85 %.

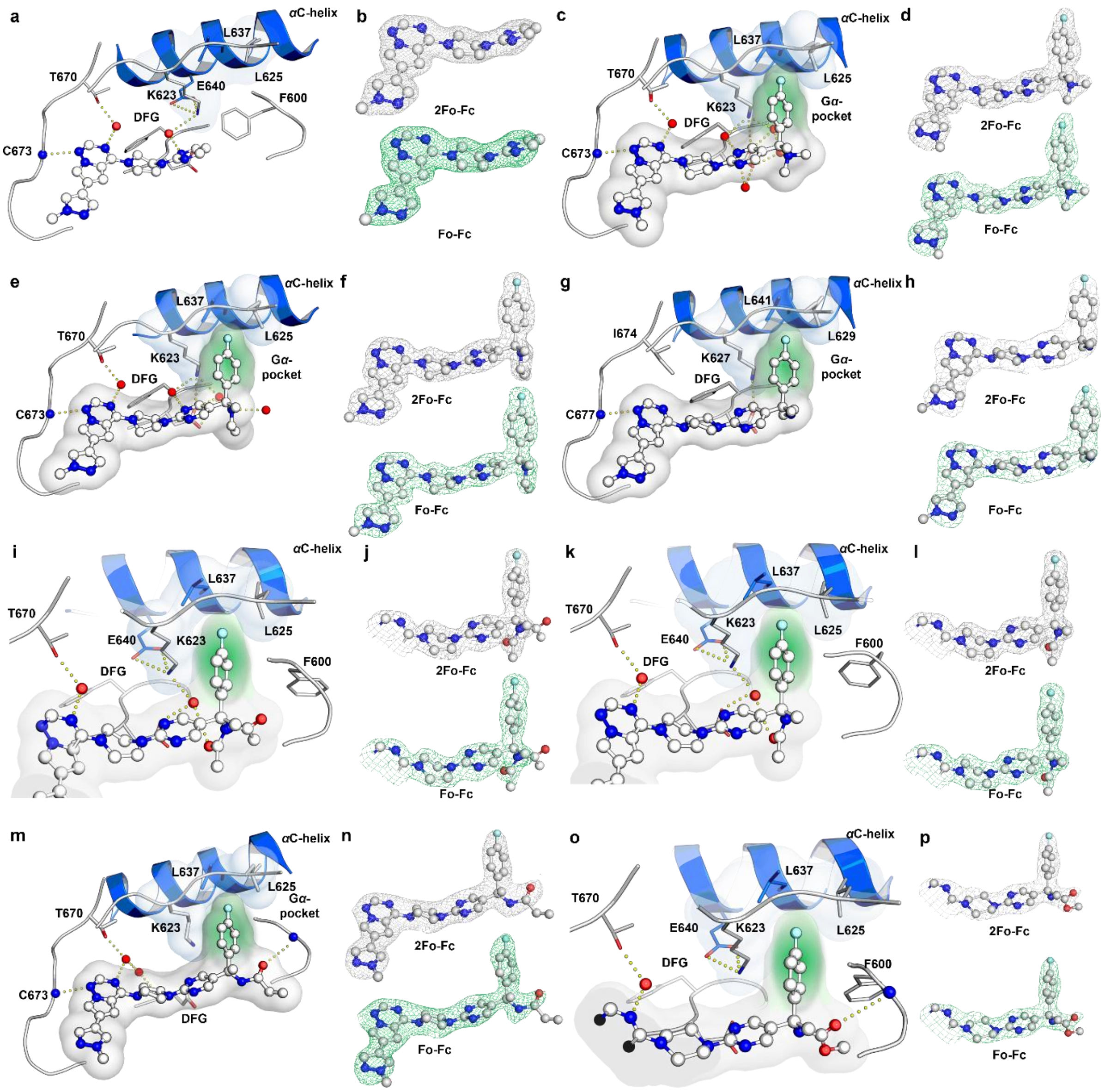

Supp. Fig. 7 Complex crystal structures of 4 and 8-12 bound to KIT-wt and 9 bound to PDGFRA-T674I. a Complex crystal structure of **4** bound to KIT-wt. Without the fluorobenzene moiety (**4**), Phe600 of the Gly-rich loop is rotated inwards the binding pocket so that the G*α*-pocket is covered by the side chain of the amino acid (PDB-ID: 8PQA). **b** Corresponding |2Fo-Fc|- (r.m.s.d. = 1.0) and simulated annealing |Fo-Fc| omit (r.m.s.d. = 2.8) electron density maps of **4** bound to KIT-wt (PDB-ID: 8PQA). **c** Complex crystal structure of **8** bound to KIT-wt (PDB-ID: 8PQB). In the binding pocket, a large water network, connecting protein and ligand is observed. **d** Corresponding |2Fo-Fc|- (r.m.s.d. = 1.0) and simulated annealing |Fo-Fc| omit (r.m.s.d. = 2.8) electron density maps of **8** bound to KIT-wt (PDB-ID: 8PQB). **e** Complex crystal structure of **9** bound to KIT-wt (PDB-ID: 8PQC). **f** Corresponding |2Fo-Fc|- (r.m.s.d. = 1.0) and simulated annealing |Fo-Fc| omit (r.m.s.d. = 2.8) electron density maps of **9** bound to KIT-wt (PDB-ID: 8PQC). **g** Complex crystal structure of **9** bound to PDGFRA-T674I. Interactions between ligand and protein can only be identified between Cys677 of the hinge region and the pyrrolotriazine scaffold N2 nitrogen and between the pyrimidine core and N*ε* of the catalytic Lys627 (PDB-ID: 8PQI). **h** Corresponding |2Fo-Fc|- (r.m.s.d. = 1.0) and simulated annealing |Fo-Fc| omit (r.m.s.d. = 2.8) electron density maps of **9** bound to PDGFRA-T674I (PDB-ID: 8PQI). **i, k** Complex crystal structures of **10** bound to KIT-wt (PDB-ID: 8PQD). The asymmetric units of the crystal structure show different flexibilities concerning the acetyl group attached to the free amine. In addition, a slight reorientation of the Gly-rich loop and the inhabited Phe600 can be observed. **j, l** Corresponding |2Fo-Fc|- (r.m.s.d. = 1.0) and simulated annealing |Fo-Fc| omit (r.m.s.d. = 2.8) electron density maps of **10** bound to KIT-wt in the two asymmetric units (PDB-ID: 8PQD). **m** Complex crystal structure of **11** bound to KIT-wt indicating an additional hydrogen bond between the acrylamides oxygen and the backbone nitrogen of Phe600, which is located in the Gly-rich loop (PDB-ID: 8PQE). **n** Corresponding |2Fo-Fc|- (r.m.s.d. = 1.0) and simulated annealing |Fo-Fc| omit (r.m.s.d. = 2.8) electron density maps of **11** bound to KIT-wt (PDB-ID: 8PQE). **o** Complex crystal structure **12** bound to KIT-wt, revealing an additional interaction between the carbonyl oxygen of the carbamate and the backbone of Phe600 of the Gly-rich loop, which leads to a slight reorientation of the Gly-rich loop towards the ligand binding pocket (PDB-ID: 8PQF). **p** Corresponding |2Fo-Fc|- (r.m.s.d. = 1.0) and simulated annealing |Fo-Fc| omit (r.m.s.d. = 2.8) electron density maps of **12** bound to KIT-wt (PDB-ID: 8PQF).

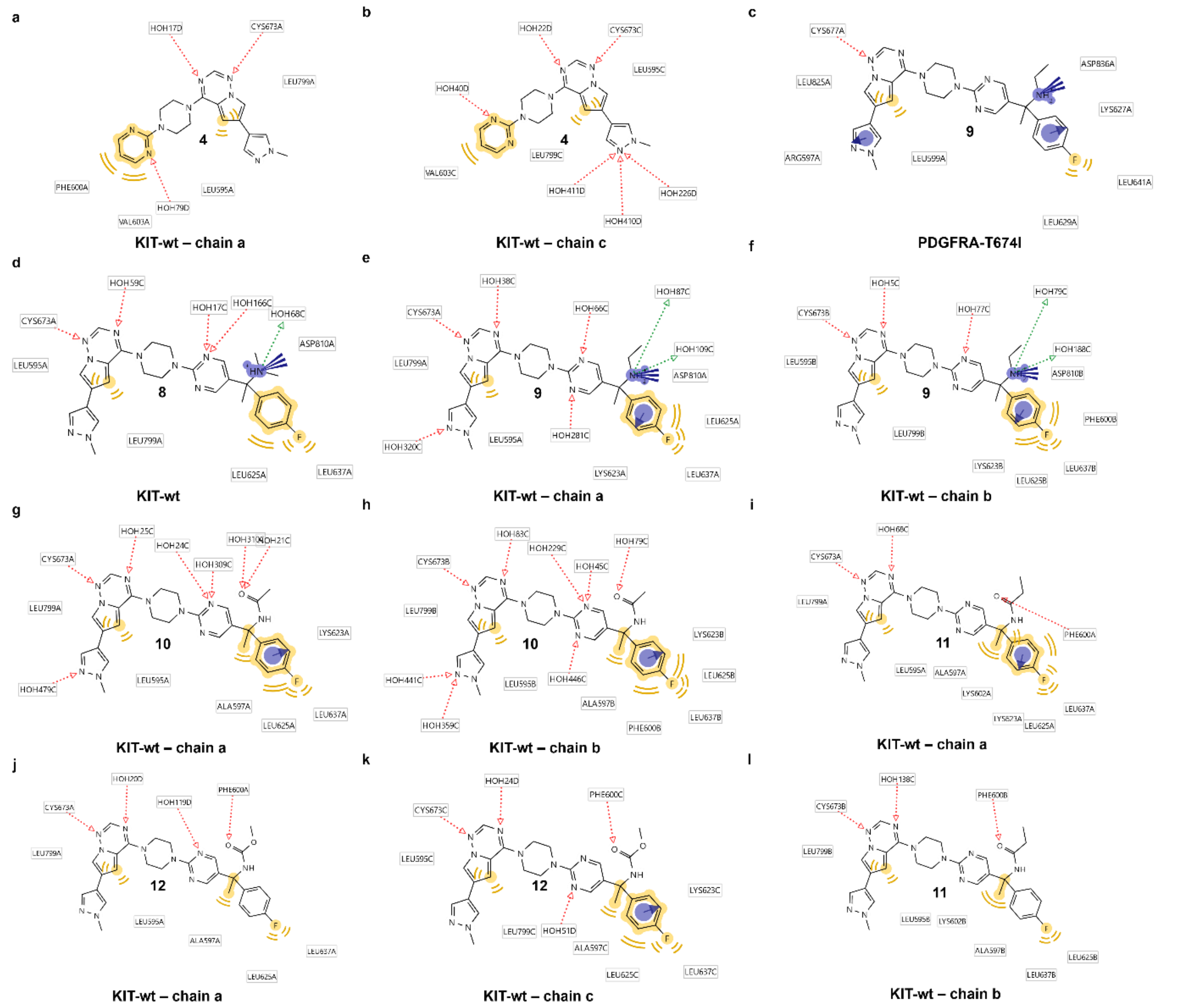

Supp. Fig. 8 Representation of pharmacophoric features of co-crystallized inhibitors 4 and 8-12 bound to KIT-wt and 9 bound to PDGFRA-T674I. a, b 2D pharmacophore model of **4** bound to KIT-wt. **c** 2D pharmacophore model of **9** bound to PDGFRA-T674I. **d** 2D pharmacophore model of **8** bound to KIT-wt. **e, f** 2D pharmacophore model of **9** bound to KIT-wt. **g, h** 2D pharmacophore model of **10** bound to KIT-wt. **i, l** 2D pharmacophore model of **11** bound to KIT-wt. **j, k** 2D pharmacophore model of **12** bound to KIT-wt. LigandScout (v. 4.4.8) was used to generate the pharmacophore models and the corresponding 2D visualization.

Supp. Tab. 1 GR_{50} determinations on secondary or tertiary mutated-PDGFRA- and KIT cell lines of ligands 1-12. Data presented as mean values ± s.d; n ≥ 3, * n = 2., where n represents the number of independent experiments. n.d. not determined. T1-a cell lines: PDGFRA cell lines based on GIST-T1, generated by CRISPR/Cas9-mediated gene editing. GR_{50} half-maximal growth inhibition rate. Ava: avapritinib as reference inhibitor.

**Cpd**	**PDGFRA CTG GR_{50} [nM]**			**KIT CTG GR_{50} [nM]**	
	T1-a-T674I	T1-a-T674R	T1-a-5258	T1-T670I	T1-V654A
ava (1)	193 ± 107	918 ± 560	540 ± 30	457 ± 240	357 ± 142*
2	4224 ± 3911	3921 ± 2783	1340 ± 70	2867 ± 510	7556 ± 991
3	≥10000	n.d.	n.d.	n.d.	n.d.
4	8063 ± 2354	6779 ± 3777	≥10000	≥10000	≥10000
5	≥10000	≥10000	8260 ± 1700	≥10000	≥10000
6	≥10000	≥10000	9530 ± 650	≥10000	≥10000
7	9030 ± 1680	≥10000	9520 ± 810	≥10000	6730 ± 439
8	732 ± 367	880 ± 298	1660 ± 250	2560 ± 258	1171 ± 264
9	446 ± 99	1572 ± 1196	1330 ± 220	1495 ± 223	575 ± 159
10	193 ± 79	411 ± 193	2060 ± 430	386 ± 39	293 ± 83
11	256 ± 115	436 ± 249	3380 ± 360	413 ± 44	347 ± 108
12	364 ± 63	597 ± 235	2220 ± 350	695 ± 135	474 ± 155

Supp. Tab. 2 GR_{50} or EC_{50} determinations of non-KIT/PDGFRA driven control cell lines to assess off-target toxicity of ligands 1-13. GR_{50} half-maximal growth inhibition rate. EC_{50} half-maximal effective concentration. Data presented as mean values ± s.d; n = 3, where n represents the number of independent experiments. Ava: avapritinib as reference inhibitor.

Cpd	CTG GR_{50} [nM]	CTG EC_{50} [nM]	
	SK-LMS-1	ZR-75-1	MDA-MB-175VII
ava (1)	8423 ±2731	6558 ± 705	5778 ± 88
2	7713 ± 3929	6484 ± 1470	9191 ± 1401
3	≥10000	≥10000	≥10000
4	≥10000	≥10000	≥10000
5	≥10000	≥10000	≥10000
6	≥10000	≥10000	≥10000
7	≥10000	≥10000	≥10000
8	8631 ± 2371	≥10000	≥10000
9	9041 ± 1661	≥10000	≥10000
10	8787 ± 2101	≥10000	≥10000
11	6459 ± 3067	≥10000	≥10000
12	7334 ± 2310	≥10000	≥10000
13	9498 ± 869	≥10000	≥10000

Supp. Tab. 3 Predicted physicochemical parameters of the inhibitors 1-13 as well as calculated CNS-MPO Scores and measured MDCKII-MDR1 data for 1, 10-13. [a]Calculated with MarvinSketch software from Chemaxon.

Cpd	Physicochemical Parameters[a]						CNS-MPO Score[a]	MDCKII-MDR1 (P_{app} A→B) at 10 µM
	**clogP**	**clogD**	**MW**	**TPSA**	**HBD**	**pk$_a$**		
**avapritinib**	3.26	2.12	498.57	106.29	2	8.52	3.52	12.5
2	1.70	1.70	233.66	48.01	0	1.80	6.00	n.d.
3	1.04	-0.31	283.34	63.28	1	8.73	5.38	n.d.
4	2.07	2.07	361.41	80.27	0	3.22	5.96	n.d.
5	4.16	4.16	451.54	80.27	0	2.87	3.77	n.d.
6	3.84	3.84	463.55	80.27	0	2.88	3.92	n.d.
7	4.42	4.42	491.60	80.27	0	2.88	3.35	n.d.
8	4.08	3.59	526.62	83.51	0	7.72	3.67	n.d.
9	4.05	3.25	526.62	91.30	1	8.13	3.46	n.d.
10	3.08	3.08	540.61	109.37	1	2.79	3.52	11.3
11	3.84	3.84	552.62	109.37	1	2.79	2.77	10.1
12	3.70	3.70	556.61	118.60	1	2.79	2.60	12.4
13	4.94	4.94	602.68	109.37	1	2.79	2.14	3.5

Supplementary Methods

Supp. Tab. 4 Primer sequences of site-directed mutagenesis (mut.) for generation of the gatekeeper mutant crystallization constructs of KIT-T670I and PDGFRA-T674I as well as sequencing primers (seq.) of the used constructs.

Protein	Usage	Primer	Sequence 5'-3'
KIT	seq.	Forward	GTATTGTACGGCCGCATAATCG
	seq.	Reverse	GCTAGTTATTGCTCAGGGG
PDGFRA	seq.	Forward	GTTTCAGTTGCAAGTTGACACTGG
	seq.	Reverse	TACATGATGAAAGGAGGGAAGGGAG
KIT-T670I	mut.	Forward	GGTCCGACACTGGTTATTATTGAATATTGTTGTTATGGCG
	mut.	Reverse	CGCCATAACAACAATATTCAATAATAACCAGTGTCGGACC
PDGFRA-T674I	mut.	Forward	GTCCTATCTACATCATCATTGAGTACTGCTTCTACGG
	mut.	Reverse	CCGTAGAAGCAGTACTCAATGATGATGTAGATAGGAC

Supp. Tab. 5 Summary of crystallization conditions, proteins and corresponding PDB-IDs of the obtained complex crystal structures.

Structure	PDB-ID	Crystallization conditions
KIT-wt + **1**	8PQ9	6.6 mg/mL, 10% PEG8000, 20% Ethylen glycole, 30 mM NaI, 30 mM NaF, 30 mM NaBr, 100 mM Bicine, pH 8.5, 285.15 K
KIT-wt + **4**	8PQA	3 mg/mL, 23% PEG3350, 150 mM Na2-tartrate, 291.15 K
KIT-wt + **8**	8PQB	6.6 mg/mL, 1 M Na3-citrate, 100 mM Hepes, pH 6.5, 285.15 K
KIT-wt + **9**	8PQC	6.6 mg/mL, 750 mM Na3-citrate, 100 mM Hepes, pH 6.5, 293.15 K
KIT-wt + **10**	8PQD	6 mg/mL, 10% PEG6000, 200 mM LiCl, 100 mM Tris, pH 8.0, cryo-protected with 20% PEG400 in reservoir directly added to crystallization drop, 285.15 K
KIT-wt + **11**	8PQE	7.5 mg/mL, 12% PEG8000 20% Ethylen glycole, 30 mM NaI, 30 mM NaF, 30 mM NaBr, 100 mM Tris-Bicine, pH 9.5, 285.15 K
KIT-wt + **12**	8PQF	3 mg/mL, 21% PEG3350, 125 mM Li3-citrate, 285.15 K
KIT-T670I + **1**	8PQG	6 mg/mL, 25% PEG3350, 200 mM Na-citrate, 100 mM Bis-Tris-Propane, pH 6.5, 293.15 K

Structure Determination and Refinement. The structures of KIT WT in complex with compounds **1**, **4**, **8**, **9**, **10**, **11** and **12** were solved by molecular replacement with PHASER[1] using structure PDB-ID: 6GQK as a search model. Molecules in the asymmetric unit were manually adjusted using COOT[2] and the structure was refined with Phenix.refine 1.20.1_ 4487.[3] Inhibitor topology files were generated using eLBOW of the Phenix 1.14-3260 program package or the GRADE online server. [4] The refined structures were validated with the PDB validation server. PyMol (W.L. DeLano, The PyMOL Molecular Graphics System) was used for generating figures.

Supp. Tab. 6 Data statistic table of complex crystal structures of KIT-wt bound to 1 (PDB-ID:8PQ9) and 4 (PDB-ID: 8PQA) and KIT-T670I bound to 1 (PDB-ID:8PQG).

	KIT-WT + 1 (PDB-ID: 8PQ9)	KIT-T670I + 1 (PDB-ID: 8PQG)	KIT-WT + 4 (PDB-ID: 8PQA)
**Data collection**			
Space group	P 2_1 2_1 2_1 (19)	P 2_1 2_1 2_1 (19)	P 2_1 2_1 2_1 (19)
Cell dimensions			
a, b, c [Å]	59.02,59.30,191.76	58.75,59.09,192.42	58.76,58.88,193.41
α, β, γ [°]	90, 90, 90	90,90,90	90,90,90
Resolution [Å]	47.94-1.70 (1.80-1.70)	48.11-2.40 (2.50-2.40)	43.48-1.65 (1.70-1.65)
R_{meas} [%]	5.7 (139.1)	10 (170.9)	6.1 (260.6)
I / σI	22.42 (1.89)	14.36 (1.21)	19.49 (0.88)
Completeness [%]	100 (99.9)	99.8 (99.5)	100.0 (99.8)
$CC_{1/2}$	99.9 (89.5)	99.9 (55.7)	100.0 (77.9)
Redundancy	13.37 (13.43)	10.92 (8.27)	13.38 (13.68)
**Refinement**			
Resolution [Å]	35.00-1.70 (1.72-1.70)	48.10-2.40 (2.49-2.40)	38.21-1.65 (1.67-1.65)
No. reflections	74920	27015	81530
R_{work} / R_{free}	0.1905/0.2044 (0.5068/0.4994)	0.2061/0.2311 (0.3354/0.3866)	0.1877/0.2140 (0.5886/0.5600)
No. atoms			
Protein	4667 (Chain A: 2327, Chain C: 2340)	4614 (Chain A: 2274, Chain C: 2340)	4696 (Chain A: 2323, Chain C: 2373)
Ligands	79 (Chain B: 37, Chain D: 37, Chain F: 5)	75 (Chain D:37, Chain E:37)	54 (Chain B: 27, Chain E: 27)
Water	486	42	475
B-factors (Å^2)			
Protein	40.88 (Chain A:41.84, Chain C: 41.11)	67.71 (Chain A: 69.56, Chain C: 67.35)	44.79 (Chain A: 47.06, Chain C:43.56)
Ligand	38.21 (Chain B: 39.56, Chain D: 36.05, Chain F: 44.24)	61. 53 (Chain D: 65.34, Chain E: 57.72)	42.48 (Chain B: 46.54, Chain E: 38.42)
Water	47.88	64.18	48.09
RMS deviations			
Bond	0.006	0.004	0.010
Lengths [Å]	0.873	0.586	0.986
Bond angles [°]			
Ramachandran [%]			
Outliers	0	0	0
Allowed	1.36	2.59	2.23
Favored	98.64	97.41	97.77

Supp. Tab. 7 Data statistic table of complex crystal structures of KIT-wt bound to 8 (PDB-ID:8PQB), 9 (PDB-ID: 8PQC) and 10 (PDB-ID:8PQD).

	KIT-WT + 8 (PDB-ID: 8PQB)	KIT-WT + 9 (PDB-ID: 8PQC)	KIT-WT + 10 (PDB-ID: 8PQD)
**Data collection**			
Space group	P 3_1 2 1 (152)	P 2_1 2_1 2_1 (19)	P 2_1 2_1 2_1 (19)
Cell dimensions			
a, b, c [Å]	55.67,55.67,186.08	59.22,59.22,192.60	59.39,59.37,192.89
α, β, γ [°]	90,90,120	90,90,90	90,90,90
Resolution [Å]	48.21-1.87 (1.90-1.87)	48.15-1.77 (1.80-1.77)	48.22-1.50 (1.60-1.50)
R_{meas} [%]	7.8 (156.0)	12.9 (169.1)	7.7 (126.5)
I / σI	21.19 (2.03)	17.03 (1.48)	16.16 (2.02)
Completeness [%]	100.0 (100.0)	100.0 (99.5)	99.0 (98.6)
$CC_{1/2}$	99.9 (75.3)	99.9 (61.3)	99.9 (84.5)
Redundancy	18.15 (18.48)	11.60 (6.72)	13.90 (14.36)
**Refinement**			
Resolution [Å]	46.67-1.87 (1.94-1.87)	43.53-1.77 (1.80-1.77)	41.99-1.50 (1.52-1.50)
No. reflections	28621	67004	108872
R_{work} / R_{free}	0.1980/0.2339 (0.3819-0.3964)	0.1814/0.2063 (0.3910-0.4003)	0.1746/0.1943 (0.2941/0.3373)
No. atoms			
Protein	2217	4727 (Chain A: 2373, Chain B: 2354)	4762 (Chain A: 2369, Chain B:2393)
Ligand	39	78 (Chain D: 39, Chain E: 39)	84 (Chain D: 44, Chain E:40)
Water	154	371	602
B-factors (Å^2)			
Protein	41.24	33.78 (Chain A: 34.35, Chain B: 34.20)	33.84 (Chain A: 33.92, Chain B: 34.76)
Ligand	36.83	29. 43 (Chain D: 28.35, Chain E: 30.50)	28.77 (Chain D: 28.5, Chain E:29.08)
Water	42.87	37.11	41.73
RMS deviations			
Bond Lengths [Å]	0.005	0.008	0.008
Bond angles [°]	0.843	0.944	0.864
Ramachandran [%]			
Outliers	0	0	0
Allowed	1.10	1.72	1.87
Favored	98.90	98.28	98.13

Supp. Tab. 8 Data statistic table of complex crystal structures of KIT-wt bound to 11 (PDB-ID:8PQE) and 12 (PDB-ID: 8PQF).

	KIT-WT + 11 (PDB-ID: 8PQE)	KIT-WT + 12 (PDB-ID: 8PQF)
**Data collection**		
Space group	P 2_1 2_1 2_1 (19)	P 2_1 2_1 2_1 (19)
Cell dimensions		
a, b, c [Å]	58.97,62.52,191.60	58.09,59.25,191.83
α, β, γ [°]	90,90,90	90,90,90
Resolution [Å]	44.69-2.00 (2.10-2.00)	47.96-1.90 (2.00-1.90)
R_{meas} [%]	6.7 (225.0)	9.4 (159.7)
I / σI	19.57 (1.22)	14.58 (1.53)
Completeness [%]	100.0 (100.0)	100.0 (100.0)
$CC_{1/2}$	100.0 (70.1)	99.9 (73.8)
Redundancy	13.28 (13.98)	13.36 (12.86)
**Refinement**		
Resolution [Å]	44.69-2.00 (2.04-2.00)	43.00-1.90 (1.93-1.90)
No. reflections	48818	53176
R_{work} / R_{free}	0.2135/0.2556 (0.4123/0.4374)	0.1957/0.2186 (0.3447/0.3412)
No. atoms		
Protein	4382 (Chain A: 2173, Chain B: 2209)	4667 (Chain A: 2329, Chain C: 2338)
Ligand	86 (Chain D: 41, Chain E: 41, Chain A: 4)	82 (Chain B: 41, Chain E: 41)
Ions	2 (Chain G:1, Chain F: 1)	-
Water	137	284
B-factors (Å^2)		
Protein	62.57 (Chain A: 61.02, Chain B: 64.99)	47.6 (Chain A: 49.77, Chain C: 46.35)
Ligand	63.84 (Chain D: 64.82, Chain E: 61.77, Chain A: 74.89)	45.61 (Chain B: 47.89, Chain E: 43.32)
Ions	59.83 (Chain G: 72.37, Chain F: 47.29)	-
Water	60.95	49.33
RMS deviations		
Bond Lengths [Å]	0.004	0.003
Bond angles [°]	1.015	0.634
Ramachandran [%]		
Outliers	0	0
Allowed	0.73	1.56
Favored	99.27	98.44

Supp. Tab. 9 Summary of crystallization conditions, proteins and corresponding PDB-IDs of the obtained complex crystal structures.

**Structure**	**PDB-ID**	**Crystallization conditions**
PDGFRA-T674I + **1**	8PQH	10 mg/mL, 13% PEG3350, 200 mM KCl, 293.15 K
PDGFRA-T674I + **9**	8PQI	5 mg/mL, 5% PEG3350, 200 mM KCl, 293.15 K
PDGFRA-wt	8PQJ	10 mg/mL, 15% PEG3350, 100 mM Bis-Tris-Propane, pH 7.5, 285.15 K
PDGFRA-T674I	8PQK	10 mg/mL, 22% PEG3350, 100 mM Bis-Tris-Propane, pH 7.5, 293.15 K

Structure Determination and Refinement. The structures of PDGFRA apo or in complex with ligands **1** and **9** were solved by molecular replacement with PHASER[1] using structure PDB-ID: 5GRN as a search model. Molecules in the asymmetric unit were manually adjusted using COOT[2] and the structure was refined with Phenix.refine 1.20.1_4487. [3] Inhibitor topology files were generated using eLBOW of the Phenix 1.14-3260 program package or the GRADE online server [4]. The refined structure was validated with the PDB validation server. PyMol (W.L. DeLano, *The PyMOL Molecular Graphics System*) was used for generating figures.

Supp. Tab. 10 Data statistic table of complex crystal structures of apo PDGFRA-wt (PDB-ID:8PQJ) and apo PDGFRA-T674I (PDB-ID: 8PQK).

	PDGFRA-wt Apo (PDB-ID: 8PQJ)	PDGFRA-T674I Apo (PDB-ID: 8PQK)
**Data collection**		
Space group	C 1 2 1 (5)	C 1 2 1 (5)
Cell dimensions		
a, b, c [Å]	96.38,49.18,77.04	95.90,49,02,76.98
α, β, γ [°]	90,100.78,90	90,100.70,90
Resolution [Å]	47.34-1.82 (1.90-1.82)	47.12-2.00 (2.10-2.00)
R_{meas} [%]	8.7 (171.3)	12.9 (85.1)
$I / \sigma I$	12.45 (1.21)	9.24 (2.04)
Completeness [%]	99.9 (100.0)	99.3 (98.1)
$CC_{1/2}$	99.9 (46.1)	99.7 (78.2)
Redundancy	6.84 (6.52)	6.82 (6.69)
**Refinement**		
Resolution [Å]	37.84-1.82 (1.88-1.82)	43.81-2.00(2.08-2.00)
No. reflections	31998	23797
R_{work} / R_{free}	0.1838/0.2151 (0.4402/0.4581)	0.1960/0.2219 (0.3095/0.3573)
No. atoms		
Protein	2632 (Chain A: 2632)	2579 (Chain A: 2579)
Ligand	-	-
Water	204	138
B-factors (Å^2)		
Protein	40.20 (Chain A: 40.20)	37.36 (Chain A: 37.36)
Ligand	-	-
Water	44.63	41.17
RMS deviations		
Bond Lengths [Å]	0.005	0.002
Bond angles [°]	0.776	0.473
Ramachandran [%]		
Outliers	0	0
Allowed	0.93	1.27
Favored	99.07	98.73

Supp. Tab. 11 Data statistic table of complex crystal structures of PDGFRA-T674I bound to 1 (PDB-ID:8PQH) and 9 (PDB-ID: 8PQI).

	PDGFRA-T674I + 1 (PDB-ID: 8PQH)	PDGFRA-T674I + 9 (PDB-ID: 8PQI)
**Data collection**		
Space group	P 2_1 2_1 2_1 (19)	P 2_1 2_1 2_1 (19)
Cell dimensions		
a, b, c [Å]	52.27,73.26,102.59	52.39, 74.04, 102.26
α, β, γ [°]	90,100.69,90	90.00, 90.00, 90.00
Resolution [Å]	46.57-2.50 (2.60-2.50)	46.63-2.60 (2.70-2.6)
R_{meas} [%]	8.0 (149.9)	11.8 (160.7)
I / σ*I*	18.79 (2.06)	12.39 (1.80)
Completeness [%]	100.0 (100.0)	98.3 (99.9)
$CC_{1/2}$	99.9 (77.0)	99.8 (87.6)
Redundancy	13.09 (13.67)	13.05 (13.54)
**Refinement**		
Resolution [Å]	46.57-2.5 (2.60-2.50)	46.63-2.60 (2.86-2.60)
No. reflections	14174	12197
R_{work} / R_{free}	0.2184/0.2486 (0.3543/0.3967)	0.2320/0.2540 (0.3184/0.3117)
No. atoms		
Protein	2430 (Chain A: 2430)	2352 (Chain A: 2352)
Ligand	44 (Chain B:37, Chain D: 7)	46 (Chain D: 39, Chain C: 7)
Water	23	8
B-factors		
Protein	80.52 (Chain A: 80.52)	79.69 (Chain A: 79.69)
Ligand	74.59 (Chain B: 72.65, Chain D: 84.89)	76.75 (Chain D: 73.29, Chain C: 96.07)
Water	74.69	69.97
RMS deviations		
Bond Lengths [Å]	0.009	0.002
Bond angles [°]	1.004	0.594
Ramachandran [%]		
Outliers	0	0
Allowed	2.54	2.39
Favored	97.46	97.61

Supp. Tab. 12 Genetic description and origin of used sarcoma cell lines.

Cell line	Genotype	Establishment
GIST-T1	Primary activating mutation in KIT exon 11 (RRID: CVCL_4976, V560_Y578del)	Established by Takahiro Taguchi (Kochi University, Kochi, Japan)
T1-D816E	Primary activating mutation in KIT exon 11, secondary mutation D816E (RRID: CVCL_A9N0)	Long-term IM treatment of GIST-T1, Brian Rubin (Cleveland Clinic, OH)
T1-T670I	Primary activating mutation in KIT exon 11, secondary mutation T670I (RRID: CVCL_A9M9)	Long-term IM treatment of GIST-T1, Brian Rubin (Cleveland Clinic, OH)
T1-V654A	Primary activating mutation in KIT exon 11, secondary mutation V654A	CRISPR/Cas9-mediated gene editing, Thomas Mühlenberg (WTZ Essen, Germany)
T1-a-D842V	Endogenous PDGFRA primary mutation D842V	CRISPR/Cas9-mediated gene editing, Thomas Mühlenberg (WTZ Essen, Germany)
T1-a-G680R	Endogenous PDGFRA primary mutation D842V, secondary mutation G680R	CRISPR/Cas9-mediated gene editing, Thomas Mühlenberg (WTZ Essen, Germany)
T1-a-T674I	Endogenous PDGFRA primary mutation D842V, secondary mutation T674I	CRISPR/Cas9-mediated gene editing, Thomas Mühlenberg (WTZ Essen, Germany)
T1-a-T674R	Endogenous PDGFRA primary mutation D842V, secondary mutation T674R	CRISPR/Cas9-mediated gene editing, Thomas Mühlenberg (WTZ Essen, Germany)
T1-a-5258	Triple mutated cell line, endogenous PDGFRA primary mutation D842V, G652E, V658A	CRISPR/Cas9-mediated gene editing, Thomas Mühlenberg (WTZ Essen, Germany)
GIST-48B	KIT/PDGFRA independent cell line (RRID: CVCL_M441)	Established by Jonathan Fletcher (Brigham and Women’s Hospital, Boston, MA)
SK-LMS-1	Non-KIT non kinase driven leiomyosarcoma cell line (RRID: CVCL_0628)	Established by Jonathan Fletcher (Brigham and Women’s Hospital, Boston, MA)

Sequences of sgRNAs and ssDNA homology directed repair templates used for the generation of isogenic sublines have been previously published (Mühlenberg, MCT 2019; Grunewald CD, 2021).[5, 6]

Supp. Tab. 13 References used for MDCKII-MDR1 assays.

Reference	MDCKII-MDR1 (P_{app}A→B) [10^{-6} cm/s]	MDCKII-MDR1 (P_{app}B→A) [10^{-6} cm/s]	MDCKII-MDR1 (Ratio B→A: A→B) [NA]	Comment
Propanolol	32 ± 10.3 (134 runs)	not routinely determined	NA	permeable
Atenolol	1.1 ± 3.5 (130 assay runs)	not routinely determined	NA	non-permeable
Digoxin	1.9 ± 4.5 (127 assay runs)	15.4 ± 16.0 (128 data points)	Has to be >3	efflux

Synthetic Procedures. Safety statement: No unexpected or unusually high safety hazards have been encountered. The reagents and solvents used were commercially purchased from various suppliers such as Activate Scientific, Alfa Aesar, Apollo Scientific, BLDpharm, Merck, Sigma-Aldrich, TCI Chemicals, or VWR and were used as received without further purification. Air- and/or humidity-sensitive reactions were performed in heated glassware and under inert gas, using either argon or nitrogen atmospheres. Reactions were monitored by thin-layer chromatography (TLC) and liquid chromatography-mass spectrometry (LC)-MS. Analytical TLC was performed on Merck 60 F254 aluminum-backed silica gel plates and visualized under UV light (λ = 254 and 366 nm) and via staining solutions. The LC-MS analysis was recorded via the HPLC system LCQ Advantage Max of the 1200 series from Agilent with Eclipse XDB-C18-column (5 µM 150 × 1.6 mm, Phenomenex). The purification of the compounds by column chromatography was performed either manually on VWR silica gel (40-63 µm particle size), on the Isolera One™ Flash System from Biotage, or on the Reveleris PREP HPLC from Büchi using Büchi Reveleris or Büchi FlashPure EcoFlex C18 columns. High-resolution electrospray ionization mass spectra (ESI-Fourier transform mass spectrometry) were recorded on a Thermo LTQ Orbitrap (high-resolution mass spectrometer from Thermo Electron) coupled to an Accela HPLC system supplied with a Hypersil GOLD column (Thermo Electron). Preparative HPLC was conducted on a Büchi Reveleris Prep System with a VP 250/21 Nucleodur C18 column from Macherey-Nagel and monitored by UV at λ = 210, 254, and 280 nm. The ^{1}H and ^{13}C nuclear magnetic resonance (NMR) spectra were recorded on either a Bruker AVANCE III HD 400 MHz, Bruker AVANCE III HD 500 MHz, Agilent DD2 500 MHz, Bruker AVANCE III HD 600 MHz or Bruker AVANCE III HD 700 MHz. Chemical shifts (δ) are reported in parts per million (ppm) and referenced to the solvent signal DMSO-d6 (2.50 or 39.52 ppm) or $CDCl_3$ (7.26 or 77.16 ppm, respectively). The spin multiplicity of the ^{1}H NMR spectra is represented in an established manner: (s) singlet, (d) doublet, (t) triplet, and (m) multiplet. All final compounds were purified to > 95 % purity as determined by high-performance liquid chromatography (HPLC).

*Common Procedure A: Nucleophilic aromatic substitution with 4-chloro-6-(1-methyl-1H-pyrazol-4-yl)pyrrolo[2,1-f][1,2,4]triazine (**2**)*

TEA (3.0 eq.) was added to a stirred solution of the respective amine (1.0 eq.) and 4-chloro-6-(1-methyl-1H-pyrazol-4-yl)pyrrolo[2,1-f][1,2,4]triazine (1.0 eq.) in dry THF. The reaction mixture was allowed to stir for 6 h at room temperature or 4 h at 40 °C. Afterwards the suspension was diluted with water and extracted with DCM three times. The combined organic fractions were dried over Na_2SO_4, filtered, and concentrated *in vacuo.* The crude product was absorbed onto silica gel and purified by flash column chromatography (DCM/MeOH) to yield the desired product.

Common Procedure B: Acid mediated carbamate cleavage

The respective BOC protected piperazines derivatives were dissolved in DCM and TFA (3:1). The resulting mixture was allowed to stir for 12 h at room temperature. After the completion of the reaction was monitored via TLC and LC-MS, the suspension was basified with 10 M NaOH solution and subsequently extracted with DCM three times. The combined organic fractions were dried over Na_2SO_4, filtered, and concentrated *in vacuo.* The crude product was absorbed onto silica gel and purified by flash column chromatography (DCM/MeOH + 1 % NH_3) to yield the desired product.

Common Procedure C: Nucleophilic aromatic substitution with 5-bromo-2-chloropyrimidine
TEA (3.0 eq.) was added to a stirred solution of the respective piperazine derivate (1.0 eq.) dissolved in EtOH, followed by the addition of the 5-bromo-2-chloropyrimidine (1.0 eq.). The resulting reaction mixture was stirred for 16 h at 60 °C. After the completion of the reaction was monitored via TLC and LC-MS, the suspension was diluted with water and extracted with DCM three times. The combined organic fractions were dried over Na_2SO_4, filtered, and concentrated *in vacuo*. The crude product was absorbed onto silica gel and purified by flash column chromatography (DCM/MeOH) to yield the desired product.

Common Procedure D: Suzuki cross coupling with 2-benzyl-4,4,5,5-tetramethyl-1,3,2-dioxaborolane
The respective bromopyrimidine derivative (1.0 eq.) and 2-benzyl-4,4,5,5-tetramethyl-1,3,2-dioxaborolane (1.5 eq.) were dissolved in 1,4-dioxane and water (0.1 M; 5:1) under an argon atmosphere. The mixture was degassed for 15 min and then 3.0 eq. of the base Cs_2CO_3 as well as 0.15 eq. of [1,1'-Bis(diphenylphosphino)ferrocene]palladium(II)dichloride were added subsequently. Afterwards the suspension was stirred for 1 h at 120 °C in a microwave. The cooled reaction mixture was filtered over Celite and extracted with DCM and water for five times. The combined organic fractions were dried over Na_2SO_4 and concentrated *in vacuo*. The crude product was absorbed onto silica gel and purified by flash column chromatography (DCM/MeOH) to yield the desired product.

*4-chloro-6-(1-methyl-1H-pyrazol-4-yl)pyrrolo[2,1-f][1,2,4]triazine (**2**) was purchased from BLDpharm.* **^{1}H NMR** (600 MHz, DMSO-d$_6$) δ ppm 3.88 (s, 3 H) 7.27 (s, 1 H) 7.95 (s, 1 H) 8.20 (s, 1 H) 8.38 (s, 1 H) 8.55 (s, 1 H). **^{13}C NMR** (151 MHz, DMSO-*d*$_6$) δ ppm 39.17 (s, 1 C) 101.24 (s, 1 C) 115.18 (s, 1 C) 118.66 (s, 1 C) 121.95 (s, 1 C) 123.42 (s, 1 C) 129.10 (s, 1 C) 137.49 (s, 1 C) 145.70 (s, 1 C) 152.38 (s, 1 C). **HPLC-MS (ESI):** [Rt]: 4.96 min, *m/z* for $C_{10}H_8ClN_5$ ([M+H+]), 233.05 cacld., 234.12, found.

*Synthesis of 2-(piperazin-1-yl)pyrimidine--6-(1-methyl-1H-pyrazol-4-yl)-4-(4-(pyrimidin-2-yl)piperazin-1-yl)pyrrolo[2,1-f][1,2,4]triazine (**4**).*
4-chloro-6-(1-methyl-1H-pyrazol-4-yl)pyrrolo[2,1-f][1,2,4]triazine (**2**, 10.0 mg, 0.04 mmol, 1.0 eq.), 2-(piperazin-1-yl)pyrimidine (6.1 µL, 0.04 mmol, 1.0 eq.), TEA (6.2 µL, 0.04 mmol, 1.0 eq.) were used following common procedure A (6 h at room temperature) and yielded the final compound as a white solid (11.8 mg, 76 %).**^{1}H NMR** (600 MHz, DMSO-*d*$_6$) δ ppm 3.86 (s, 3 H) 3.95 (dd, *J*=6.24, 4.40 Hz, 4 H) 4.12 (dd, *J*=6.24, 4.40 Hz, 4 H) 6.69 (t, *J*=4.77 Hz, 1 H) 7.23 (d, *J*=1.83 Hz, 1 H) 7.82 (d, *J*=0.73 Hz, 1 H) 7.88 (s, 1 H) 7.99 (d, *J*=1.83 Hz, 1 H) 8.42 (d, *J*=4.40 Hz, 2 H). **^{13}C NMR** (151 MHz, DMSO-*d*$_6$) δ ppm 39.05 (s, 1 C) 43.32 (s, 4 C) 102.16 (s, 1 C) 110.88 (s, 1 C) 114.98 (s, 1 C) 115.94 (s, 1 C) 116.27 (s, 1 C) 118.31 (s, 1 C) 128.03 (s, 1 C) 136.87 (s, 1 C) 146.97 (s, 1 C) 154.18 (s, 1 C) 158.49 (s, 2 C) 161.51 (s, 1 C). **HPLC-MS (ESI):** [Rt]: 4.66 min, *m/z* for $C_{18}H_{19}N_9$ ([M+H+]), 361.18 cacld., 362.29, found.

*Synthesis of tert-butyl 4-(6-(1-methyl-1H-pyrazol-4-yl)pyrrolo[2,1-f][1,2,4]triazin-4-yl)piperazine-1-carboxylate (**S1**).*
4-chloro-6-(1-methyl-1H-pyrazol-4-yl)pyrrolo[2,1-f][1,2,4]triazine (**2**, 250.0 mg, 1.10 mmol, 1.0 eq.), tert-butyl piperazine-1-carboxylate (219.2 mg, 1.20 mmol, 1.1 eq.), TEA (0.1 mL, 1.1 mmol, 1.0 eq.) were used following common procedure A (3 h at 40 °C) and yielded the final compound as a pale yellow solid (372.1 mg, 91 %).**^{1}H NMR** (600 MHz, DMSO-d$_6$) δ ppm 1.45 (s, 9 H) 3.53 (m, 4 H) 3.86 (s, 3 H) 3.98 - 4.01 (m, 4 H) 7.20 (d, J=1.59 Hz, 1 H) 7.81 (s, 1 H) 7.87 (s, 1 H) 7.98 (d, J=1.54 Hz, 1 H) 8.03 (s, 1 H). **^{13}C NMR** (151 MHz, DMSO-d$_6$) d ppm 28.55 (s, 3 C) 39.04 (s, 1 C) 45.33 (s, 4 C) 79.66 (s, 1 C) 102.10 (s, 1 C) 114.89 (s, 1 C)

115.93 (s, 1 C) 116.25 (s, 1 C) 118.35 (s, 1 C) 128.01 (s, 1 C) 136.85 (s, 1 C) 146.90 (s, 1 C) 154.12 (s, 1 C) 154.34 (s, 1 C). **HPLC-MS (ESI):** [Rt]: 5.71 min, m/z for $C_{19}H_{25}N_7O_2$ ([M+H+]), 383.21 cacld., 384.03, found.

*Synthesis of tert-butyl 6-(6-(1-methyl-1H-pyrazol-4-yl)pyrrolo[2,1-f][1,2,4]triazin-4-yl)-2,6-diazaspiro[3.3]heptane-2-carboxylate (**S2**).*
4-chloro-6-(1-methyl-1H-pyrazol-4-yl)pyrrolo[2,1-f][1,2,4]triazine (**2**, 30.0 mg, 0.1 mmol, 1.0 eq.), tert-butyl 2,6-diazaspiro[3.3]heptane-2-carboxylate oxalate (37.0 mg, 0.1 mmol, 1.0 eq.), TEA (1.8 µL, 0.1 mmol, 1.0 eq.) were used following common procedure A (6 h at room temperature) and yielded the final compound as a white solid (33.4 mg, 66 %).**^{1}H NMR** (600 MHz, CHLOROFORM-d) δ ppm 1.46 (s, 9 H) 3.95 (s, 4 H) 4.19 (s, 4 H) 4.69 (s., 3 H) 6.64 (br. s., 1 H) 7.55 (s, 1 H) 7.68 (s, 2 H) 7.86 (s, 1 H). **^{13}C NMR** (151 MHz, CHLOROFORM-d) δ ppm 28.54 (s, 3 C) 34.25 (s, 1 C) 39.32 (s, 1 C) 59.66 (s, 2 C) 68.67 (s, 2 C) 80.44 (s, 1 C) 116.15 (s, 1 C) 125.03 (s, 1 C) 127.10 (s, 1 C) 127.82 (s, 1 C) 128.38 (s, 1 C) 137.19 (s, 1 C) 137.62 (s, 1 C) 138.16 (s, 1 C) 151.68 (s, 1 C) 156.06 (s, 1 C). **HPLC-MS (ESI):** [Rt]: 4.46 min, m/z for $C_{20}H_{25}N_7O_2$ ([M+H+]), 395.21 cacld., 396.18 found.

*Synthesis of tert-butyl 7-(6-(1-methyl-1H-pyrazol-4-yl)pyrrolo[2,1-f][1,2,4]triazin-4-yl)-2,7-diazaspiro[3.5]nonane-2-carboxylate (**S3**).*
4-chloro-6-(1-methyl-1H-pyrazol-4-yl)pyrrolo[2,1-f][1,2,4]triazine (**2**, 27.9 mg, 0.1 mmol, 0.9 eq.), tert-butyl 2,7-diazaspiro[3.5]nonane-2-carboxylate (30.0 mg, 0.1 mmol, 1.0 eq.), TEA (1.9 µL, 0.1 mmol, 1.0 eq.) were used following common procedure A (6 h at room temperature) and yielded the final compound as a white solid (54.9 mg, 98 %).**^{1}H NMR** (400 MHz, DMSO-d_6) δ ppm 1.32 (s, 9 H) 1.73 (t, J=5.38 Hz, 4 H) 3.56 (br. s., 4 H) 3.77 (s, 3 H) 3.83 (br. s., 4 H) 7.05 (d, J=1.47 Hz, 1 H) 7.74 (s, 1 H) 7.76 (s, 1 H) 7.87 (d, J=1.47 Hz, 1 H) 7.96 (s, 1 H). **^{13}C NMR** (101 MHz, DMSO-d_6) d ppm 28.09 (s, 3 C) 33.47 (s, 2 C) 34.90 (s, 1 C) 38.54 (s, 1 C) 42.83 (s, 4 C) 78.47 (s, 1 C) 101.27 (s, 1 C) 114.41 (s, 1 C) 115.38 (s, 1 C) 115.72 (s, 1 C) 117.71 (s, 1 C) 127.58 (s, 1 C) 136.39 (s, 1 C) 146.49 (s, 1 C) 153.36 (s, 1 C) 155.69 (s, 1 C). **HPLC-MS (ESI):** [Rt]: 5.79 min, m/z for $C_{22}H_{29}N_7O_2$ ([M+H+]), 423.24 cacld., 424.24 found.

*Synthesis of 6-(1-methyl-1H-pyrazol-4-yl)-4-(piperazin-1-yl)pyrrolo[2,1-f][1,2,4]triazine (**3**).*
tert-butyl 4-(6-(1-methyl-1H-pyrazol-4-yl)pyrrolo[2,1-f][1,2,4]triazin-4-yl)piperazine-1-carboxylate (**S1**, 350.0 mg, 0.9 mmol, 1.0 eq.) was used following common procedure B and yielded the final compound as a white solid (166.7 mg, 64 %). **^{1}H NMR** (600 MHz, DMSO-d_6) δ ppm 2.82 - 2.86 (m, 4 H) 3.85 (s, 3 H) 3.89 - 3.93 (m, 4 H) 7.14 (s, 1 H) 7.81 (s, 1 H) 7.84 (s, 1 H) 7.95 (s, 1 H) 8.03 (s, 1 H). **^{13}C NMR** (151 MHz, DMSO-d_6) δ ppm 39.02 (s, 1 C) 46.13 (s, 2 C) 47.07 (s, 2 C) 101.92 (s, 1 C) 114.94 (s, 1 C) 115.79 (s, 1 C) 116.26 (s, 1 C) 118.13 (s, 1 C) 128.02 (s, 1 C) 136.85 (s, 1 C) 146.96 (s, 1 C) 153.98 (s, 1 C). **HPLC-MS (ESI):** [Rt]: 2.41 min, m/z for $C_{14}H_{17}N_7$ ([M+H+]), 283.15 cacld., 284.22 found.

*Synthesis of 6-(1-methyl-1H-pyrazol-4-yl)-4-(2,6-diazaspiro[3.3]heptan-2-yl)pyrrolo[2,1-f][1,2,4]triazine (**S4**).*
7-(6-(1-methyl-1H-pyrazol-4-yl)pyrrolo[2,1-f][1,2,4]triazin-4-yl)-2,7-diazaspiro[3.5]nonane-2-carboxylate (**S3**, 30.0 mg, 0.08 mmol, 1.0 eq.) was used following common procedure B and yielded the final compound as a white solid (14.2 mg, 63 %).**^{1}H NMR** (600 MHz, DMSO-d_6) δ ppm 1.99 (s. 1 H) 3.61 (br. s., 4 H) 3.75 (s., 3 H) 4.32-4.68 (br. s., 4 H) 6.85 (s, 1 H) 7.78 (d, J=2.20 Hz, 2 H) 7.90 (s, 1 H) 8.01 (s, 1 H). **^{13}C NMR** (151 MHz, DMSO-d_6) δ ppm 21.24 (s, 1 C) 39.04 (s, 1 C) 56.39 (s, 2 C) 60.24 (s, 2 C) 99.52 (s, 1 C) 114.45 (s, 1 C) 115.24 (s, 1 C) 116.33 (s, 1 C) 118.54 (s, 1 C) 127.93 (s, 1 C) 136.78 (s, 1 C) 147.74 (s, 1 C) 153.70 (s, 1 C). **HPLC-MS (ESI):** [Rt]: 1.89 min, m/z for $C_{15}H_{17}N_7$ ([M+H+]), 295,15 cacld., 296.23 found.

*Synthesis of 6-(1-methyl-1H-pyrazol-4-yl)-4-(2,7-diazaspiro[3.5]nonan-7-yl)pyrrolo[2,1-f][1,2,4]triazine (**S5**).*

tert-butyl-6-(6-(1-methyl-1H-pyrazol-4-yl)pyrrolo[2,1-f][1,2,4]triazin-4-yl)-2,6-diazaspiro[3.3]heptane-2-carboxylate (**S2**, 45.0 mg, 0.11 mmol, 1.0 eq.) was used following common procedure B and yielded the final compound as a white solid (34.0 mg, quant.).

^{1}H NMR (600 MHz, METHANOL-d$_4$) δ ppm 1.90 (br. s., 1 H) 1.96 (br. s., 4 H) 3.62 (s, 4 H) 3.91 (s, 3 H) 3.96 (br. s., 4 H) 7.03 (s, 1 H) 7.76 (d, J=1.47 Hz, 1 H) 7.77 (d, J=1.83 Hz, 2 H) 7.89 (s, 1 H). **^{13}C NMR** (151 MHz, METHANOL-d$_4$) δ ppm 36.64 (s, 2 C) 39.02 (s, 1 C) 44.40 (s, 1 C) 46.54 (s, 2 C) 57.04 (s, 2 C) 103.08 (s, 1 C) 116.52 (s, 1 C) 116.95 (s, 1 C) 118.08 (s, 1 C) 119.43 (s, 1 C) 129.24 (s, 1 C) 137.89 (s, 1 C) 147.90 (s, 1 C) 155.51 (s, 1 C). **HPLC-MS (ESI):** [Rt]: 2.78 min, m/z for $C_{17}H_{21}N_7$ ([M+H+]), 323,19 cacld., 324.33 found.

*Synthesis of 4-(4-(5-bromopyrimidin-2-yl)piperazin-1-yl)-6-(1-methyl-1H-pyrazol-4-yl)pyrrolo[2,1-f][1,2,4]triazine (**S6**).*

6-(1-methyl-1H-pyrazol-4-yl)-4-(piperazin-1-yl)pyrrolo[2,1-f][1,2,4]triazine (**3**, 130.0 mg, 0.46 mmol, 1.0 eq.), 5-bromo-2-chloropyrimidine (88.8 mg, 0.46 mmol, 1.0 eq.) and TEA (63.6 µL, 0.46 mmol, 1.0 eq.) were used following common procedure C and yielded the final compound as a white solid (127.6 mg, 63 %.). **^{1}H NMR** (500 MHz, DMSO-d$_6$) δ ppm 3.79 (s, 3 H) 3.86 (dd, J=6.26, 4.43 Hz, 4 H) 4.06 (dd, J=6.33, 4.35 Hz, 4 H) 7.19 (d, J=1.37 Hz, 1 H) 7.75 (s, 1 H) 7.83 (s, 1 H) 7.94 (d, J=1.37 Hz, 1 H) 7.96 (s, 1 H) 8.46 (s, 2 H). **^{13}C NMR** (126 MHz, DMSO-d$_6$) δ ppm 39.06 (s, 1 C) 43.46 (s, 4 C) 106.26 (s, 1 C) 114.78 (s, 1 C) 116.15 (s, 1 C) 116.36 (s, 1 C) 118.52 (s, 1 C) 128.06 (s, 1 C) 130.95 (s, 1 C) 133.14 (s, 1 C) 136.87 (s, 1 C) 153.67 (s, 1 C) 158.54 (s, 2 C) 159.83 (s, 1 C). **HPLC-MS (ESI):** [Rt]: 6.25 min, m/z for $C_{18}H_{18}BrN_9$ ([M+H+]), 439,09 cacld., 440.32 found.

*Synthesis of 4-(6-(5-bromopyrimidin-2-yl)-2,6-diazaspiro[3.3]heptan-2-yl)-6-(1-methyl-1H-pyrazol-4-yl)pyrrolo[2,1-f][1,2,4]triazine (**S7**).*

6-(1-methyl-1H-pyrazol-4-yl)-4-(2,6-diazaspiro[3.3]heptan-2-yl)pyrrolo[2,1-f][1,2,4]triazine (**S5**, 10.0 mg, 0.03 mmol, 1.0 eq.), 5-bromo-2-chloropyrimidine (6.6 mg, 0.03 mmol, 1.0 eq.) and TEA (4.9 µL, 0.03 mmol, 1.0 eq.) were used following common procedure C and yielded the final compound as a white solid (13.1 mg, 85 %.). **^{1}H NMR** (600 MHz, DMSO-d6) δ ppm 4.29 (s, 4 H) 4.42 (br. s., 2 H) 4.81 (br. s., 2 H) 6.83 (d, J=1.83 Hz, 1 H) 7.77 (d, J=0.73 Hz, 1 H) 7.81 (s, 1 H) 7.92 (d, J=1.47 Hz, 1 H) 8.00 (s, 1 H) 8.48 (s, 2 H). **^{13}C NMR** (151 MHz, DMSO-d$_6$) δ ppm 34.76 (s, 1 C) 39.04 (s, 1 C) 60.53 (s, 4 C) 99.43 (s, 1 C) 106.96 (s, 1 C) 114.46 (s, 1 C) 115.31 (s, 1 C) 116.30 (s, 1 C) 118.58 (s, 1 C) 127.92 (s, 1 C) 136.76 (s, 1 C) 147.75 (s, 1 C) 153.74 (s, 1 C) 158.48 (s, 2 C) 161.11 (s, 1 C). **HPLC-MS (ESI):** [Rt]: 4.38 min, m/z for $C_{19}H_{18}BrN_9$ ([M+H+]), 451,09 cacld., 452.36 found.

*Synthesis of 4-(2-(5-bromopyrimidin-2-yl)-2,7-diazaspiro[3.5]nonan-7-yl)-6-(1-methyl-1H-pyrazol-4-yl)pyrrolo[2,1-f][1,2,4]triazine (**S8**).*

6-(1-methyl-1H-pyrazol-4-yl)-4-(2,7-diazaspiro[3.5]nonan-7-yl)pyrrolo[2,1-f][1,2,4]triazine (**S6**, 30.0 mg, 0.09 mmol, 1.0 eq.), 5-bromo-2-chloropyrimidine (17.9 mg, 0.09 mmol, 1.0 eq.) and TEA (19.4 µL, 0.14 mmol, 1.5 eq.) were used following common procedure C and yielded the final compound as a white solid (31.0 mg, 70 %.). **^{1}H NMR** (400 MHz, DMSO-d$_6$) δ ppm 1.79 - 1.84 (m, 4 H) 3.77 (s, 3 H) 3.78 (s, 4 H) 3.86 - 3.90 (m, 4 H) 7.07 (d, J=1.47 Hz, 1 H) 7.74 (s, 1 H) 7.77 (s, 1 H) 7.88 (d, J=1.47 Hz, 1 H) 7.97 (s, 1 H) 8.38 (s, 2 H). **^{13}C NMR** (101 MHz, DMSO-d$_6$) δ ppm 34.69 (s, 2 C) 35.53 (s, 1 C) 39.03 (s, 1 C) 43.40 (s, 2 C) 60.24 (s, 2 C) 100.00 (s, 1 C) 101.80 (s, 1 C) 106.51 (s, 1 C) 114.93 (s, 1 C) 116.23 (s, 1 C) 118.21 (s, 1 C) 128.07 (s, 1 C) 136.89 (s, 1 C) 147.00 (s, 1 C) 153.92 (s, 1 C) 158.45 (s, 2 C) 161.32 (s, 1 C). **HPLC-MS (ESI):** [Rt]: 5.70 min, m/z for $C_{21}H_{22}BrN_9$ ([M+H+]), 479.12 cacld., 480.33 found.

*Synthesis of 4-(4-(5-benzylpyrimidin-2-yl)piperazin-1-yl)-6-(1-methyl-1H-pyrazol-4-yl)pyrrolo[2,1-f][1,2,4]triazine (**5**).*

4-(4-(5-bromopyrimidin-2-yl)piperazin-1-yl)-6-(1-methyl-1H-pyrazol-4-yl)pyrrolo[2,1-f][1,2,4]triazine (**S7**, 25.0 mg, 0.1 mmol, 1.0 eq.), 2-benzyl-4,4,5,5-tetramethyl-1,3,2-dioxaborolane (19.0 µL, 0.1 mmol, 1.5 eq.), [1,1'-Bis(diphenylphosphino)ferrocene]palladium(II)dichloride (5.9 mg, 8.5 µmol, 0.15 eq.) and Cs_2CO_3 (55.7 mg, 0.2 mmol, 3.0 eq.) were used following common procedure D and yielded the final compound as a white solid (18.5 mg, 72 %). **^{1}H NMR** (600 MHz, DMSO-d6) δ ppm 3.81 (s, 2 H) 3.86 (s, 3 H) 3.89 - 3.92 (m, 4 H) 4.08 - 4.11 (m, 4 H) 7.18 - 7.23 (m, 2 H) 7.25 (d, J=6.97 Hz, 2 H) 7.30 (t, J=7.52 Hz, 2 H) 7.81 - 7.82 (m, 1 H) 7.87 (s, 1 H) 7.98 (d, J=1.56 Hz, 1 H) 8.03 (s, 1 H) 8.34 (s, 2 H). **^{13}C NMR** (151 MHz, DMSO-d_6) δ ppm 35.03 (s, 1 C) 39.04 (s, 1 C) 43.54 (s, 4 C) 102.13 (s, 1 C) 114.97 (s, 1 C) 115.91 (s, 1 C) 116.25 (s, 1 C) 118.29 (s, 1 C) 123.32 (s, 1 C) 126.61 (s, 1 C) 128.03 (s, 1 C) 128.85 (s, 2 C) 129.05 (s, 2 C) 136.86 (s, 1 C) 141.39 (s, 1 C) 146.97 (s, 1 C) 154.17 (s, 1 C) 158.32 (s, 2 C) 160.56 (s, 1 C). **HPLC-MS (ESI):** [Rt]: 6.55 min, m/z for $C_{25}H_{25}N_9$ ([M+H+]), 451,22 cacld., 452.35 found.

*Synthesis of 4-(6-(5-benzylpyrimidin-2-yl)-2,6-diazaspiro[3.3]heptan-2-yl)-6-(1-methyl-1H-pyrazol-4-yl)pyrrolo[2,1-f][1,2,4]triazine (**6**).*

4-(6-(5-bromopyrimidin-2-yl)-2,6-diazaspiro[3.3]heptan-2-yl)-6-(1-methyl-1H-pyrazol-4-yl)pyrrolo[2,1-f][1,2,4]triazine (**S8**, 13.0 mg, 0.03 mmol, 1.0 eq.), 2-benzyl-4,4,5,5-tetramethyl-1,3,2-dioxaborolane (0.01 mL, 0.04 mmol, 1.5 eq.), [1,1'-Bis(diphenylphosphino)ferrocene]palladium(II)dichloride (2.9 mg, 4.3 µmol, 0.15 eq.) and Cs_2CO_3 (28.2 mg, 0.09 mmol, 3.0 eq.) were used following common procedure D and yielded the final compound as a white solid (7.2 mg, 54 %). **^{1}H NMR** (700 MHz, DMSO-d_6) δ ppm 3.33 (s, 8 H) 3.85 (s, 2 H) 4.25 (s, 3 H) 6.84 (d, J=1.51 Hz, 1 H) 7.18 - 7.22 (m, 1 H) 7.23 (d, J=7.10 Hz, 2 H) 7.30 (t, J=7.64 Hz, 2 H) 7.77 (s, 1 H) 7.80 (s, 1 H) 7.92 (d, J=1.72 Hz, 1 H) 8.00 (s, 1 H) 8.29 (s, 2 H). **^{13}C NMR** (176 MHz, DMSO-d_6) δ ppm 34.36 (s, 1 C) 34.65 (s, 1 C) 38.54 (s, 1 C) 55.78 (s, 2 C) 60.12 (s, 2 C) 98.99 (s, 1 C) 113.98 (s, 1 C) 114.80 (s, 1 C) 115.82 (s, 1 C) 118.09 (s, 1 C) 123.36 (s, 1 C) 126.13 (s, 1 C) 127.43 (s, 1 C) 128.33 (s, 2 C) 128.56 (s, 2 C) 136.27 (s, 1 C) 140.92 (s, 1 C) 147.26 (s, 1 C) 153.26 (s, 1 C) 157.74 (s, 2 C) 161.81 (s, 1 C). **HPLC-MS (ESI):** [Rt]: 4.65 min, m/z for $C_{26}H_{25}N_9$ ([M+H+]), 463,22 cacld., 464.29 found.

*Synthesis of 4-(2-(5-benzylpyrimidin-2-yl)-2,7-diazaspiro[3.5]nonan-7-yl)-6-(1-methyl-1H-pyrazol-4-yl)pyrrolo[2,1-f][1,2,4]triazine (**7**).*

4-(2-(5-bromopyrimidin-2-yl)-2,7-diazaspiro[3.5]nonan-7-yl)-6-(1-methyl-1H-pyrazol-4-yl)pyrrolo[2,1-f][1,2,4]triazine (**S9**, 28.0 mg, 0.06 mmol, 1.0 eq.), 2-benzyl-4,4,5,5-tetramethyl-1,3,2-dioxaborolane (0.02 mL, 0.09 mmol, 1.5 eq.), [1,1'-Bis(diphenylphosphino)ferrocene]palladium(II)dichloride (6.1 mg, 0.01 mmol, 0.15 eq.) and Cs_2CO_3 (57.2 mg, 0.17 mmol, 3.0 eq.) were used following common procedure D and yielded the final compound as a white solid (15.5 mg, 54 %).**^{1}H NMR** (700 MHz, DMSO-d_6) δ ppm 1.86 - 1.89 (m, 4 H) 3.79 (s, 2 H) 3.82 (s, 4 H) 3.85 (s, 3 H) 3.94 - 3.97 (m, 4 H) 7.14 - 7.15 (m, 1 H) 7.18 - 7.21 (m, 1 H) 7.23 (d, J=7.10 Hz, 2 H) 7.28 - 7.31 (m, 2 H) 7.82 (s, 1 H) 7.85 (s, 1 H) 7.96 (d, J=1.29 Hz, 1 H) 8.05 (s, 1 H) 8.27 (s, 2 H). **^{13}C NMR** (176 MHz, DMSO-d_6) d ppm 34.16 (s, 2 C) 34.66 (s, 1 C) 35.16 (s, 1 C) 38.51 (s, 1 C) 40.01 (s, 2 C) 59.74 (s, 2 C) 101.28 (s, 1 C) 114.43 (s, 1 C) 115.35 (s, 1 C) 115.73 (s, 1 C) 117.69 (s, 1 C) 122.85 (s, 1 C) 126.08 (s, 1 C) 127.56 (s, 1 C) 128.32 (s, 2 C) 128.53 (s, 2 C) 136.37 (s, 1 C) 140.96 (s, 1 C) 146.49 (s, 1 C) 153.39 (s, 1 C) 157.71 (s, 2 C) 161.88 (s, 1 C). **HPLC-MS (ESI):** [Rt]: 5.77 min, m/z for $C_{28}H_{29}N_9$ ([M+H+]), 491.25 cacld., 492.39 found.

Synthesis of (S)-1-(4-fluorophenyl)-1-(2-(4-(6-(1-methyl-1H-pyrazol-4-yl)pyrrolo[2,1-f][1,2,4]triazin-4-yl)piperazin-1-yl)pyrimidin-5-yl)ethan-1-amine (**1**) has been reported. [7]
^{1}H NMR (400 MHz, DMSO-*d*$_6$) *δ* ppm 1.66 (s, 3 H) 3.78 (s, 3 H) 3.81 - 3.85 (m, 4 H) 4.01 (dd, *J*=6.60, 4.16 Hz, 4 H) 7.00 - 7.06 (m, 2 H) 7.15 (d, *J*=1.47 Hz, 1 H) 7.39 (dd, *J*=8.80, 5.38 Hz, 2 H) 7.74 (s, 1 H) 7.79 (s, 1 H) 7.90 (d, *J*=1.47 Hz, 1 H) 7.95 (s, 1 H) 8.33 (s, 2 H). **^{13}C NMR** (101 MHz, DMSO-*d*$_6$) *δ* ppm 31.74 (s, 1 C) 39.05 (s, 1 C) 43.53 (s, 4 C) 55.53 (s, 1 C) 102.14 (s, 1 C) 114.84 (s, 1 C) 114.97 (s, 1 C) 115.04 (s, 1 C) 115.92 (s, 1 C) 116.27 (s, 1 C) 118.31 (s, 1 C) 128.03 (s, 1 C) 128.42 (s, 1 C) 128.50 (s, 2 C) 132.14 (s, 1 C) 136.87 (s, 1 C) 146.40 (s, 1 C) 146.97 (s, 1 C) 154.17 (s, 1 C) 156.53 (s, 2 C) 160.19 (s, 1 C). **^{19}F NMR** (377 MHz, CHLOROFORM-*d*) δ ppm -115.92 (s, 1 F). **HPLC-MS (ESI):** [R_t]: 3.30 min, *m/z* for $C_{26}H_{27}FN_{10}$ ([M+H$^+$]), 498.24 cacld., 498.99 found.

Synthesis of (S)-1-(4-fluorophenyl)-N,N-dimethyl-1-(2-(4-(6-(1-methyl-1H-pyrazol-4-yl)pyrrolo[2,1-f][1,2,4]triazin-4-yl)piperazin-1-yl)pyrimidin-5-yl)ethan-1-amine (**8**).
Methyliodide (11.25 µL, 0.18 mmol) was slowly added to the stirred solution of **1** (30 mg, 0.06 mmol) and DIPEA (16 µL, 0.09 mmol) in dry THF (3 mL) at 0 °C and the reaction mixture was allowed to stir for 3 h at room temperature. After the completion of the reaction was monitored via TLC and LC-MS, the reaction was quenched by adding sat. $NaHCO_3$ and three times extracted with EA. The combined organic fractions were dried over Na_2SO_4, filtered, and concentrated *in vacuo*. The crude product was absorbed onto silica gel and purified by flash column chromatography (DCM/MeOH) to yield the titled compound (**8**) as off-white solid (24 mg, 77 %). **^{1}H NMR** (600 MHz, DMSO-*d*$_6$) δ ppm 1.69 (s, 3 H) 2.07 (s, 6 H) 3.86 (s, 3 H) 3.92 (dd, *J*=6.12, 4.47 Hz, 4 H) 4.08 - 4.12 (m, 4 H) 7.11 - 7.15 (m, 2 H) 7.22 (d, *J*=1.60 Hz, 1 H) 7.48 - 7.51 (m, 2 H) 7.82 (s, 1 H) 7.87 (s, 1 H) 7.98 (d, *J*=1.56 Hz, 1 H) 8.04 (s, 1 H) 8.42 (s, 2 H). **^{13}C NMR** (151 MHz, DMSO-*d*$_6$) δ ppm 18.35 (s, 1 C) 39.04 (s, 1 C) 40.53 (s, 1 C) 43.39 (s, 4 C) 63.99 (s, 1 C) 102.13 (s, 1 C) 114.96 (s, 1 C) 115.18 (s, 1 C) 115.32 (s, 1 C) 115.93 (s, 1 C) 116.26 (s, 1 C) 118.30 (s, 1 C) 126.77 (s, 1 C) 128.02 (s, 2 C) 129.35 (s, 1 C) 136.87 (s, 1 C) 142.50 (s, 1 C) 142.52 (s, 1 C) 146.97 (s, 1 C) 154.16 (s, 1 C) 157.50 (s, 2 C) 160.11 (s, 1 C) 161.88 (s, 1 C). **^{19}F NMR** (470 MHz, DMSO-*d*$_6$) δ ppm -116.83 (s, 1 F). **HPLC-MS (ESI):** [R_t]: 5.65 min, *m/z* for $C_{28}H_{31}FN_{10}$ ([M+H$^+$]), 526.27 cacld., 527.33, found.

Synthesis of (S)-N-ethyl-1-(4-fluorophenyl)-1-(2-(4-(6-(1-methyl-1H-pyrazol-4-yl)pyrrolo[2,1-f][1,2,4]triazin-4-yl)piperazin-1-yl)pyrimidin-5-yl)ethan-1-amine (**9**).
Ethyliodide (10 µL, 0.10 mmol, 1.2 eq.) was slowly added to the stirred solution of **1** (40 mg, 0.08 mmol, 1.0 eq.) and freshly washed NaH (60 % in oil) (8 mg, 0.32 mmol, 4.0 eq.) in dry THF (5 mL) at 0 °C and the reaction mixture was allowed to stir for 8 h at room temperature. After the completion of the reaction was monitored via TLC and LC-MS, the reaction was quenched by adding water at 0 °C and extracted with EA three times. The combined organic layers were dried over Na_2SO_4, filtered, and concentrated under reduced pressure. The crude product was absorbed onto silica gel and purified by flash column chromatography, pure fractions were evaporated to dryness to afford the titled compound **9** as off-white solid (34 mg, 80 %). **^{1}H NMR** (500 MHz, DMSO-*d*$_6$) δ ppm 1.02 (t, *J*=7.02 Hz, 3 H) 1.69 (s, 3 H) 2.50 - 2.51 (m, 2 H) 3.86 (s, 3 H) 3.89 - 3.94 (m, 4 H) 4.08 - 4.12 (m, 4 H) 7.12 (t, *J*=8.85 Hz, 2 H) 7.24 (d, *J*=1.37 Hz, 1 H) 7.42 (dd, *J*=8.77, 5.57 Hz, 2 H) 7.83 (s, 1 H) 7.88 (s, 1 H) 7.99 (d, *J*=1.37 Hz, 1 H) 8.04 (s, 1 H) 8.34 (s, 2 H). **^{13}C NMR** (126 MHz, DMSO-*d*$_6$) δ ppm 15.99 (s, 1 C) 27.34 (s, 1 C) 37.24 (s, 1 C) 39.04 (s, 1 C) 43.44 (s, 4 C) 59.53 (s, 1 C) 102.13 (s, 1 C) 114.95 (s, 1 C) 115.15 (s, 1 C) 115.92 (s, 1 C) 116.27 (s, 1 C) 118.30 (s, 1 C) 128.02 (s, 1 C) 129.05 (s, 1 C) 129.12 (s, 1 C) 129.56 (s, 2 C) 136.86 (s, 1 C) 144.30 (s, 1 C) 146.97 (s, 1 C) 154.14 (s, 1 C) 157.20 (s, 2 C) 160.24 (s, 1 C) 162.09 (s, 1 C). **^{19}F NMR** (470 MHz, DMSO-*d*$_6$) δ ppm -117.05 (s, 1 F). **HPLC-MS (ESI):** [R_t]: 3.84 min, *m/z* for $C_{28}H_{31}FN_{10}$ ([M+H$^+$]), 526.27 cacld., 527.96, found.

*Synthesis of (S)-N-(1-(4-fluorophenyl)-1-(2-(4-(6-(1-methyl-1H-pyrazol-4-yl)pyrrolo[2,1-f][1,2,4]triazin-4-yl)piperazin-1-yl)pyrimidin-5-yl)ethyl)acetamide (**10**).*
Acetylchloride (4.2 µL, 0.06 mmol, 1.0 eq.) was slowly added to the stirred solution of **1** (30 mg, 0.06 mmol, 1.0 eq.) in dry THF (3 mL) at 0 °C and the reaction mixture was allowed to stir for 3 h at room temperature. After the completion of the reaction was monitored via TLC and LC-MS, the reaction was quenched by adding sat. $NaHCO_3$ (5 mL) solution. Followed by the extraction two times with EA, washed with brine solution. The organic fractions were concentrated and purified by flash chromatography to get the title product **10** as off-white solid (29 mg, 89 %). **^{1}H NMR** (500 MHz, $CDCl_3$-*d*) δ ppm 1.93 (s, 3 H) 2.01 (s, 3 H) 3.87 (s, 3 H) 3.94 (dd, *J*=6.26, 4.27 Hz, 4 H) 4.04 - 4.09 (m, 4 H) 6.06 (s, 1 H) 6.70 (d, *J*=1.53 Hz, 1 H) 6.95 (t, *J*=8.54 Hz, 2 H) 7.15 - 7.20 (m, 2 H) 7.49 (s, 1 H) 7.60 - 7.63 (m, 2 H) 7.82 (s, 1 H) 8.22 (s, 2 H). **^{13}C NMR** (126 MHz, $CDCl_3$-*d*) δ ppm 24.24 (s, 1 C) 28.01 (s, 1 C) 39.07 (s, 1 C) 43.29 (s, 1 C) 45.39 (s, 1 C) 59.03 (s, 1 C) 101.26 (s, 1 C) 115.41 (s, 2 C) 115.58 (s, 1 C) 115.90 (s, 1 C) 116.41 (s, 1 C) 117.78 (s, 1 C) 126.57 (s, 1 C) 126.92 (s, 1 C) 128.03 (s, 2 C) 136.98 (s, 1 C) 140.58 (s, 1 C) 146.84 (s, 1 C) 154.52 (s, 1 C) 156.70 (s, 2 C) 160.28 (s, 1 C) 160.93 (s, 1 C) 162.90 (s, 1 C) 169.22 (s, 1 C). **^{19}F NMR** (470 MHz, DMSO-d_6) δ ppm -117.29 (s, 1 F). **HPLC-MS (ESI):** [R_t]: 5.30 min, *m/z* for $C_{28}H_{29}FN_{10}O$ ([M+H$^+$]), 540.25 cacld., 541.15 found.

*Synthesis of (S)-N-(1-(4-fluorophenyl)-1-(2-(4-(6-(1-methyl-1H-pyrazol-4-yl)pyrrolo[2,1-f][1,2,4]triazin-4-yl)piperazin-1-yl)pyrimidin-5-yl)ethyl)acrylamide (**11**).*
Acryloylchloride (4.9 µL, 0.06 mmol, 1.0 eq.) was slowly added to the stirred solution of **1** (30 mg, 0.06 mmol, 1.0 eq.) in dry THF (3 mL) at 0 °C and the reaction mixture was allowed to stir for 3 h at room temperature. After the completion of the reaction was monitored via TLC and LC-MS, the reaction was quenched by adding sat. $NaHCO_3$ (5 mL) solution and extracted with EA three times. The combined organic fractions were dried over Na_2SO_4, filtered, and concentrated *in vacuo*. The crude product was absorbed onto silica gel and purified by flash column chromatography (DCM/MeOH) to yield the desired product **11** as off-white solid (29 mg, 90 %). **^{1}H NMR** (400 MHz, $CDCl_3$-*d*) δ ppm 2.07 (s, 3 H) 3.87 (s, 3 H) 3.94 - 3.98 (m, 4 H) 4.08 (dd, *J*=6.36, 3.91 Hz, 4 H) 5.59 (dd, *J*=10.27, 1.47 Hz, 1 H) 6.01 - 6.11 (m, 1 H) 6.17 - 6.23 (m, 1 H) 6.72 (d, *J*=1.47 Hz, 1 H) 6.93 - 6.99 (m, 2 H) 7.16 - 7.23 (m, 3 H) 7.50 (s, 1 H) 7.62 (s, 2 H) 7.83 (s, 1 H) 8.24 (s, 2 H). **^{13}C NMR** (101 MHz, $CDCl_3$-*d*) δ ppm 28.11 (s, 1 C) 39.08 (s, 1 C) 43.30 (s, 2 C) 45.55 (s, 2 C) 59.15 (s, 1 C) 115.48 (s, 2 C) 115.70 (s, 1 C) 116.36 (s, 1 C) 122.76 (s, 1 C) 126.47 (s, 1 C) 126.92 (s, 1 C) 127.49 (s, 1 C) 128.02 (s, 1 C) 128.10 (s, 2 C) 130.80 (s, 1 C) 131.09 (s, 1 C) 137.03 (s, 1 C) 137.99 (s, 1 C) 140.40 (s, 1 C) 144.50 (s, 1 C) 156.73 (s, 2 C) 160.20 (s, 1 C) 163.22 (s, 1 C) 164.47 (s, 1 C). **^{19}F NMR** (470 MHz, DMSO-d_6) δ ppm -116.53 (s, 1 F). **HPLC-MS (ESI):** [R_t]: 5.66 min, *m/z* for $C_{29}H_{29}FN_{10}O$ ([M+H$^+$]), 552.25 cacld., 553.14 found.

*Synthesis of Methyl (S)-(1-(4-fluorophenyl)-1-(2-(4-(6-(1-methyl-1H-pyrazol-4-yl)pyrrolo[2,1-f][1,2,4]triazin-4-yl)piperazin-1-yl)pyrimidin-5-yl)ethyl)carbamate (**12**).*
Methyl chloroformate (0.01 µL, 0.1 mmol, 1.0 eq.) was slowly added to a solution of **1** (50.0 mg, 0.1 mmol, 1.0 eq.) and K_2CO_3 (13.9 mg, 0.1 mmol, 1.0 eq.) in DCM at 0 °C. The reaction mixture was stirred for 5 h at room temperature. Afterwards the suspension was diluted with water and extracted with DCM three times. The combined organic fractions were dried over Na_2SO_4, filtered, and concentrated *in vacuo*. The crude product was absorbed onto silica gel and purified by flash column chromatography (DCM/MeOH) to yield the desired product **12** as white solid (46.8 mg, 84 %). **^{1}H NMR** (500 MHz, DMSO-d_6) δ ppm 1.92 (s, 3 H) 3.48 (s, 3 H) 3.86 (s, 3 H) 3.93 (dd, *J*=6.18, 4.35 Hz, 4 H) 4.09 - 4.13 (m, 4 H) 7.16 (t, *J*=8.93 Hz, 2 H) 7.23 (d, *J*=1.53 Hz, 1 H) 7.30 - 7.34 (m, 2 H) 7.82 (s, 1 H) 7.88 (s, 1 H) 7.92 (s, 1 H) 7.99 (d, *J*=1.53 Hz, 1 H) 8.04 (s, 1 H) 8.28 (s, 2 H). **^{13}C NMR** (126 MHz, DMSO-d_6) δ ppm 22.96 (s, 1 C) 39.05 (s, 1 C) 43.41 (s, 4 C) 51.56 (s, 1 C) 58.15 (s, 1 C) 102.14 (s, 1 C) 114.98 (s, 1

C) 115.14 (s, 1 C) 115.31 (s, 1 C) 115.94 (s, 1 C) 116.26 (s, 1 C) 118.32 (s, 1 C) 127.75 (s, 1 C) 128.03 (s, 1 C) 128.80 (s, 2 C) 128.86 (s, 1 C) 136.87 (s, 1 C) 142.02 (s, 1 C) 146.97 (s, 1 C) 154.18 (s, 1 C) 155.78 (s, 1 C) 157.17 (s, 2 C) 160.21 (s, 1 C). **^{19}F NMR** (471 MHz, DMSO-*d*$_6$) δ ppm -116.58 (s, 1 F). **HPLC-MS (ESI):** [R_t]: 5.92 min, *m/z* for $C_{28}H_{29}FN_{10}O_2$ ([$M+H^+$]), 556.25 cacld., 557.30 found.

*Synthesis of (S)-N-(1-(4-fluorophenyl)-1-(2-(4-(6-(1-methyl-1H-pyrazol-4-yl)pyrrolo[2,1-f][1,2,4]triazin-4-yl)piperazin-1-yl)pyrimidin-5-yl)ethyl)benzamide (**13**).*

Benzoic acid (11.0 µL, 0.1 mmol, 1.1 eq.) was dissolved in dry DCM and TEA (44 µL, 0.3 mmol, 3.0 eq.) was added. After 5 minutes, 1-[bis(dimethylamino)methylene]-1H-1,2,3-triazolo[4,5-b]pyridinium-3-oxide hexafluorophosphate (41.9 mg, 0.1 mmol, 1.1 eq.) was added to the stirred solution. The resulting mixture was stirred for 15 min before **1** (50.0 mg, 0.1 mmol, 1.0 eq.) was added. The reaction mixture was stirred at room temperature for 14 h. After the completion of the reaction was monitored by TLC and LC-MS, the reaction was diluted with brine and washed with DCM. The combined organic layers were dried over Na_2SO_4, filtered and concentrated under reduced pressure. The crude product was absorbed on silica gel and purified by flash column chromatography (DCM/MeOH), pure fractions were evaporated to dryness to afford the title product as a white solid (51.1 mg, 85 %). **^{1}H NMR** (600 MHz, DMSO-*d*$_6$) δ ppm 2.08 (s, 3 H) 3.86 (s, 3 H) 3.92 - 3.96 (m, 4 H) 4.10 - 4.13 (m, 4 H) 7.18 (t, *J*=8.83 Hz, 2 H) 7.24 (d, *J*=1.38 Hz, 1 H) 7.39 (dd, *J*=8.83, 5.39 Hz, 2 H) 7.44 - 7.48 (m, 2 H) 7.52 - 7.56 (m, 1 H) 7.82 (s, 1 H) 7.86 (d, *J*=7.20 Hz, 2 H) 7.88 (s, 1 H) 7.99 (d, *J*=1.42 Hz, 1 H) 8.04 (s, 1 H) 8.37 (s, 2 H) 8.76 (s, 1 H). **^{13}C NMR** (151 MHz, DMSO-*d*$_6$) δ ppm 38.55 (s, 1 C) 40.05 (s, 1 C) 42.93 (s, 4 C) 58.57 (s, 1 C) 101.65 (s, 1 C) 114.48 (s, 1 C) 114.65 (s, 1 C) 114.79 (s, 1 C) 115.44 (s, 2 C) 115.77 (s, 1 C) 117.83 (s, 1 C) 127.19 (s, 1 C) 127.54 (s, 1 C) 127.67 (s, 2 C) 128.09 (s, 2 C) 128.37 (s, 1 C) 128.42 (s, 1 C) 131.23 (s, 1 C) 135.01 (s, 1 C) 136.38 (s, 1 C) 141.26 (s, 1 C) 146.48 (s, 1 C) 153.69 (s, 1 C) 156.83 (s, 2 C) 159.69 (s, 1 C) 166.39 (s, 1 C). **^{19}F NMR** (471 MHz, DMSO-*d*$_6$) δ ppm -116.73 (s, 1 F). **HPLC-MS (ESI):** [R_t]: 6.54 min, *m/z* for *$C_{33}H_{31}FN_{10}O$* ([$M+H^+$]), *602,27* cacld., 603.13 found.

Supplementary Note 1: NMR and mass spectra of final compounds.

*^{1}H NMR spectrum of 4-chloro-6-(1-methyl-1H-pyrazol-4-yl)pyrrolo[2,1-f][1,2,4]triazine (**2**).*

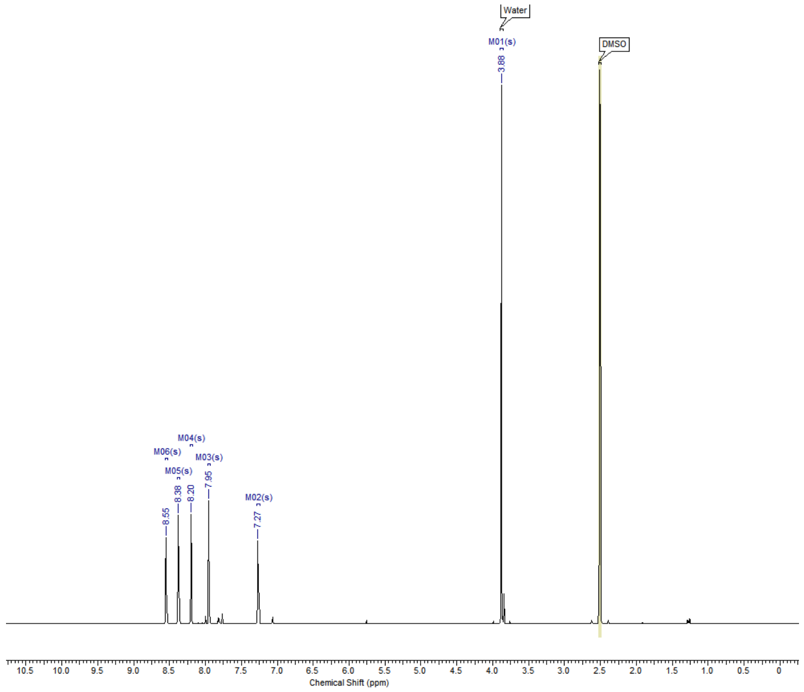

*^{13}C NMR spectrum of 4-chloro-6-(1-methyl-1H-pyrazol-4-yl)pyrrolo[2,1-f][1,2,4]triazine (**2**).*

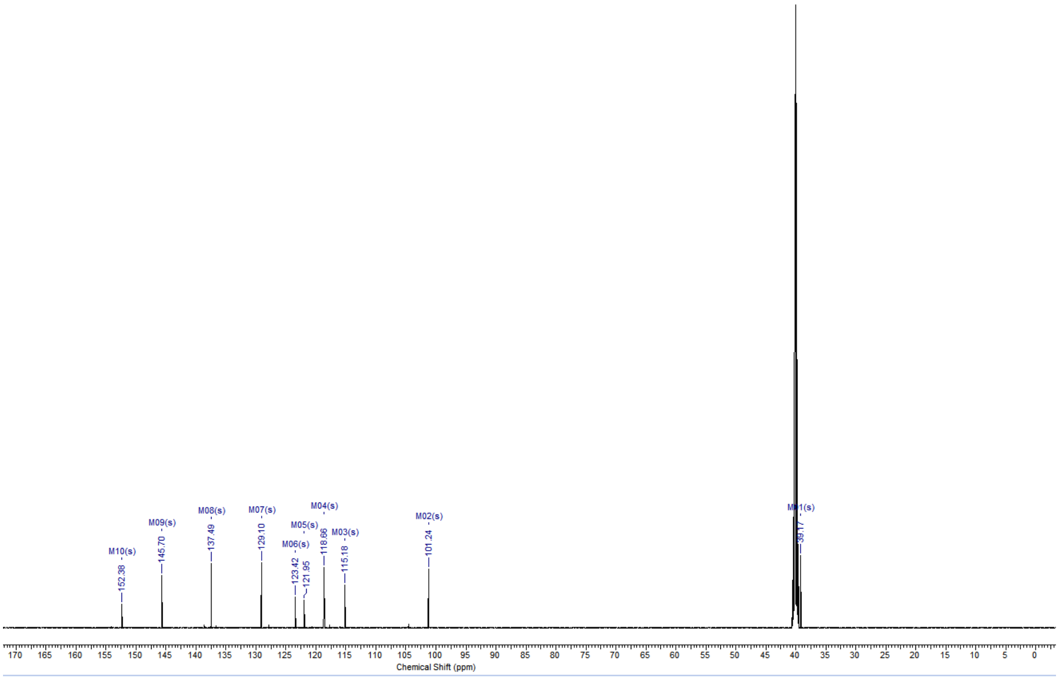

*HPLC spectrum of 4-chloro-6-(1-methyl-1H-pyrazol-4-yl)pyrrolo[2,1-f][1,2,4]triazine (**2**).*

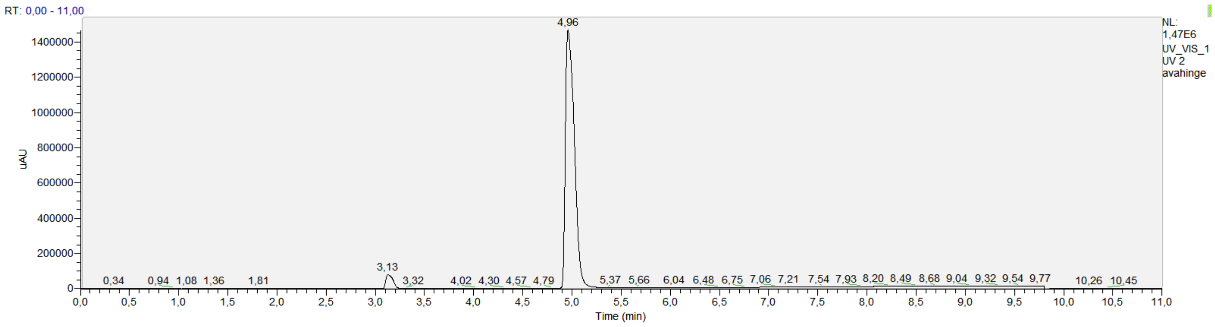

*^{1}H NMR spectrum of 6-(1-methyl-1H-pyrazol-4-yl)-4-(piperazin-1-yl)pyrrolo[2,1-f][1,2,4]triazine (**3**).*

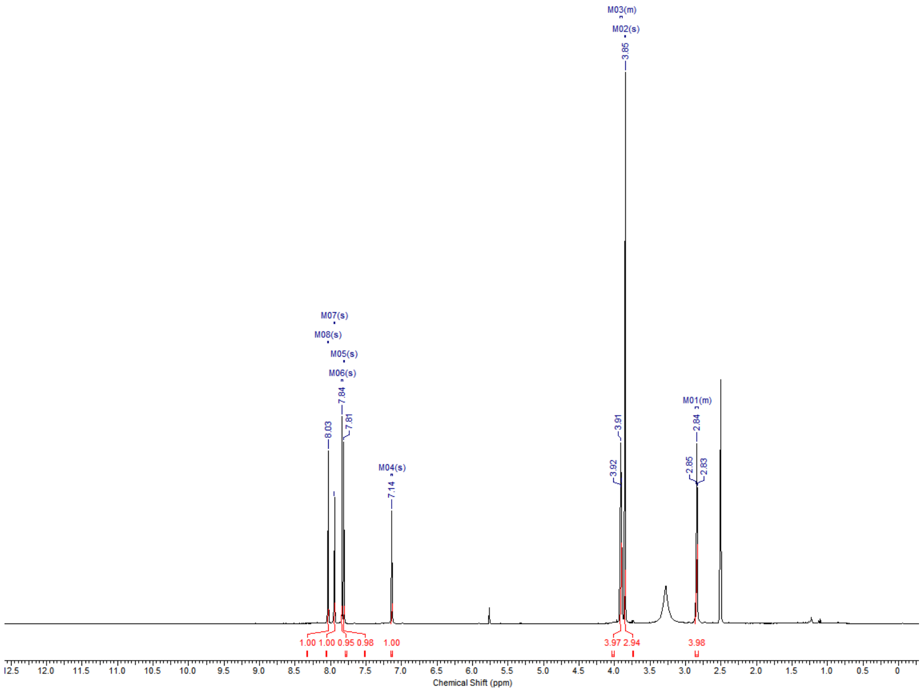

*^{13}C NMR spectrum of 6-(1-methyl-1H-pyrazol-4-yl)-4-(piperazin-1-yl)pyrrolo[2,1-f][1,2,4]triazine (**3**).*

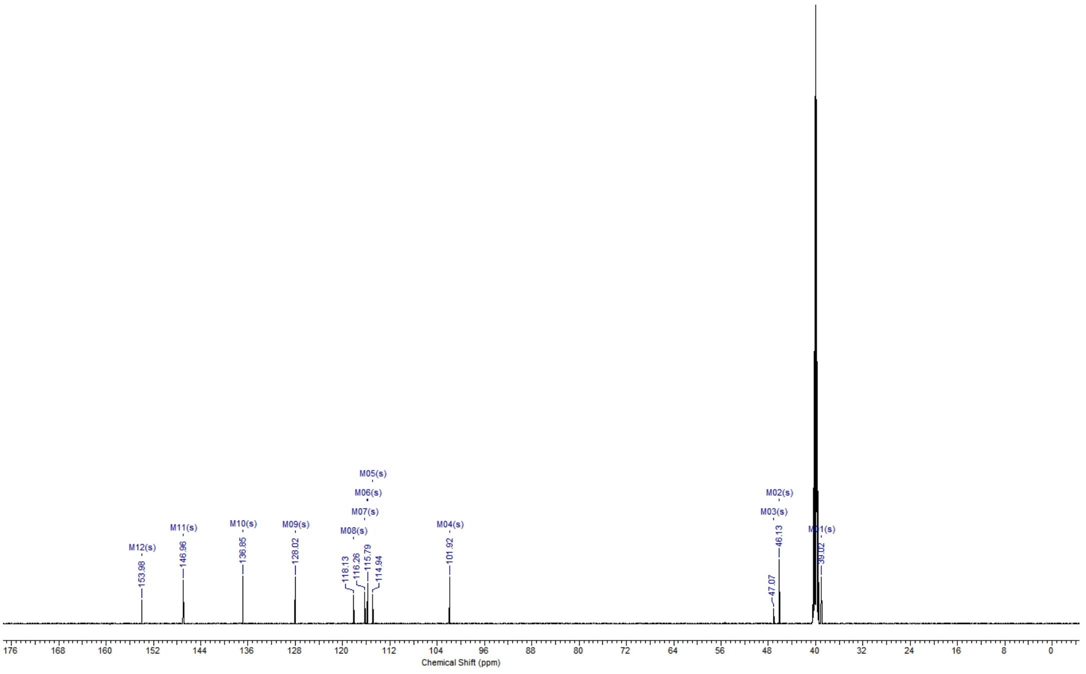

*HPLC spectrum of 6-(1-methyl-1H-pyrazol-4-yl)-4-(piperazin-1-yl)pyrrolo[2,1-f][1,2,4]triazine (**3**).*

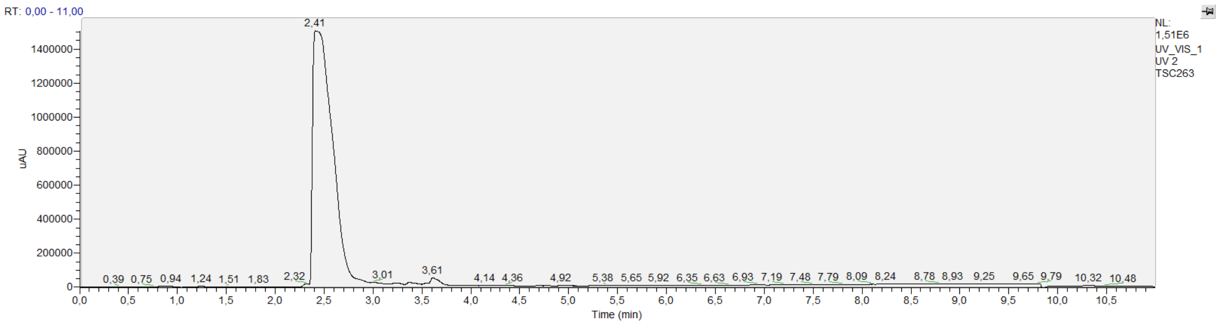

*^{1}H NMR spectrum of 2-(piperazin-1-yl)pyrimidine--6-(1-methyl-1H-pyrazol-4-yl)-4-(4-(pyrimidin-2-yl)piperazin-1-yl)pyrrolo[2,1-f][1,2,4]triazine (**4**).*

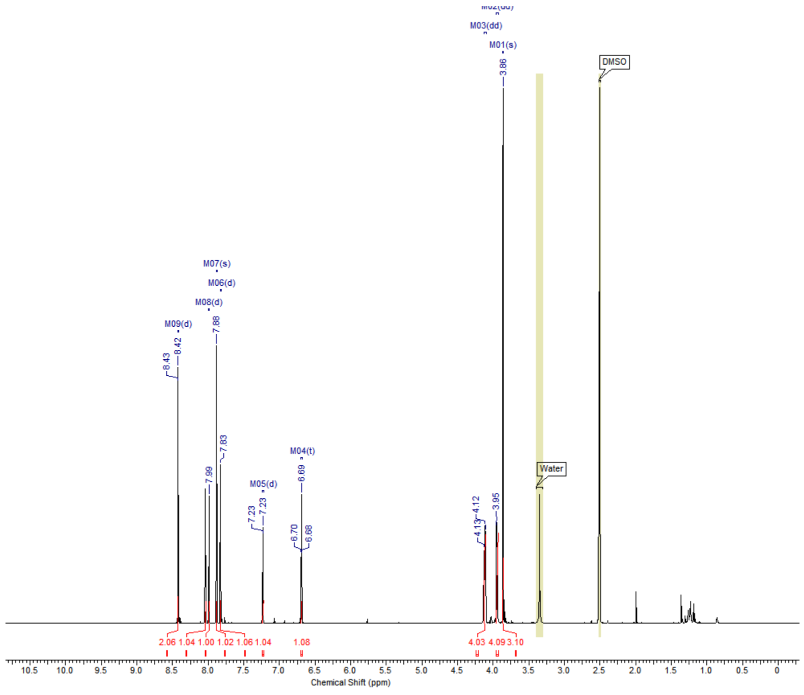

*^{13}C NMR spectrum of 2-(piperazin-1-yl)pyrimidine--6-(1-methyl-1H-pyrazol-4-yl)-4-(4-(pyrimidin-2-yl)piperazin-1-yl)pyrrolo[2,1-f][1,2,4]triazine (**4**).*

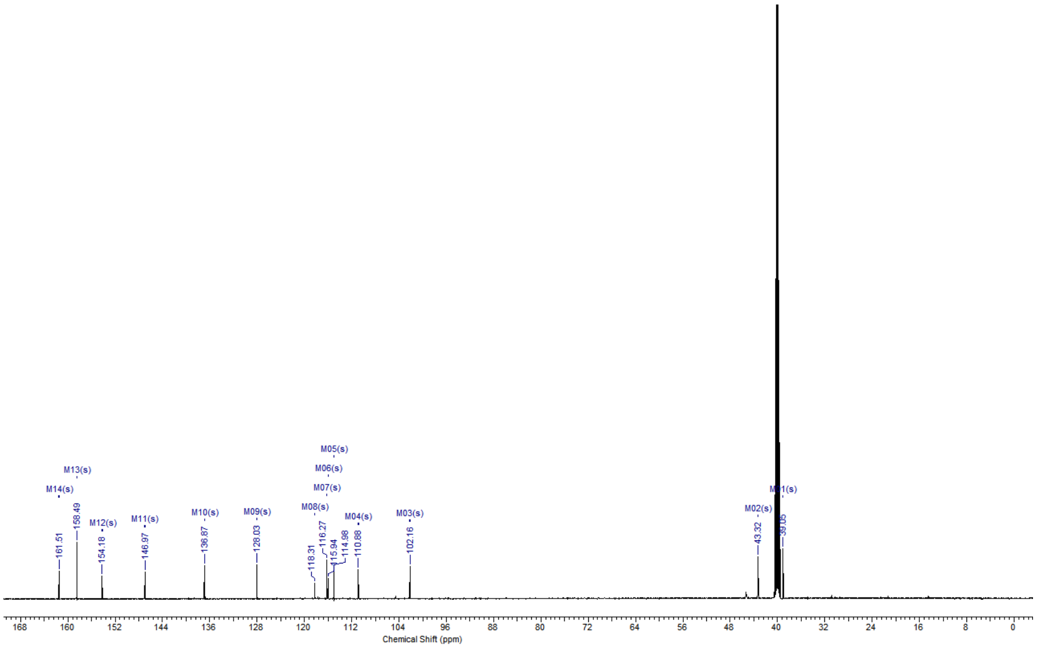

*HPLC spectrum of 2-(piperazin-1-yl)pyrimidine--6-(1-methyl-1H-pyrazol-4-yl)-4-(4-(pyrimidin-2-yl)piperazin-1-yl)pyrrolo[2,1-f][1,2,4]triazine (**4**).*

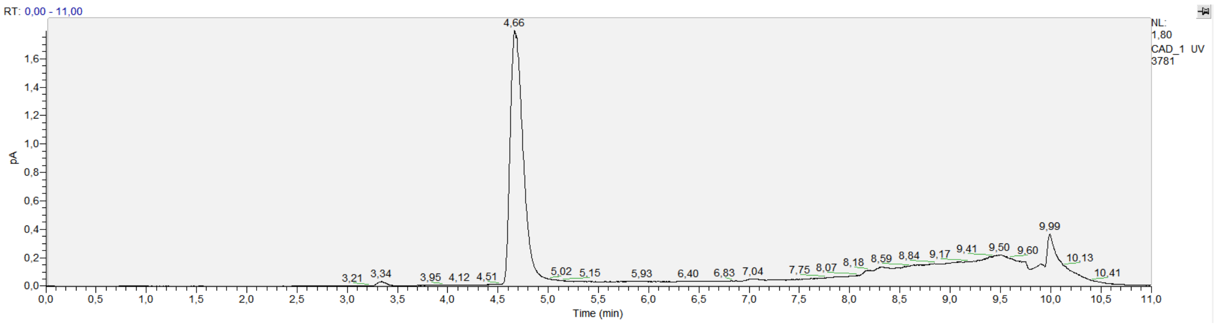

*^{1}H NMR spectrum of 4-(4-(5-benzylpyrimidin-2-yl)piperazin-1-yl)-6-(1-methyl-1H-pyrazol-4-yl)pyrrolo[2,1-f][1,2,4]triazine (**5**).*

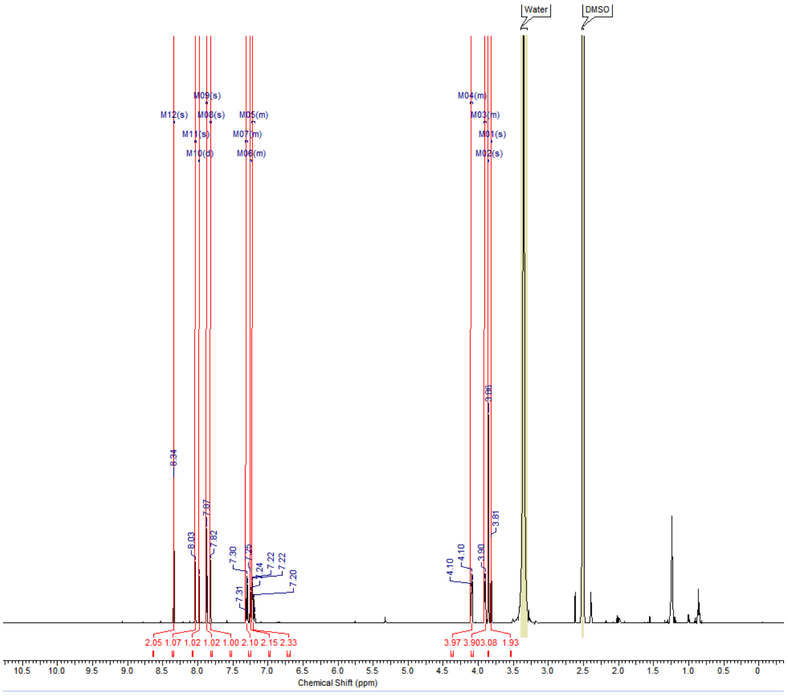

*^{13}C NMR spectrum of 4-(4-(5-benzylpyrimidin-2-yl)piperazin-1-yl)-6-(1-methyl-1H-pyrazol-4-yl)pyrrolo[2,1-f][1,2,4]triazine (**5**).*

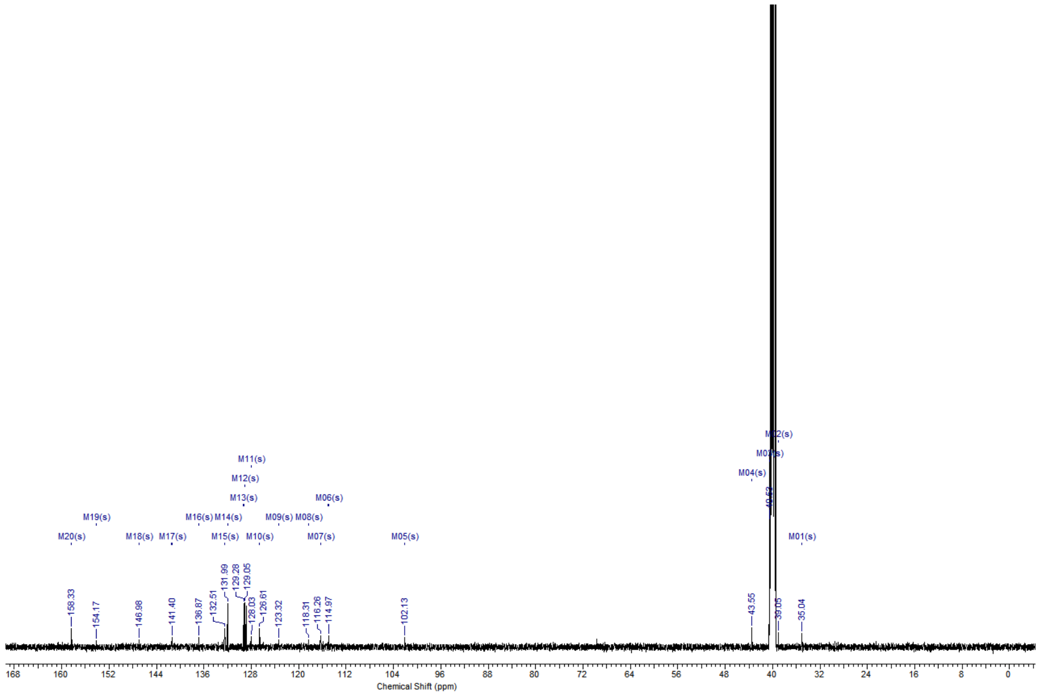

*HPLC spectrum of 4-(4-(5-benzylpyrimidin-2-yl)piperazin-1-yl)-6-(1-methyl-1H-pyrazol-4-yl)pyrrolo[2,1-f][1,2,4]triazine (**5**).*

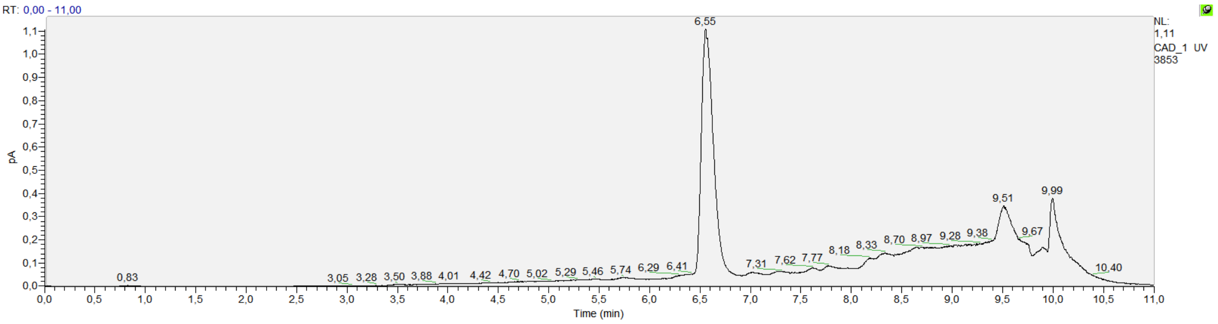

^{1}H NMR spectrum of 4-(6-(5-benzylpyrimidin-2-yl)-2,6-diazaspiro[3.3]heptan-2-yl)-6-(1-methyl-1H-pyrazol-4-yl)pyrrolo[2,1-f][1,2,4]triazine (**6**).

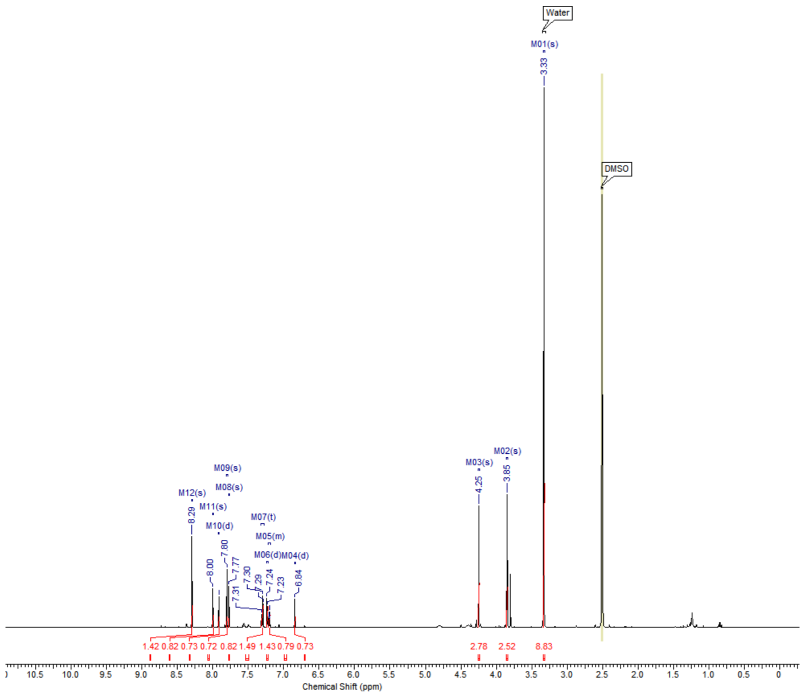

^{13}C NMR spectrum of 4-(6-(5-benzylpyrimidin-2-yl)-2,6-diazaspiro[3.3]heptan-2-yl)-6-(1-methyl-1H-pyrazol-4-yl)pyrrolo[2,1-f][1,2,4]triazine (**6**).

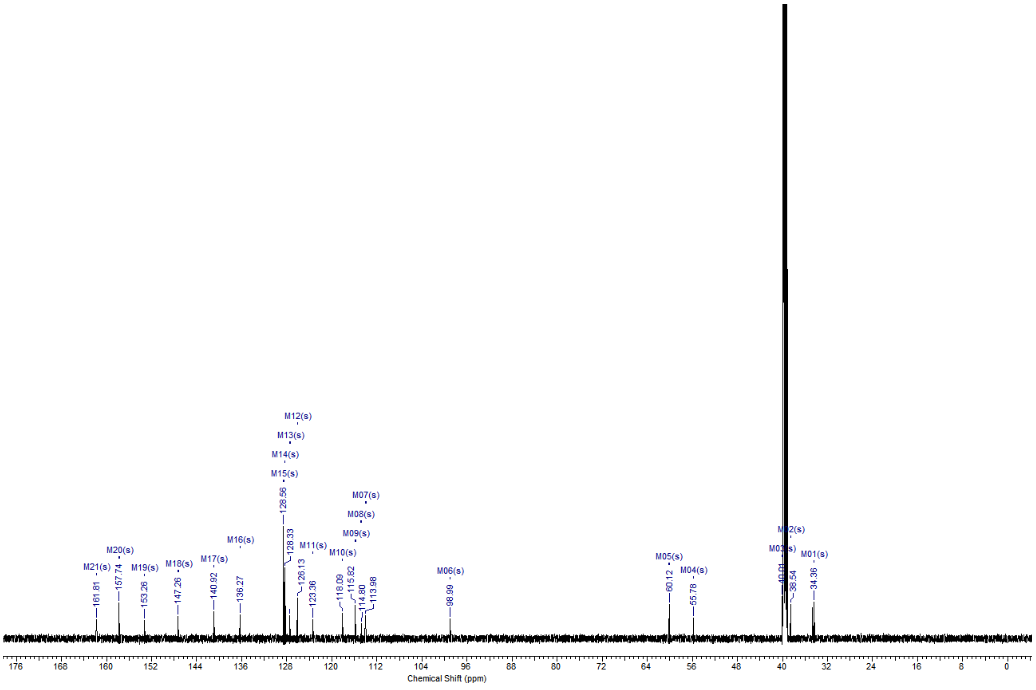

*HPLC spectrum of 4-(6-(5-benzylpyrimidin-2-yl)-2,6-diazaspiro[3.3]heptan-2-yl)-6-(1-methyl-1H-pyrazol-4-yl)pyrrolo[2,1-f][1,2,4]triazine (**6**).*

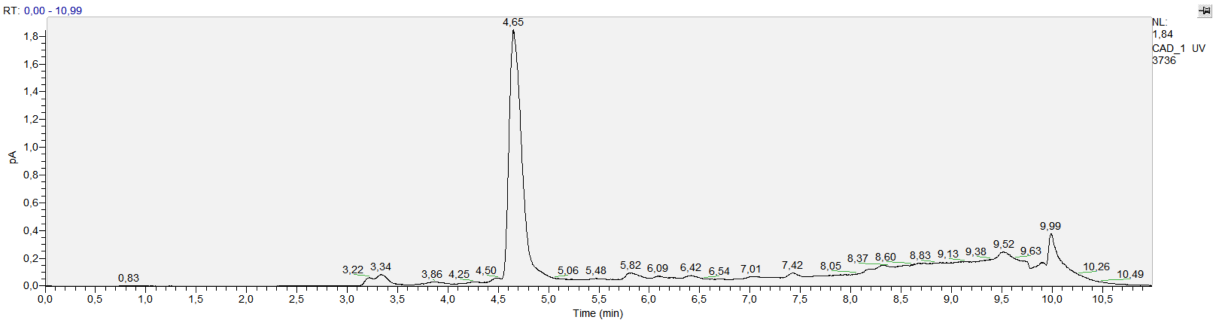

*^{1}H NMR spectrum of 4-(2-(5-benzylpyrimidin-2-yl)-2,7-diazaspiro[3.5]nonan-7-yl)-6-(1-methyl-1H-pyrazol-4-yl)pyrrolo[2,1-f][1,2,4]triazine (**7**).*

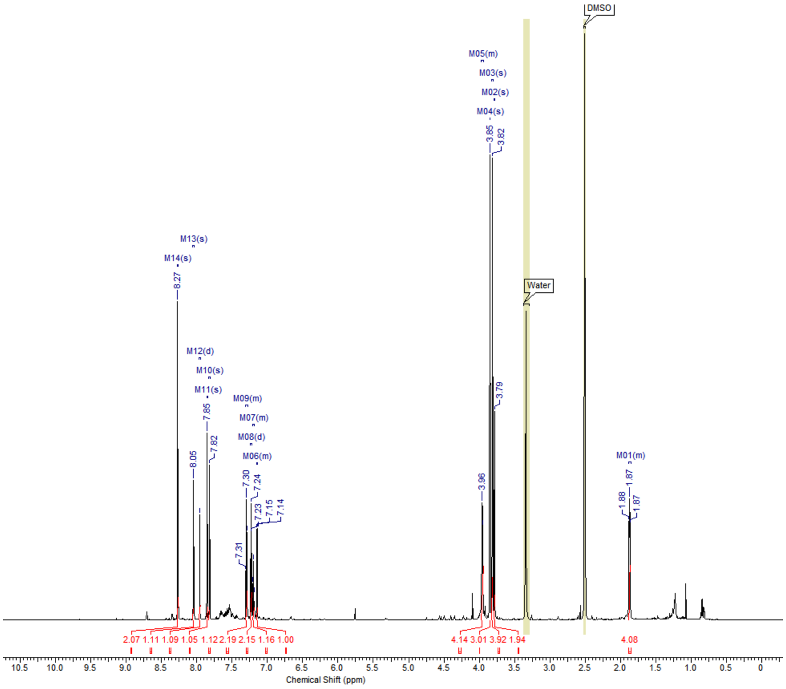

*^{13}C NMR spectrum of 4-(2-(5-benzylpyrimidin-2-yl)-2,7-diazaspiro[3.5]nonan-7-yl)-6-(1-methyl-1H-pyrazol-4-yl)pyrrolo[2,1-f][1,2,4]triazine (**7**).*

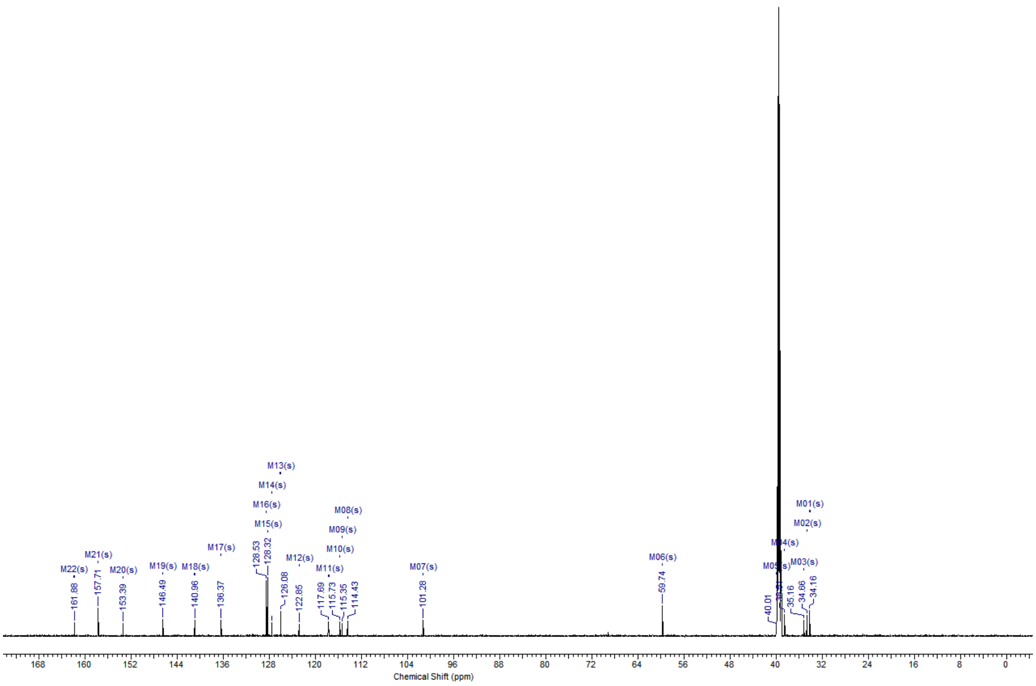

*HPLC spectrum of 4-(2-(5-benzylpyrimidin-2-yl)-2,7-diazaspiro[3.5]nonan-7-yl)-6-(1-methyl-1H-pyrazol-4-yl)pyrrolo[2,1-f][1,2,4]triazine (**7**).*

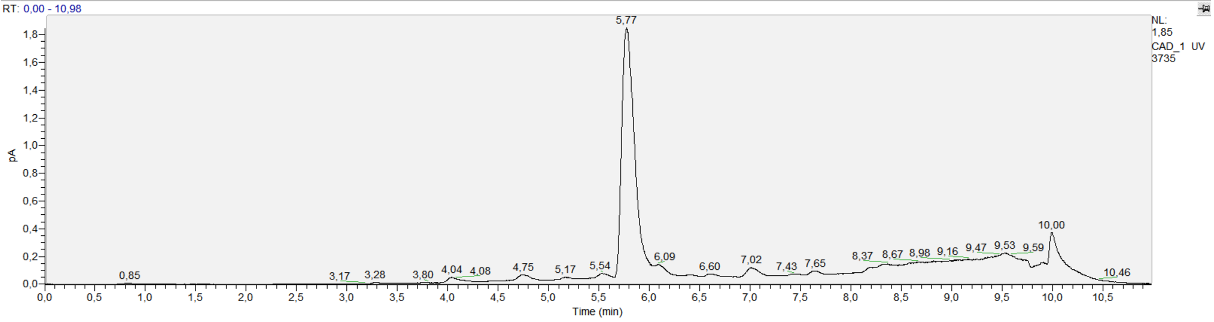

^{1}H NMR spectrum of (S)-1-(4-fluorophenyl)-1-(2-(4-(6-(1-methyl-1H-pyrazol-4-yl)pyrrolo[2,1-f][1,2,4]triazin-4-yl)piperazin-1-yl)pyrimidin-5-yl)ethan-1-amine (**1**).

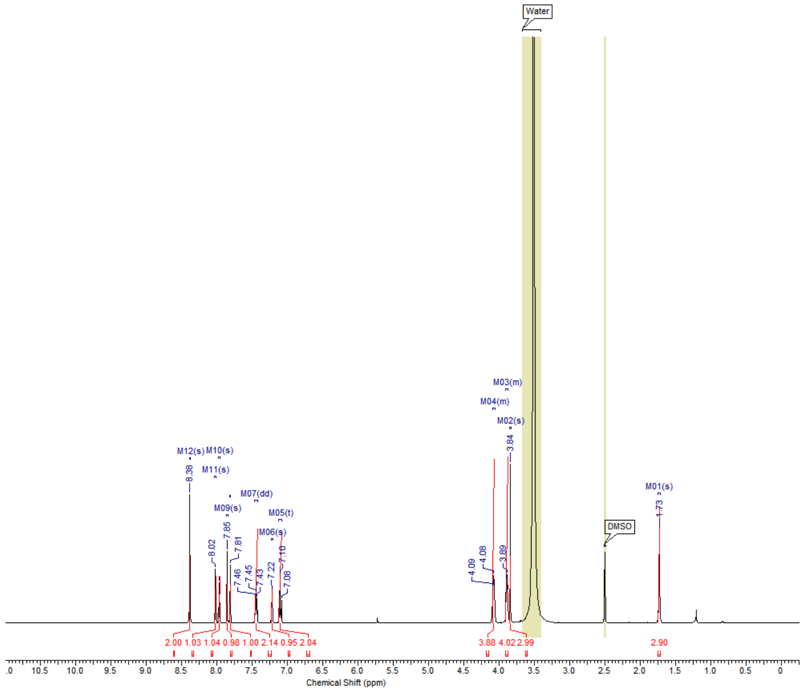

^{13}C NMR spectrum of (S)-1-(4-fluorophenyl)-1-(2-(4-(6-(1-methyl-1H-pyrazol-4-yl)pyrrolo[2,1-f][1,2,4]triazin-4-yl)piperazin-1-yl)pyrimidin-5-yl)ethan-1-amine (**1**).

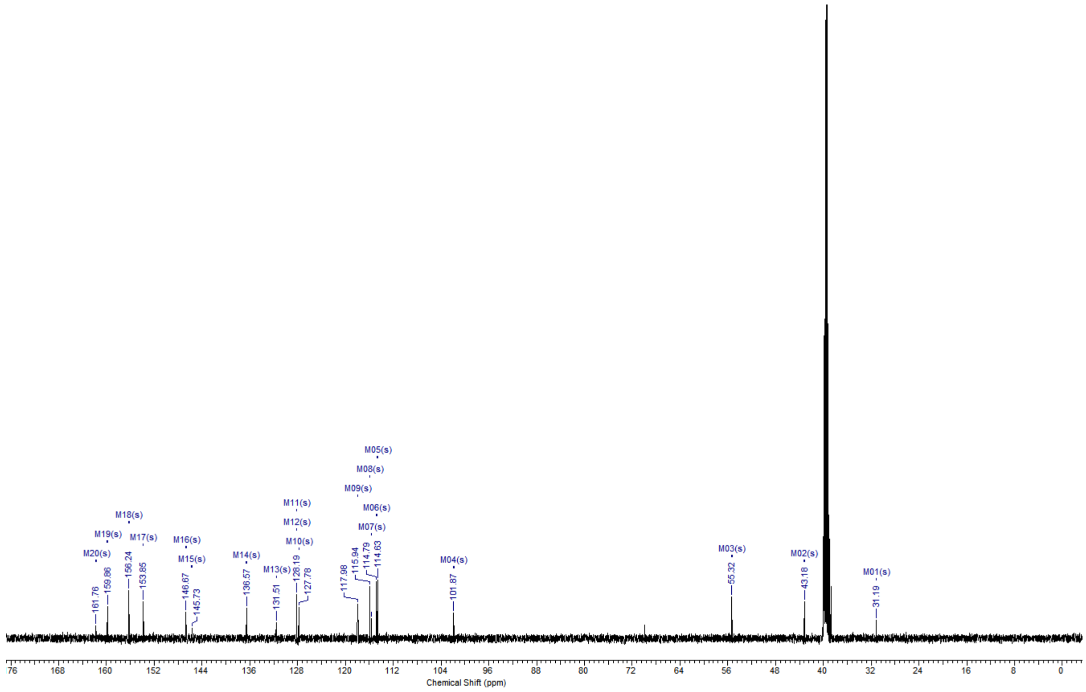

HPLC spectrum of (S)-1-(4-fluorophenyl)-1-(2-(4-(6-(1-methyl-1H-pyrazol-4-yl)pyrrolo[2,1-f][1,2,4]triazin-4-yl)piperazin-1-yl)pyrimidin-5-yl)ethan-1-amine (**1**).

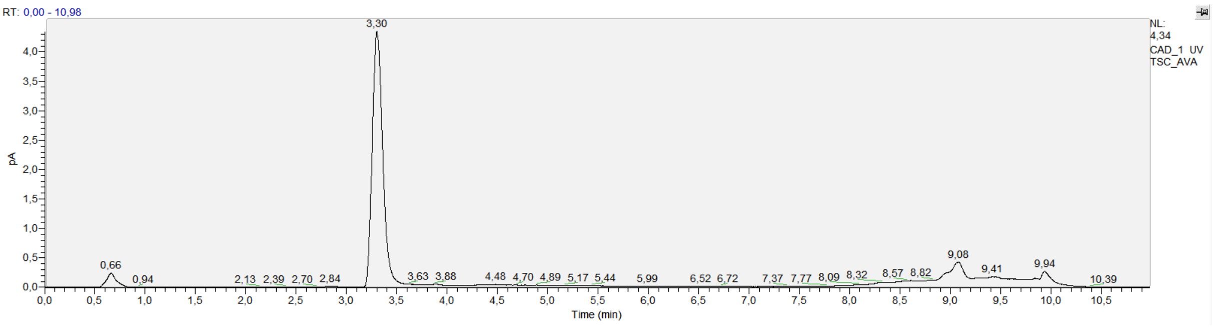

[1]*H NMR spectrum of (S)-1-(4-fluorophenyl)-N,N-dimethyl-1-(2-(4-(6-(1-methyl-1H-pyrazol-4-yl)pyrrolo[2,1-f][1,2,4]triazin-4-yl)piperazin-1-yl)pyrimidin-5-yl)ethan-1-amine* (**8**).

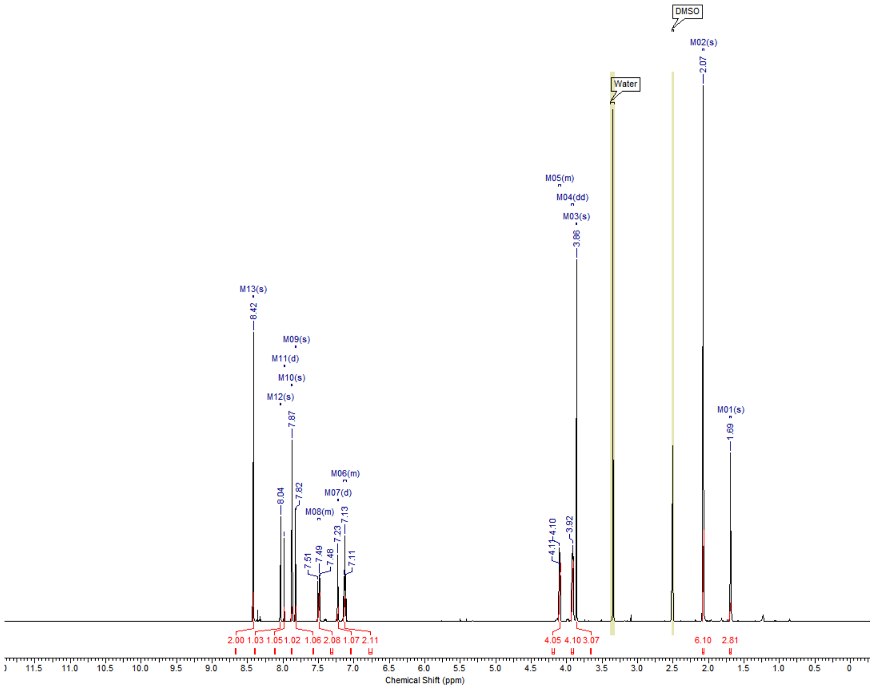

^{13}C NMR spectrum of (S)-1-(4-fluorophenyl)-N,N-dimethyl-1-(2-(4-(6-(1-methyl-1H-pyrazol-4-yl)pyrrolo[2,1-f][1,2,4]triazin-4-yl)piperazin-1-yl)pyrimidin-5-yl)ethan-1-amine (**8**).

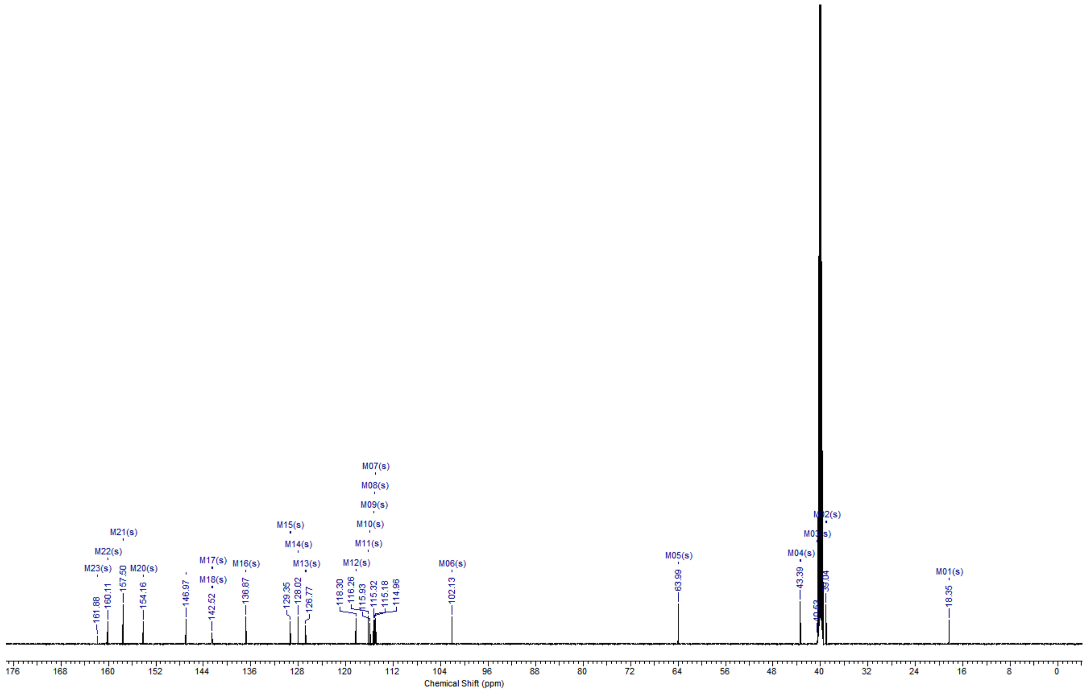

HPLC spectrum of (S)-1-(4-fluorophenyl)-N,N-dimethyl-1-(2-(4-(6-(1-methyl-1H-pyrazol-4-yl)pyrrolo[2,1-f][1,2,4]triazin-4-yl)piperazin-1-yl)pyrimidin-5-yl)ethan-1-amine (**8**).

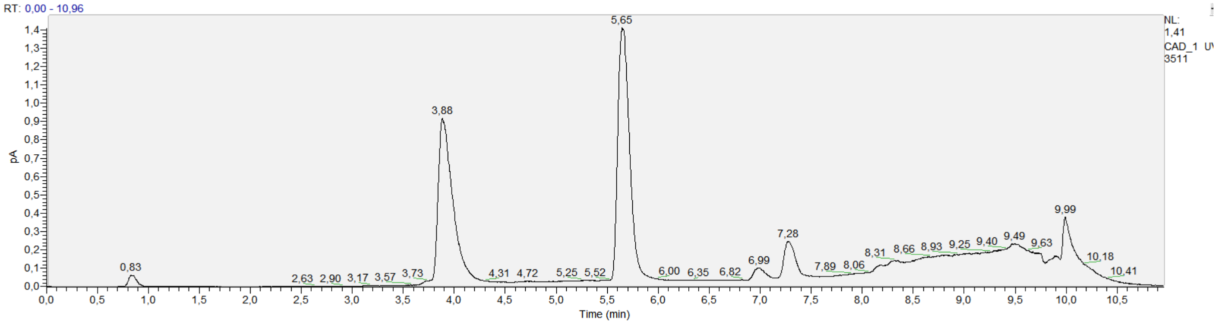

^{1}H NMR spectrum of (S)-N-ethyl-1-(4-fluorophenyl)-1-(2-(4-(6-(1-methyl-1H-pyrazol-4-yl)pyrrolo[2,1-f][1,2,4]triazin-4-yl)piperazin-1-yl)pyrimidin-5-yl)ethan-1-amine (**9**).

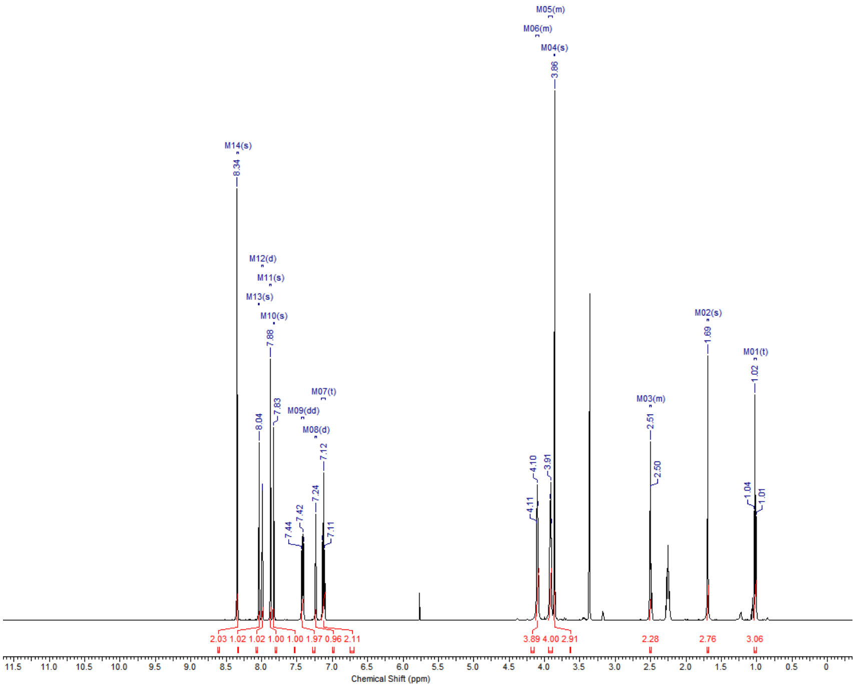

^{13}C NMR spectrum of (S)-N-ethyl-1-(4-fluorophenyl)-1-(2-(4-(6-(1-methyl-1H-pyrazol-4-yl)pyrrolo[2,1-f][1,2,4]triazin-4-yl)piperazin-1-yl)pyrimidin-5-yl)ethan-1-amine (**9**).

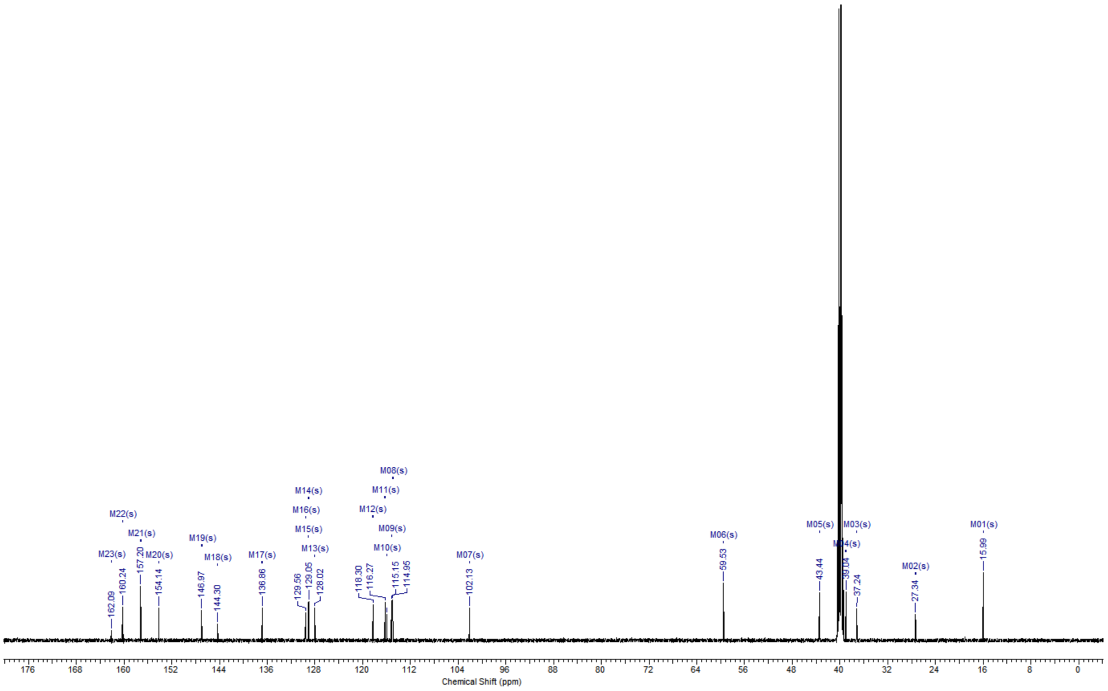

HPLC spectrum of (S)-N-ethyl-1-(4-fluorophenyl)-1-(2-(4-(6-(1-methyl-1H-pyrazol-4-yl)pyrrolo[2,1-f][1,2,4]triazin-4-yl)piperazin-1-yl)pyrimidin-5-yl)ethan-1-amine (**9**).

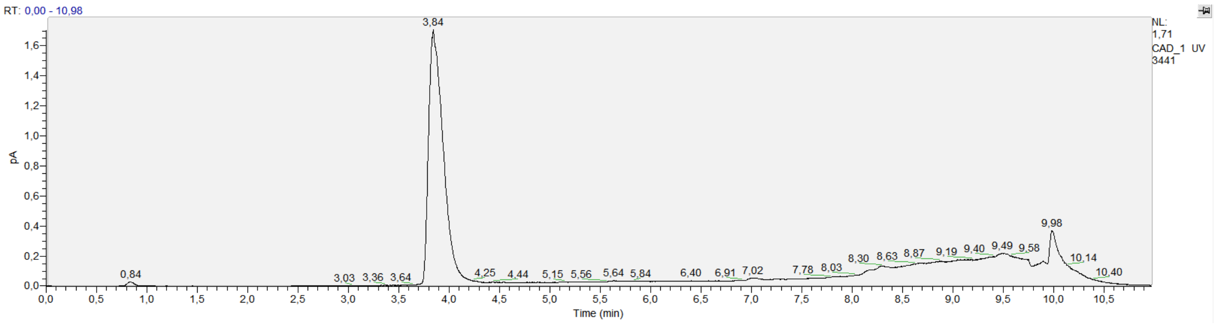

[1]*H NMR spectrum of (S)-N-(1-(4-fluorophenyl)-1-(2-(4-(6-(1-methyl-1H-pyrazol-4-yl)pyrrolo[2,1-f][1,2,4]triazin-4-yl)piperazin-1-yl)pyrimidin-5-yl)ethyl)acetamide* (***10***).

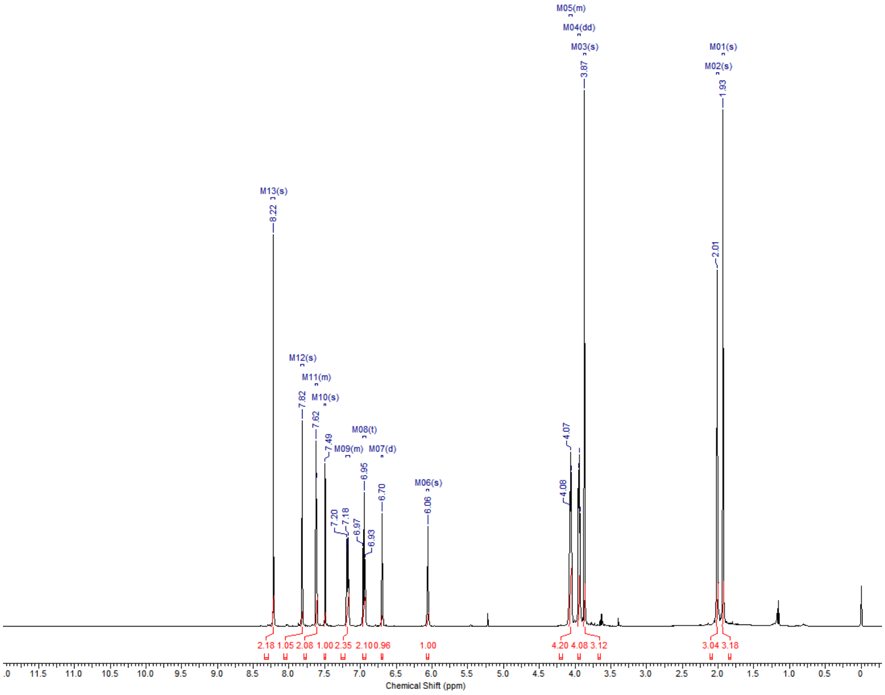

*^{13}C NMR spectrum of (S)-N-(1-(4-fluorophenyl)-1-(2-(4-(6-(1-methyl-1H-pyrazol-4-yl)pyrrolo[2,1-f][1,2,4]triazin-4-yl)piperazin-1-yl)pyrimidin-5-yl)ethyl)acetamide (**10**).*

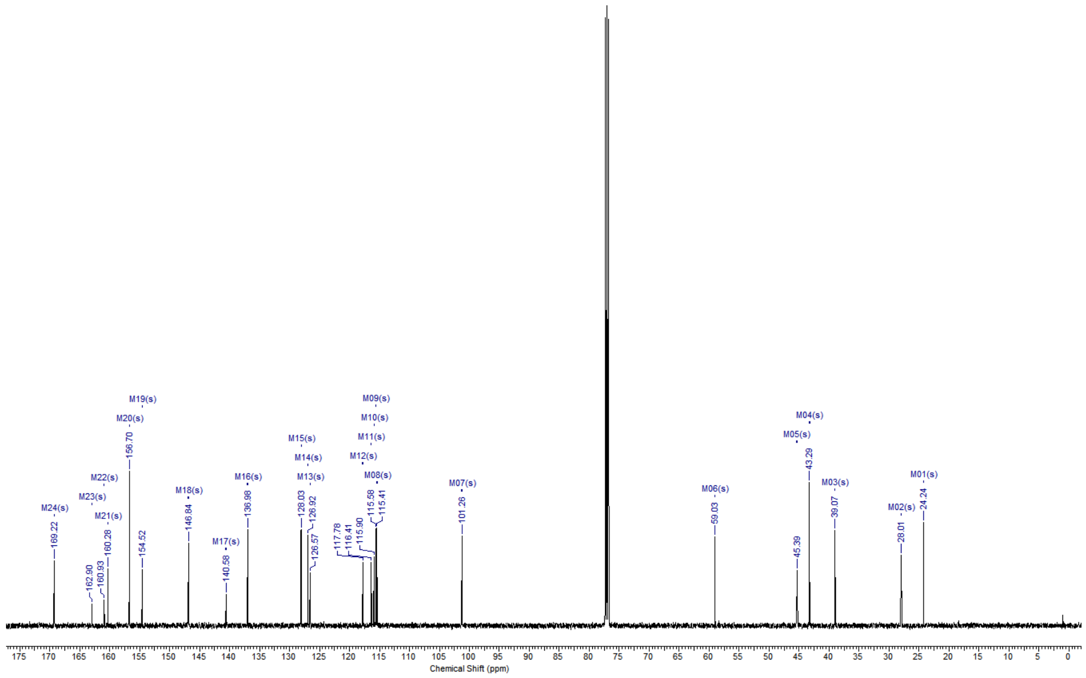

*HPLC spectrum of (S)-N-(1-(4-fluorophenyl)-1-(2-(4-(6-(1-methyl-1H-pyrazol-4-yl)pyrrolo[2,1-f][1,2,4]triazin-4-yl)piperazin-1-yl)pyrimidin-5-yl)ethyl)acetamide (**10**).*

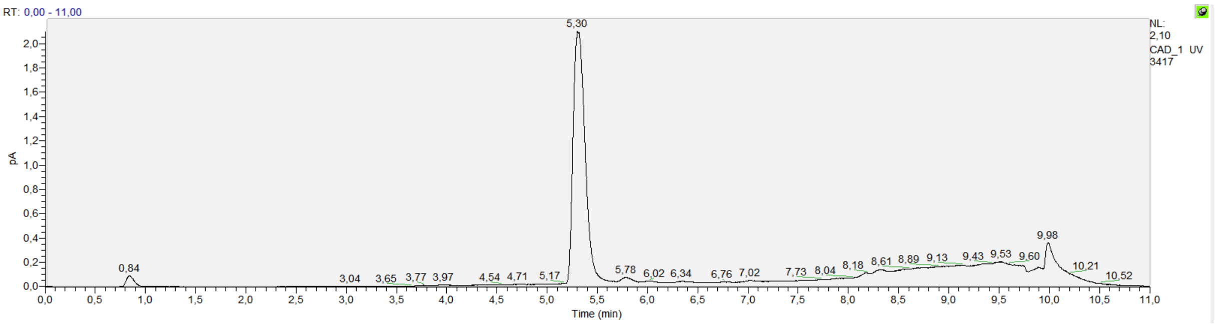

^{1}H NMR spectrum of (S)-N-(1-(4-fluorophenyl)-1-(2-(4-(6-(1-methyl-1H-pyrazol-4-yl)pyrrolo[2,1-f][1,2,4]triazin-4-yl)piperazin-1-yl)pyrimidin-5-yl)ethyl)acrylamide (**11**).

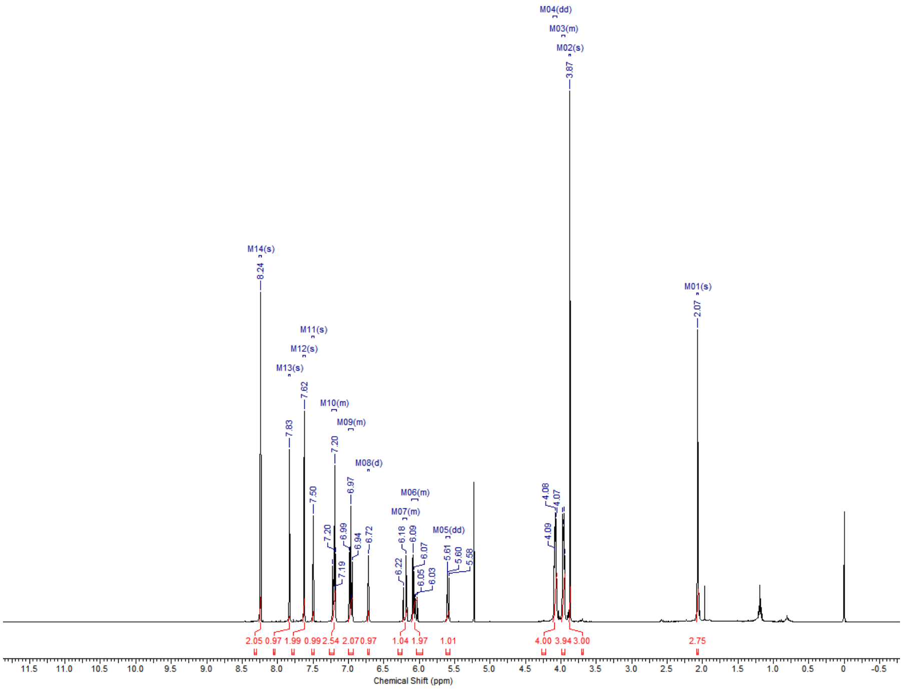

^{13}C NMR spectrum of (S)-N-(1-(4-fluorophenyl)-1-(2-(4-(6-(1-methyl-1H-pyrazol-4-yl)pyrrolo[2,1-f][1,2,4]triazin-4-yl)piperazin-1-yl)pyrimidin-5-yl)ethyl)acrylamide (**11**).

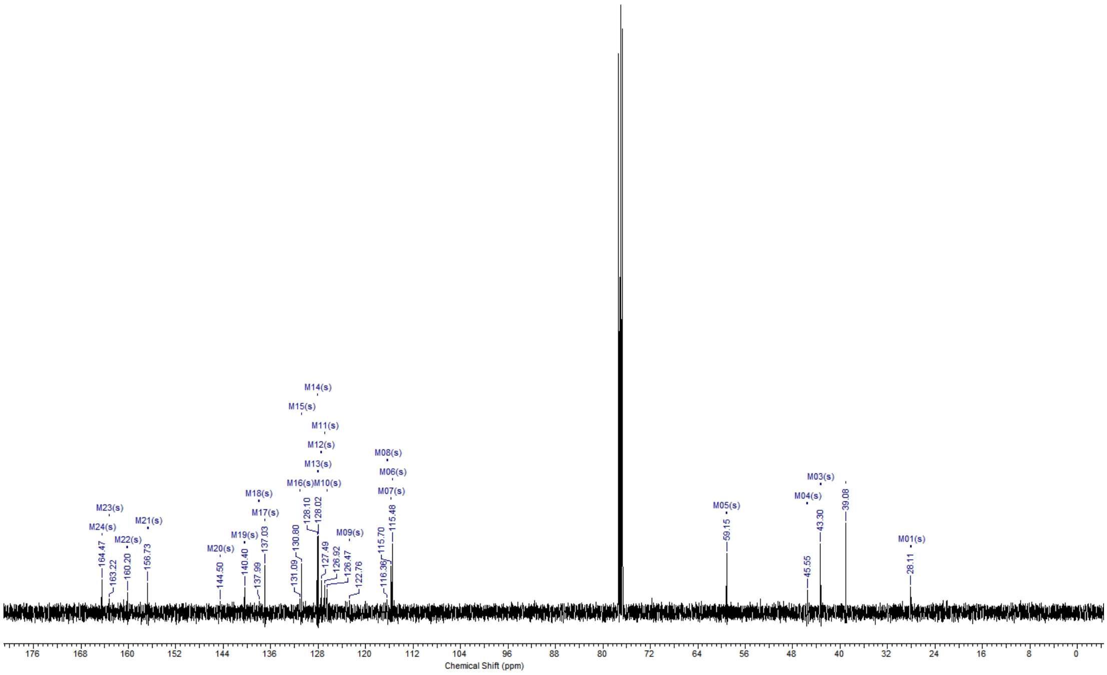

*HPLC spectrum of (S)-N-(1-(4-fluorophenyl)-1-(2-(4-(6-(1-methyl-1H-pyrazol-4-yl)pyrrolo[2,1-f][1,2,4]triazin-4-yl)piperazin-1-yl)pyrimidin-5-yl)ethyl)acrylamide (**11**).*

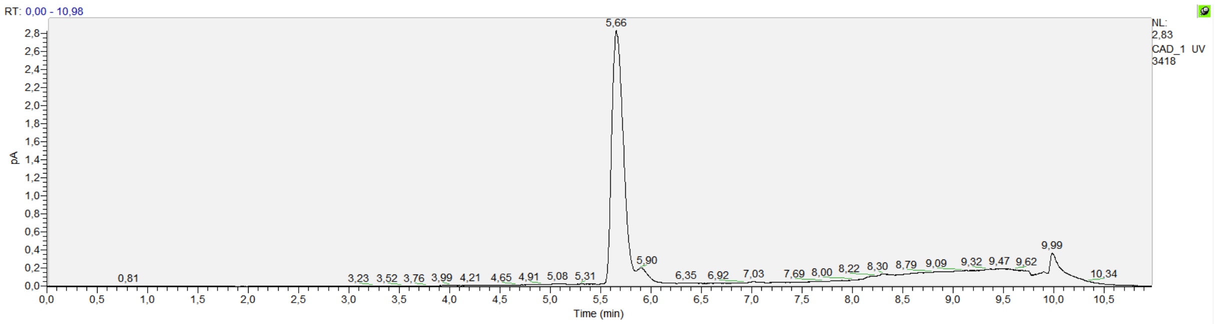

*^{1}H NMR spectrum of Methyl (S)-(1-(4-fluorophenyl)-1-(2-(4-(6-(1-methyl-1H-pyrazol-4-yl)pyrrolo[2,1-f][1,2,4]triazin-4-yl)piperazin-1-yl)pyrimidin-5-yl)ethyl)carbamate (**12**).*

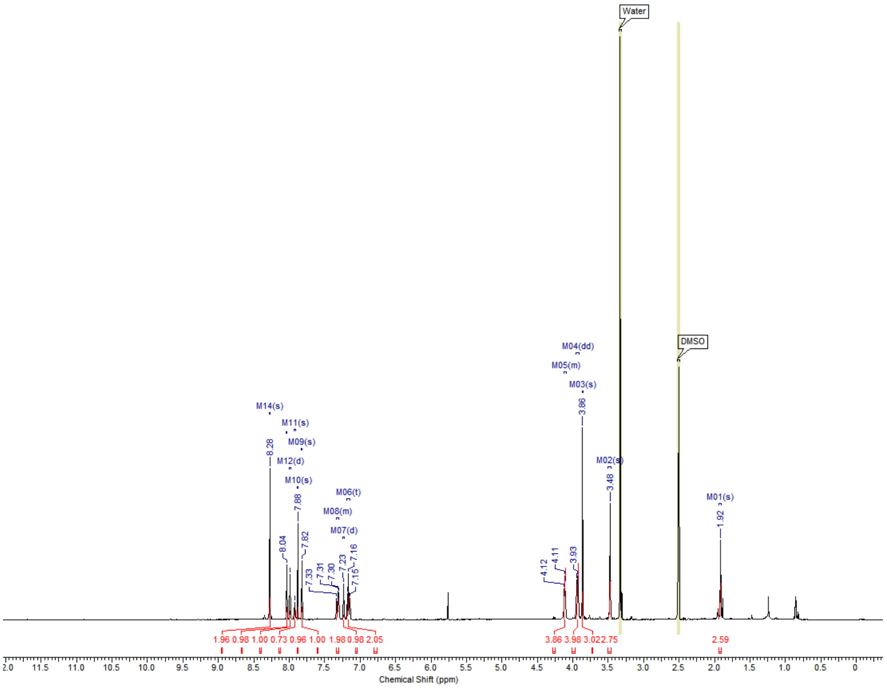

*^{13}C NMR spectrum of Methyl (S)-(1-(4-fluorophenyl)-1-(2-(4-(6-(1-methyl-1H-pyrazol-4-yl)pyrrolo[2,1-f][1,2,4]triazin-4-yl)piperazin-1-yl)pyrimidin-5-yl)ethyl)carbamate (**12**).*

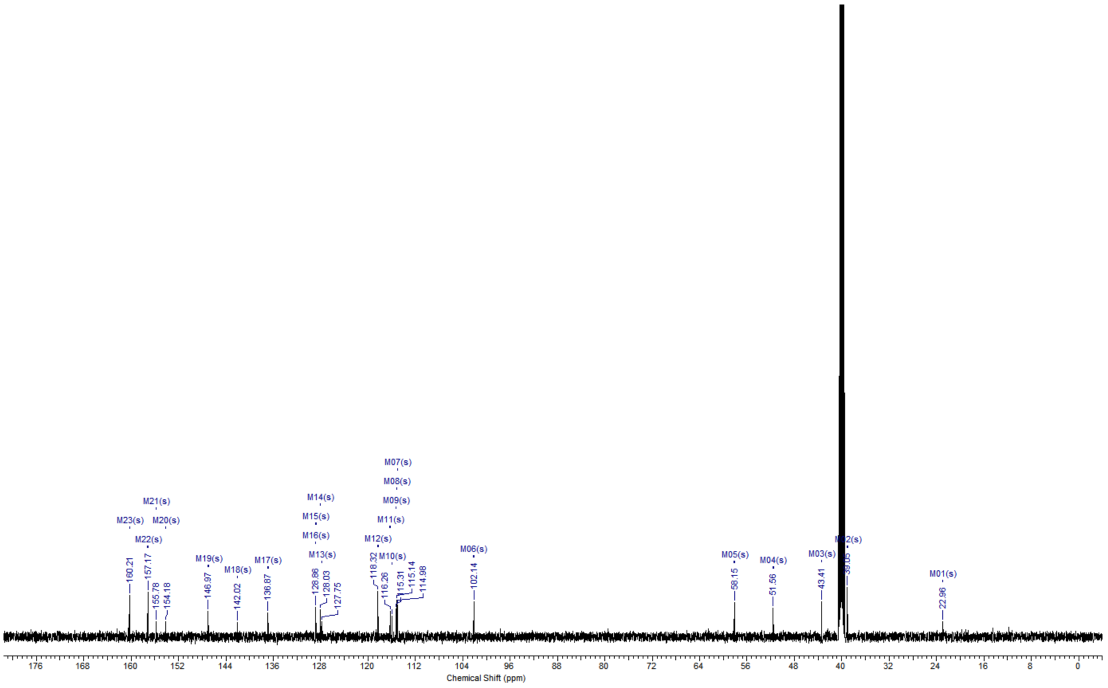

*HPLC spectrum of Methyl (S)-(1-(4-fluorophenyl)-1-(2-(4-(6-(1-methyl-1H-pyrazol-4-yl)pyrrolo[2,1-f][1,2,4]triazin-4-yl)piperazin-1-yl)pyrimidin-5-yl)ethyl)carbamate (**12**).*

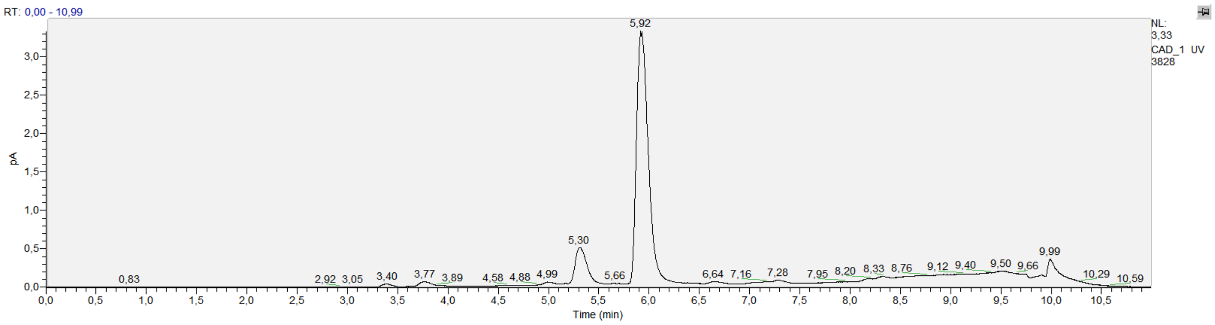

*^{1}H NMR spectrum of (S)-N-(1-(4-fluorophenyl)-1-(2-(4-(6-(1-methyl-1H-pyrazol-4-yl)pyrrolo[2,1-f][1,2,4]triazin-4-yl)piperazin-1-yl)pyrimidin-5-yl)ethyl)benzamide (**13**).*

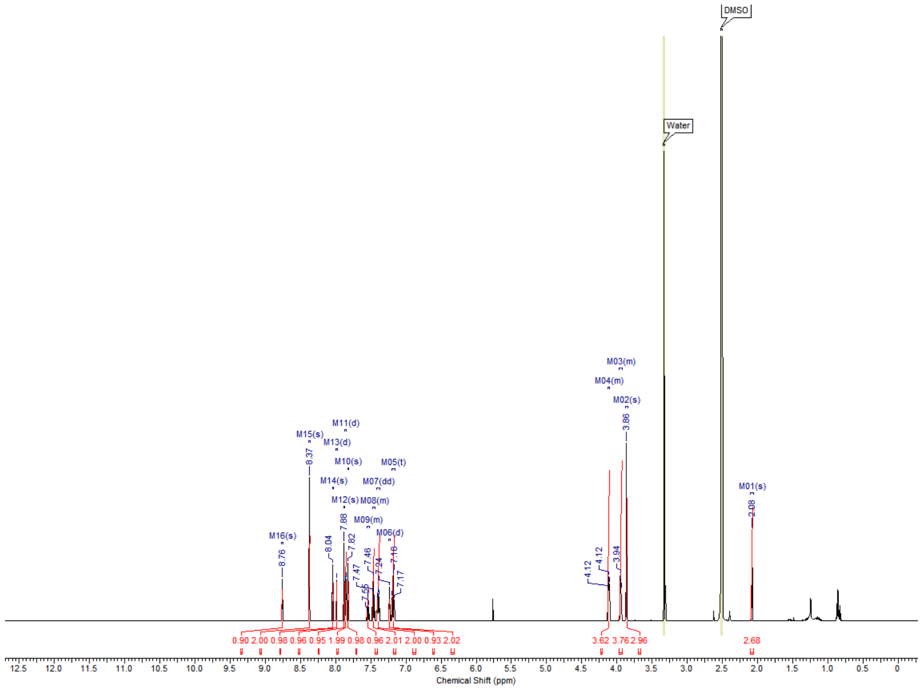

*^{13}C NMR spectrum of (S)-N-(1-(4-fluorophenyl)-1-(2-(4-(6-(1-methyl-1H-pyrazol-4-yl)pyrrolo[2,1-f][1,2,4]triazin-4-yl)piperazin-1-yl)pyrimidin-5-yl)ethyl)benzamide (**13**).*

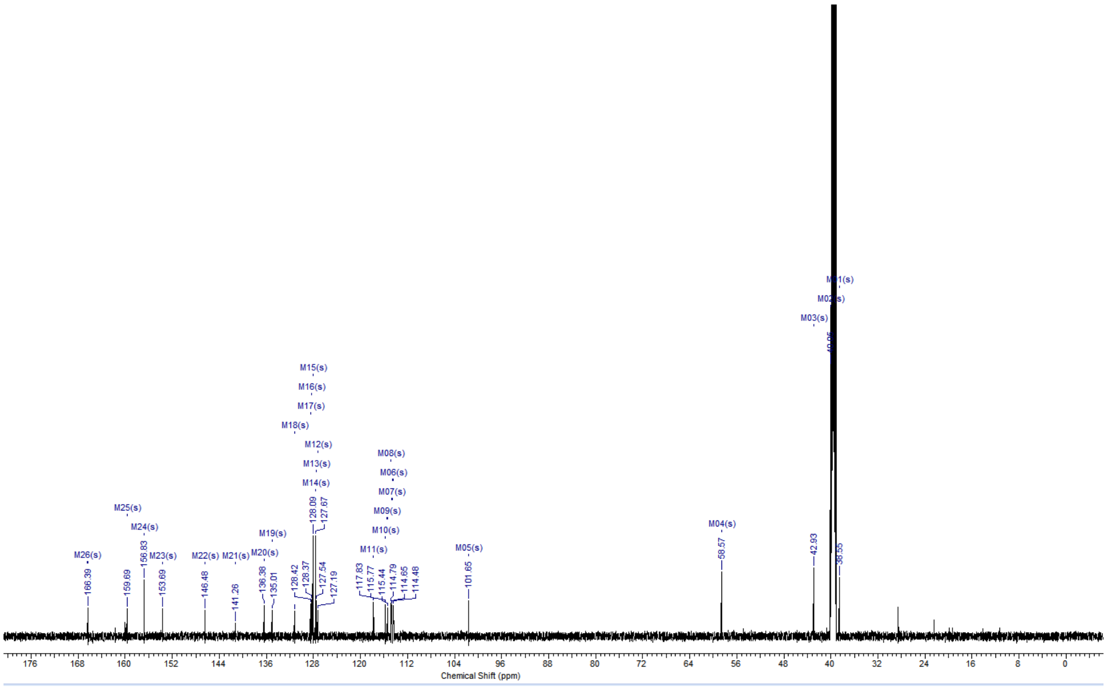

*HPLC spectrum of (S)-N-(1-(4-fluorophenyl)-1-(2-(4-(6-(1-methyl-1H-pyrazol-4-yl)pyrrolo[2,1-f][1,2,4]triazin-4-yl)piperazin-1-yl)pyrimidin-5-yl)ethyl)benzamide (**13**).*

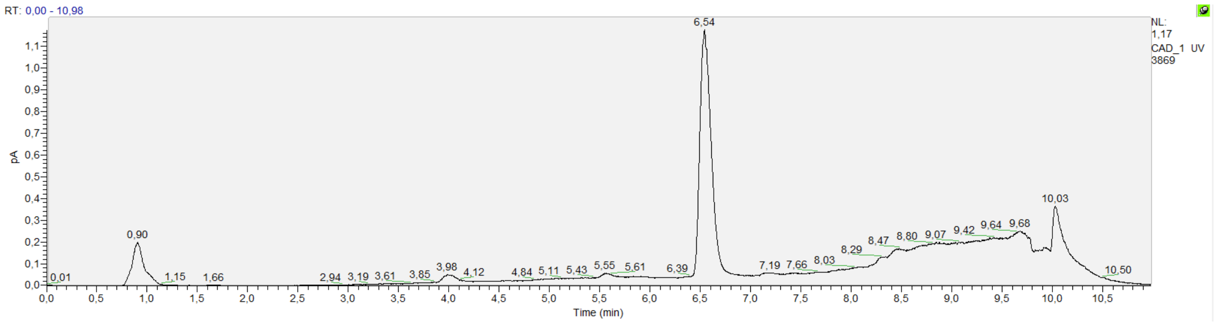

Supplementary Note 2: Abbreviations

AL	Activation loop
BBB	Blood-brain barrier
CNS-MPO	Central nervous system multiparameter optimization
CTG	CellTiter-Glo®
DFG	Aspartate-phenylalanine-glycine
GIST	Gastrointestinal stromal tumors
KIT	Stem cell factor receptor
MD	Molecular dynamics
MDCKII-MDR1	Madin Darby canine kidney cells overexpressing the multidrug resistance protein 1
PDGFRA	Platelet-derived growth factor receptor alpha
SAR	Structure-activity relationship
TKI	Tyrosine kinase inhibitor

References

1. Read RJ. Pushing the boundaries of molecular replacement with maximum likelihood. *Acta Crystallogr D Biol Crystallogr* **57**, 1373-1382 (2001).

2. Emsley P, Cowtan K. Coot: model-building tools for molecular graphics. *Acta Crystallogr D Biol Crystallogr* **60**, 2126-2132 (2004).

3. Adams PD, *et al.* PHENIX: a comprehensive Python-based system for macromolecular structure solution. *Acta Crystallogr D Biol Crystallogr* **66**, 213-221 (2010).

4. Smart OS, Womack, T. O., Sharff, A., Flensburg, C., Keller, P., Paciorek, W., Vonrhein, C. and Bricogne, G. Grade, version 1.2.20., (2011).

5. Grunewald S, *et al.* Resistance to Avapritinib in PDGFRA-Driven GIST Is Caused by Secondary Mutations in the PDGFRA Kinase Domain. *Cancer Discov* **11**, 108-125 (2021).

6. Mühlenberg T, *et al.* KIT-Dependent and KIT-Independent Genomic Heterogeneity of Resistance in Gastrointestinal Stromal Tumors - TORC1/2 Inhibition as Salvage Strategy. *Mol Cancer Ther* **18**, 1985-1996 (2019).

7. Liang X, *et al.* The synthesis review of the approved tyrosine kinase inhibitors for anticancer therapy in 2015-2020. *Bioorg Chem* **113**, 105011 (2021).
