# Supplementary material for: Avapritinib-based SAR studies unveil a binding pocket in KIT and PDGFRA
Source: Nat Commun. 2024 Jan 2;15:63. doi: 10.1038/s41467-023-44376-8 (PMC10761696; doi:10.1038/s41467-023-44376-8)
Supplement: Supplementary file 3 — Reporting Summary [file 41467_2023_44376_MOESM3_ESM.pdf]

nature portfolio

Corresponding author(s): Daniel Rauh

Last updated by author(s): Nov 28, 2023

Reporting Summary

Nature Portfolio wishes to improve the reproducibility of the work that we publish. This form provides structure for consistency and transparency in reporting. For further information on Nature Portfolio policies, see our Editorial Policies and the Editorial Policy Checklist.

Statistics

For all statistical analyses, confirm that the following items are present in the figure legend, table legend, main text, or Methods section.

n/a	Confirmed	
☐	☒	The exact sample size (*n*) for each experimental group/condition, given as a discrete number and unit of measurement
☐	☒	A statement on whether measurements were taken from distinct samples or whether the same sample was measured repeatedly
☒	☐	The statistical test(s) used AND whether they are one- or two-sided<br>*Only common tests should be described solely by name; describe more complex techniques in the Methods section.*
☒	☐	A description of all covariates tested
☒	☐	A description of any assumptions or corrections, such as tests of normality and adjustment for multiple comparisons
☐	☒	A full description of the statistical parameters including central tendency (e.g. means) or other basic estimates (e.g. regression coefficient) AND variation (e.g. standard deviation) or associated estimates of uncertainty (e.g. confidence intervals)
☒	☐	For null hypothesis testing, the test statistic (e.g. *F*, *t*, *r*) with confidence intervals, effect sizes, degrees of freedom and *P* value noted<br>*Give P values as exact values whenever suitable.*
☒	☐	For Bayesian analysis, information on the choice of priors and Markov chain Monte Carlo settings
☒	☐	For hierarchical and complex designs, identification of the appropriate level for tests and full reporting of outcomes
☒	☐	Estimates of effect sizes (e.g. Cohen's *d*, Pearson's *r*), indicating how they were calculated

Our web collection on statistics for biologists contains articles on many of the points above.

Software and code

Policy information about availability of computer code

Data collection	NMR data collected with: Bruker AVANCE III HD 400/500/600/700 MHz, Agilent DD2 500 MHz<br>Fluorescent data collected with: Spark Plate reader (Tecan), EnVision (PerkinElmer)<br>WB read out (chemiluminescence): LAS3000 (FUJI) with software Science Lab 2001 ImageGauge 4.0<br>MDCKII-MDR1 read out: Shimadzu UFLC XR-System coupled to QTrap 5500 (Sciex)<br>MS-data collected: Thermo Fisher Scientific Velos Pro, LCQ Advantage Max (1200 series, Agilent), Thermo LTQ Orbitrap<br>X-Ray crystallographic data was collected at the SLS, Paul-Scherrer-Institut, Switzerland and at the ESRF, Grenoble, France<br>in silico calculation: Marvin 22.13.0, Chemaxon (https://www.chemaxon.com)
Data analysis	All software used in the current study were commercially or publicly available and described in the methods section.<br>NMR data were analysed with ACD/Labs 12.01 (Advanced Chemistry Development Inc.)<br>HTRF and CTG data were analyzed with Quattro Software Suite 11.2.0.3 or R-package Grmetrics<br>MS data were evaluated with Xcalibur 2.2.48, deconvoluted with MagTran v1.02<br>MDCKII-MDR1 data analysis: Analyst 1.6.2<br>Calculation of Papp-values: Microsoft Excel<br>X-Ray crystallographic data were analysed with the software packages XDS (VERSION Jan 10, 2022 BUILT=20220820) and WinCoot 0.8.9.1, PHASER (integrated in Phenix), Phenix (1.20.1-4487), and visualized with PyMOL 2.0.6<br>generation of pharmacophore models: LigandScout (v. 4.4.8)

For manuscripts utilizing custom algorithms or software that are central to the research but not yet described in published literature, software must be made available to editors and reviewers. We strongly encourage code deposition in a community repository (e.g. GitHub). See the Nature Portfolio guidelines for submitting code & software for further information.

Data

Policy information about availability of data

All manuscripts must include a data availability statement. This statement should provide the following information, where applicable:

- Accession codes, unique identifiers, or web links for publicly available datasets
- A description of any restrictions on data availability
- For clinical datasets or third party data, please ensure that the statement adheres to our policy

The data supporting the findings of this study are available in the paper and its Supplementary Information. Source Data are provided with this paper. The crystal structure data generated in this study have been deposited in the PDB database under accession codes 8PQ9 [https://doi.org/10.2210/pdb8pq9/pdb], 8PQA [https://doi.org/10.2210/pdb8pqa/pdb], 8PQB [https://doi.org/10.2210/pdb8pqb/pdb], 8PQC [https://doi.org/10.2210/pdb8pqc/pdb], 8PQD [https://doi.org/10.2210/pdb8pqd/pdb], 8PQE [https://doi.org/10.2210/pdb8pqe/pdb], 8PQF [https://doi.org/10.2210/pdb8pqf/pdb], 8PQG [https://doi.org/10.2210/pdb8pqg/pdb], 8PQH [https://doi.org/10.2210/pdb8pqh/pdb], 8PQI [https://doi.org/10.2210/pdb8pqi/pdb], 8PQJ [https://doi.org/10.2210/pdb8pqj/pdb], 8PQK [https://doi.org/10.2210/pdb8pqk/pdb]. Original diffraction data can be accessed on https://www.proteindiffraction.org/ under the same accession codes. Already reported structures are deposited under following accession codes: 1T46 [https://doi.org/10.2210/pdb1t46/pdb], 6GQK [https://doi.org/10.2210/pdb6gqk/pdb], 6GQM [https://doi.org/10.2210/pdb6gqm/pdb], 6GQL [https://doi.org/10.2210/pdb6gql/pdb], 1PKG [https://doi.org/10.2210/pdb1pkg/pdb], 3G0E [https://doi.org/10.2210/pdb3g0e/pdb], 4U0I [https://doi.org/10.2210/pdb4u0i/pdb], 6MOB [https://doi.org/10.2210/pdb6mob/pdb], 7KHK [https://doi.org/10.2210/pdb7khk/pdb], 7KHJ [https://doi.org/10.2210/pdb7khj/pdb] and 5GRN [https://doi.org/10.2210/pdb15grn/pdb]. Of note, these structures have been solved by other groups and first published elsewhere.

Research involving human participants, their data, or biological material

Policy information about studies with human participants or human data. See also policy information about sex, gender (identity/presentation), and sexual orientation and race, ethnicity and racism.

Reporting on sex and gender	Not applicable
Reporting on race, ethnicity, or other socially relevant groupings	Not applicable
Population characteristics	Not applicable
Recruitment	Not applicable
Ethics oversight	Not applicable

Note that full information on the approval of the study protocol must also be provided in the manuscript.

Field-specific reporting

Please select the one below that is the best fit for your research. If you are not sure, read the appropriate sections before making your selection.

☒ Life sciences ☐ Behavioural & social sciences ☐ Ecological, evolutionary & environmental sciences

For a reference copy of the document with all sections, see nature.com/documents/nr-reporting-summary-flat.pdf

Life sciences study design

All studies must disclose on these points even when the disclosure is negative.

Sample size	For HTRF assay the sample size was determined based on the manufacturer’s protocol. For assay setup, necessary amount of kinase, ATP, substrate and fluorophore was measured for each kinase based on manufacturer's protocol to a total volume of 20 µL in each well. Briefly: 5 µL Kinase solutionand 2.5 µL inhibitor solution, the reaction was started by addition of 2.5 µL starting solution containing ATP and substrate peptide (at K(M) values each). After reaction completion, 10 µL of stop solution were added. Measurements were done by a concentration of eight repetitive bisecting concentrations. For cell viability CTG assay the sample size was determined using a concentration series of eight repetitive bisecting concentrations. The optimal cell number for the cell viability assay was determined from the linear growth range of each cell line. For western blot sample preparation the number of seeded cells was based on prior experience and experiments. No statistical method was used to predetermine sample size.
Data exclusions	No data were excluded.
Replication	HTRF assays were succsessfully replicated at least three times with two technical replicates in each experiment.CTG assays of sarcoma cell lines were succsessfully replicated at least three times with three technical replicates in each experiment. CTG assays of breast cancer cell lines were successfully replicated at least three times with two technical replicates in each experiment. MDCKII-MDR1 assays were performed three times. Qualitative Western blots were performed once.
Randomization	For in vitro studies, the samples/cells were randomized into different groups prior to treatment.
Blinding	Investigators were not blinded to the experiments as the researchers need to rank and load the samples based on the treatment information.

Blinding was not relevant for biochemical in vitro experiments we used manufactured enzymes and known substrates.

Reporting for specific materials, systems and methods

We require information from authors about some types of materials, experimental systems and methods used in many studies. Here, indicate whether each material, system or method listed is relevant to your study. If you are not sure if a list item applies to your research, read the appropriate section before selecting a response.

Materials & experimental systems

n/a	Involved in the study
☐	☒ Antibodies
☐	☒ Eukaryotic cell lines
☒	☐ Palaeontology and archaeology
☒	☐ Animals and other organisms
☒	☐ Clinical data
☒	☐ Dual use research of concern
☒	☐ Plants

Methods

n/a	Involved in the study
☒	☐ ChIP-seq
☒	☐ Flow cytometry
☒	☐ MRI-based neuroimaging

Antibodies

Antibodies used

Anti-tErk1/2 (CST, order. no. 9102, 1:1000), anti-pERK1/2(Thr202/Tyr204) (order no. 9101, 1:1000), anti-tAKT (CST, order no. 9272, 1:1000), anti-pAKT(Ser473) (CST, order no. 9271, 1:1000), anti-S6 (CST, order no. 2217, 1:1000), anti-pS6 (Ser235/236) (CST, order no. 2211, 1:1000), anti-tPDGFRA (CST, order no. 3174, 1:1000), anti-pPDGFRA (Tyr849)/PDGFRB (Tyr857) (CST, order no. 3170, 1:1000), anti-beta-Actin (CST, order no. 3700, 1:1000), secondary antibody anti-rabbit IgG, HRP-linked antibody (CST, order no. 7074, 1:2000), secondary antibody anti-mouse IgG, HRP-linked antibody (CST, order no. 7076, 1:2000)

Validation

Anti-tErk1/2 (CST, order. no. 9102, 1:1000):
Specificity / Sensitivity: p44/42 MAPK (Erk1/2) Antibody detects endogenous levels of total p44/42 MAP kinase (Erk1/Erk2) protein. In some cell types, this antibody recognizes p44 MAPK more readily than p42 MAPK. The antibody does not recognize either JNK/SAPK or p38 MAP kinase.
Species Reactivity: Human, Mouse, Rat, Hamster, Monkey, Mink, Zebrafish, Bovine, Pig, S. cerevisiae.

anti-pERK1/2(Thr202/Tyr204) (order no. 9101, 1:1000):
Specificity / Sensitivity: Phospho-p44/42 MAPK (Erk1/2) (Thr202/Tyr204) Antibody detects endogenous levels of p44 and p42 MAP Kinase (Erk1 and Erk2) when phosphorylated either individually or dually at Thr202 and Tyr204 of Erk1 (Thr185 and Tyr187 of Erk2). The antibody does not cross-react with the corresponding phosphorylated residues of either JNK/SAPK or p38 MAP Kinase, and does not cross-react with non-phosphorylated Erk1/2.
Species Reactivity: Human, Mouse, Rat, Hamster, Monkey, Mink, D. melanogaster, Zebrafish, Bovine, Pig, C. elegans
Species predicted to react based on 100% sequence homology: Chicken.

anti-tAKT (CST, order no. 9272, 1:1000):
Specificity / Sensitivity: Akt Antibody detects endogenous levels of total Akt1, Akt2 and Akt3 proteins. The antibody does not cross-react with related kinases.
Species Reactivity: Human, Mouse, Rat, Hamster, Monkey, Chicken, D. melanogaster, Bovine, Dog, Pig, Guinea Pig
Species predicted to react based on 100% sequence homology: Dog.

anti-pAKT(Ser473) (CST, order no. 9271, 1:1000):
Specificity / Sensitivity: Phospho-Akt (Ser473) Antibody detects endogenous levels of Akt1 only when phosphorylated at Ser473. This antibody also recognizes Akt2 and Akt3 when phosphorylated at the corresponding residues. It does not recognize Akt phosphorylated at other sites, nor does it recognize phosphorylated forms of related kinases such as PKC or p70 S6 kinase.
Species Reactivity: Human, Mouse, Rat, Hamster, Monkey, D. melanogaster, Bovine, Dog
Species predicted to react based on 100% sequence homology: Monkey, Chicken, Xenopus, Horse.

anti-S6 (CST, order no. 2217, 1:1000):
Specificity / Sensitivity: S6 Ribosomal Protein (5G10) Rabbit Monoclonal Antibody detects endogenous levels of total S6 ribosomal protein independent of phosphorylation.
Species Reactivity: Human, Mouse, Rat, Monkey
Species predicted to react based on 100% sequence homology: Pig.

anti-pS6 (Ser235/236) (CST, order no. 2211, 1:1000):
Specificity / Sensitivity: Phospho-S6 Ribosomal Protein (Ser235/236) Antibody detects endogenous levels of ribosomal protein S6 only when phosphorylated at serine 235 and 236. This antibody does not detect ribosomal protein S6 phosphorylated at other sites.
Species Reactivity: Human, Mouse, Rat, Monkey, S. cerevisiae
Species predicted to react based on 100% sequence homology: Chicken, Xenopus.

anti-tPDGFRA (CST, order no. 3174, 1:1000):
Specificity / Sensitivity: PDGF Receptor α (D1E1E) XP® Rabbit mAb detects endogenous levels of PDGFRα. This antibody may cross-react with PDGFRβ at overexpressed levels. Nuclear staining has been observed with this antibody in certain tissues. The specificity of this staining is unknown.
Species Reactivity: Human, Mouse.

anti-pPDGFRA (Tyr849)/PDGFRB (Tyr857) (CST, order no. 3170, 1:1000):
Specificity / Sensitivity: Phospho-PDGF Receptor α (Tyr849)/PDGF Receptor β (Tyr857) (C43E9) Rabbit mAb detects endogenous levels of PDGF receptor α and β only when phosphorylated on Tyr849 of PDGFRα and Tyr857 of PDGFRβ. This antibody may cross-react with other activated tyrosine kinases.
Species Reactivity: Human, Mouse, Rat.

anti-beta-Actin (CST, order no. 3700, 1:1000):
Specificity / Sensitivity: β-Actin (8H10D10) Mouse mAb detects endogenous levels of total β-actin protein. Due to the high sequence identity between the cytoplasmic actin isoforms, β-actin and cytoplasmic γ-actin, this antibody may cross-react with cytoplasmic γ-actin. It does not cross-react with α-skeletal, α-cardiac, α-vascular smooth, or γ-enteric smooth muscle isoforms.
Species Reactivity: Human, Mouse, Rat, Hamster, Monkey, Dog.

secondary antibody anti-rabbit IgG, HRP-linked antibody (CST, order no. 7074, 1:1000):
Reactivity/Sensitivity: no information provided from the manufacturer.

secondary antibody anti-mouse IgG, HRP-linked antibody (CST, order no. 7076, 1:2000):
Reactivity/Sensitivity: no information provided by the manufacturer.

Antibody validation Cell Signaling Technologies:
CST adhere to the Hallmarks of Antibody Validation™, six complementary strategies that can be used to determine the functionality, specificity, and sensitivity of an antibody in any given assay. CST adapted the work by Uhlen, et. al., ("A Proposal for Validation of Antibodies." Nature Methods (2016)) to build the Hallmarks of Antibody Validation.
Binary Model: Antibody signal is measured in model systems with known presence/absence of target signal. Includes wild-type vs. genetic knockout, targeted induction or silencing.

Ranged Expression: Antibody signal strength is measured in cell lines or tissues representing a known continuum of target expression levels. Includes siRNA and heterozygous knockout assays.

Orthogonal Data: Antibody signal is correlated to target expression in model systems measured using antibody independent assays. Includes mass spectrometry and in situ hybridization.

Multiple Antibodies: Antibody signal is compared to the signal observed using antibodies targeting nonoverlapping epitopes of the target. Includes IP, ChIP, and ChIP-seq.

Heterologous Expression: Antibody signal is evaluated in cell lines following heterologous expression of native (or mutated) target protein.

Complementary Assays: Antibody specificity may be validated using complementary assays. Includes competitive ELISA, peptide dot blots, peptide blocking, or protein arrays.

Eukaryotic cell lines

Policy information about cell lines and Sex and Gender in Research

Cell line source(s)

GIST-T1 was established from human metastatic GIST (japanese woman). GIST-T1 (RRID:CVCL_4976, DOI:10.1038/labinvest.3780461) contains a 57-bp deletion in KIT exon 11 and was established by Takahiro Taguchi (Kochi University, Kochi, Japan).
T1-D816E (RRID: CVCL_A9N0) and T1-T670I (RRID: CVCL_A9M9) (established by Brian Rubin, Cleveland Clinic, OH) were established by long term imatinib treatment of GIST-T1.
Further cell lines with endogenous PDGFRA mutations (T1-a-D842V, T1-a-D842V/G680R, T1-a-D842V/T674I/R) were generated by CRISPR/Cas9-mediated gene editing in GIST-T1.
GIST-48B (RRID: CVCL_M441), was established by Jonathan Fletcher (Brigham and Women's Hospital, Boston, MA), is isogenic subline of GIST-48 (RRID: CVCL_7041), which retains the parental oncogenic KIT genomic mutations but has lost KIT protein-level expression and is therefore KIT-independent and served as negative control.
SK-LMS-1 (RRID:CVCL_0628), was established by Jonathan Fletcher, is a leiomyosarcoma cell line not driven by any oncogenic activated kinase, it is served as an additional negative control for our studies.
ZR-75-1 (RRID:CVCL_0588) cell line was purchased from Sigma-Aldrich (ECACC) and is derived from human metastatic breast carcinoma (caucasian female), MDA-MB-174-VII (RRID:CVCL_1400) cell line was purchased from LGC Standards (ATCC) and is derived from human metastatic breast carcinoma of no special type (african american female).
MDCKII-MDR1 cells was licensed from the Netherlands Cancer Institute (Amsterdam, Netherlands) and internally expanded (Master and Working Banks).

Authentication

All GIST cell lines were regularly authenticated by sequencing for endogenous mutations in KIT, confirmation of phosphorylated KIT expression, and response to KIT inhibitor treatment. Authenticity of breast cancer cell lines was

	confirmed by STR analysis at Eurofins Genomics.
Mycoplasma contamination	In the course of this study, all sarcoma cell lines were regularly tested (every 3-4 months) for mycoplasma contamination by PCR and/or MycoAlert Mycoplasma Detection Kit (Lonza). Breast cancer cell lines were regularly tested for mycoplasma contamination using the Mycoplasmacheck Service at Eurofins Genomics.<br>MDCKII-MDR1: at the LDC, all cell lines are routinely tested for mycoplasma contamination.
Commonly misidentified lines<br>(See ICLAC register)	No commonly misidentified lines are included.
